# Haplotype-resolved genome assembly sheds light on the evolutionary history of autohexaploid *Tripidium arundinaceum*

**DOI:** 10.1038/s41467-026-74742-1

**Published:** 2026-06-27

**Authors:** Zhen Li, Yiying Qi, Xinlong Liu, Baiyu Wang, Xiuting Hua, Ruiting Gao, Qing Zhang, Gang Wang, Jin Chai, Gui Zhuang, Yixing Zhang, Hanjing Liu, Yuhao Wang, Jia Liu, Qiaoyu Wang, Nameng Qi, Huihong Shi, Yumin Huang, Xiuqin Lin, Zuhu Deng, Jisen Zhang

**Affiliations:** 1 Guangxi Sugarcane Bio-breeding Laboratory, State Key Laboratory for Conservation and Utilization of Subtropical Agro-bioresources, College of Agriculture, Guangxi University, Nanning, Guangxi China; 2 College of Plant Protection, Shandong Agricultural University, Tai’an, China; 3 Yunnan Key Laboratory of Sugarcane Genetic Improvement, Sugarcane Research Institute, Yunnan Academy of Agricultural Sciences, Kaiyuan, China; 4 Jiangsu Key Laboratory for Bioresources of Saline Soils, Yancheng Teachers University, Yancheng, China; 5 College of Agriculture, Fujian Agriculture and Forestry University, Fuzhou, China; 6 Center for Genomics and Biotechnology, National Engineering Research Center of Sugarcane, Fujian Provincial Key Laboratory of Haixia Applied Plant Systems Biology, Fujian Agriculture and Forestry University, Fuzhou, China

**Keywords:** Genome, Phylogenetics, Population genetics, Plant breeding

## Abstract

*Tripidium arundinaceum*, a wild perennial grass with exceptional stress tolerance, has been tested in sugarcane breeding. Its complex polyploid genome has hindered understanding of its evolution and adaptive traits. Here, we present a high-quality, haplotype-resolved reference genome for autohexaploid *T. arundinaceum* Hainan92-105 (2*n* = 6*x* = 60). *T. arundinaceum* is likely originated from a diploid ancestor approximately 1.31 million years ago and contains abundant repetitive sequences from two major recent bursts of long terminal repeat retrotransposons following polyploidization. Phylogenetic analyses place *Saccharum* closer to *Sorghum bicolor* than to *T. arundinaceum*, indicating that the polyploidizations in these two lineages occurred independently. Population genomics analysis reveals southwestern China (Yunnan) as a diversity center for *T. arundinaceum* within China. Genotype–environment associations highlight temperature and precipitation as key local adaptation drivers mediated by stress-associated genes. This study provides a genomic resource for understanding polyploid evolution and climate adaptation in *T. arundinaceum*.

## Abstract (web summary)

*Tripidium arundinaceum* has been tested in sugarcane breeding for its stress tolerance. Here, the authors report its haplotype resolved genome and reveal the evolutionary history leading to its autohexaploid genome.

## Introduction

*Tripidium arundinaceum* (2*n* = 4*x* = 40 or 2*n* = 6*x* = 60), a wild perennial grass, was originally classified within the genera *Saccharum*^1,2^ or *Erianthus*^3,4^, both of which belong to the “*Saccharum* complex”, an interbreeding group of species closely associated with the origin and improvement of modern cultivated sugarcane^5,6^. However, Lloyd Evans et al.^7^ conducted comprehensive phylogenetic analyses based on both whole chloroplast genomes and low-copy-number gene loci, demonstrating that *T. arundinaceum* should be reassigned to the genus *Tripidium*, which forms a monophyletic lineage distinct from both *Saccharum* and *Erianthus*. This conclusion has been independently supported by the plastome phylogenomic analysis of Welker et al.^8^. Moreover, Lloyd Evans et al.^7^ redefined the “*Saccharum* complex” to include only genera capable of interbreeding in the wild—namely *Saccharum*, *Miscanthus*, *Miscanthidium*, and New World *Erianthus* species—thereby excluding *Tripidium* from this group.

*T. arundinaceum* possesses numerous agronomically valuable traits, including a robust root system with deep rhizomes, strong ratooning ability, and exceptional adaptability to abiotic stresses such as drought, waterlogging, poor soil fertility, as well as resistance to pests and diseases^9–12^. Its deep rhizomes contribute to improve water and nutrient use efficiency compared to members of the genus *Saccharum*^12^. Given these characteristics, *T. arundinaceum* has been considered a promising native bioenergy crop with high biomass yield and cellulose content, as well as potential for environmental remediation, including ecological restoration, soil and water conservation, and contaminated land repair^13^. Additionally, these characteristics have motivated numerous attempts to use *T. arundinaceum* in sugarcane improvement programs. However, producing F₁ and backcross hybrids between sugarcane and *T. arundinaceum* remains rather difficult, with only a few cases validated by molecular evidence^14–18^. To date, no natural hybrids between *T. arundinaceum* and sugarcane have been reported, and only very few commercially grown sugarcane cultivars have been conclusively shown to carry chromosomes derived from *T. arundinaceum* (e.g., YueTang15491 and YueTang15863); moreover, these cultivars are grown over very limited areas.

Despite its considerable ecological and economic importance, genomic resources for *T. arundinaceum* remain limited, and the genetic mechanisms underlying its key adaptive traits are poorly understood. Photosynthetic efficiency, a key trait influencing biomass accumulation and stress tolerance, remains poorly characterized in *T. arundinaceum*, despite its phylogenetic proximity to established C_4_ species in the *Saccharum* complex.

In this work, we present a chromosome-scale genome assembly for the *T. arundinaceum* Hainan92-105 (2*n* = 6*x* = 60), elucidating its evolutionary position within the Andropogoneae, and analyzing the genetic basis of key traits relevant to both breeding improvement and ecological adaptation. Additionally, we provide the comprehensive molecular and physiological evidence supporting its classification as a C_4_ subtype species. Furthermore, we conduct resequencing analyses of 153 *T. arundinaceum* accessions to explore the genetic diversity and genomic mechanisms underlying climate adaptation in geographically distinct populations across China. Our study provides a foundation for future research on genomics-assisted adaptive evolution and the utilization of wild germplasm resources.

## Results

### Genome assembly

**Fig. 1:**
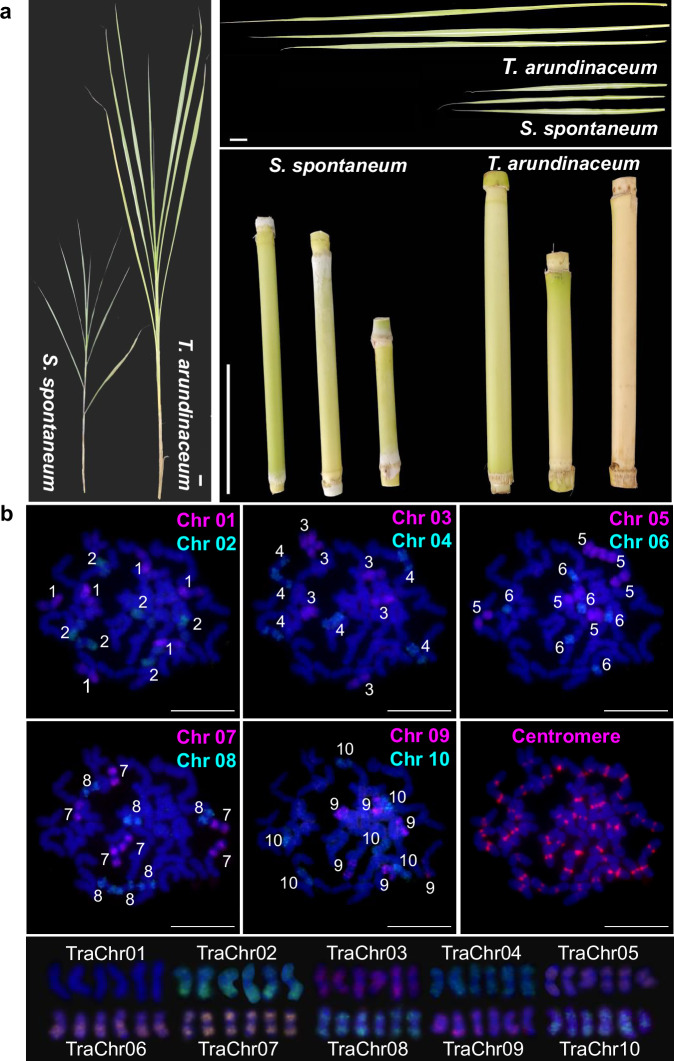
Phenotypes and karyotype characteristics of *T. arundinaceum.* **a** Comparison of phenotypes between *T. arundinaceum* and *S. spontaneum*. Scale bars, 6.5 cm. **b** FISH mapping of chromosome-specific oligo probes on the same metaphase cell of *T. arundinaceum* Hainan92-105 (2*n* = 6*x* = 60). Upper and middle panels show the chromosomal locations of Chr01-Chr10 probes and centromere repetitive probes. Oligo probes for Chr01, Chr03, Chr05, Chr07, and Chr09 are shown in magenta; probes for Chr02, Chr04, Chr06, Chr08, and Chr10 are shown in cyan. Scale bars = 10 μm. Lower panel shows karyotypes of *T. arundinaceum* Hainan92-105 Chr01 through Chr10 used to assess the chromosomes specificity of FISH probes in its genome. Tra, *T. arundinaceum*.

**Fig. 2:**
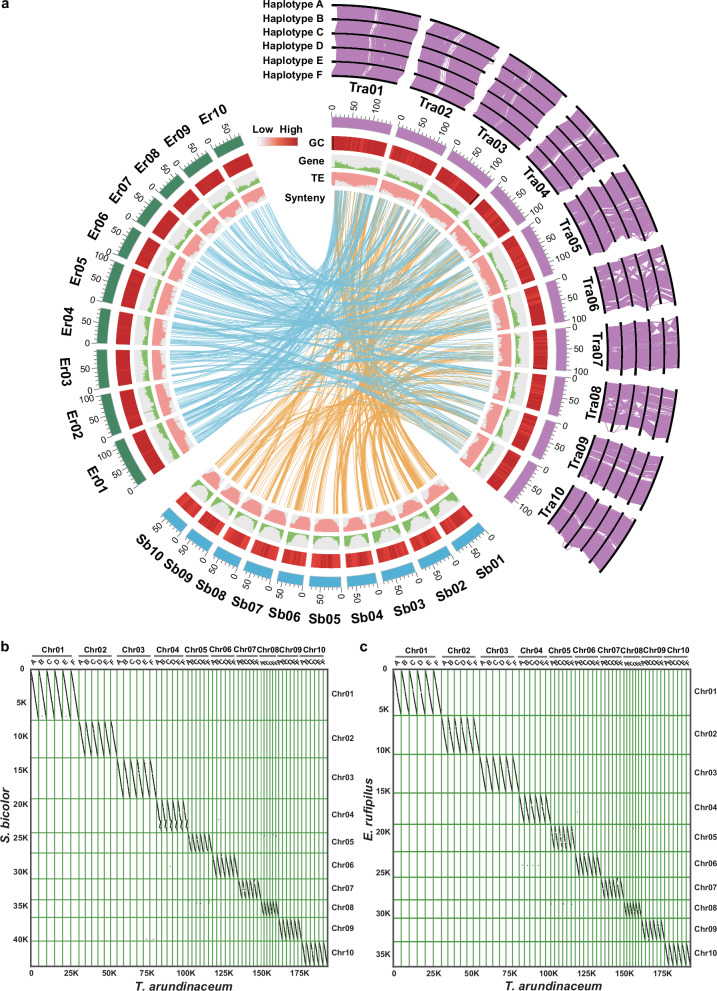
Overview of *T. arundinaceum* genome. **a** Circular schematic representation of genomic features. Conserved syntenic blocks between haplotype A, B, C, D, E, and F are indicated by purple lines. Chromosomes, GC content, gene density, transposon element density, and synteny are shown from outermost to the innermost tracks. Tra, *T. arundinaceum***;** Sb, *S. bicolor*; Er, *E. rufipilus*. **b**, **c** Genomic synteny between *T. arundinaceum* and diploid *S. bicolor* (**b**) and *E. rufipilus* (**c**). Source data are provided as a Source Data file.

In this study, we generated approximately 361.88 gigabases (Gb) of PacBio High-fidelity (HiFi) reads (comprising 123.73 Gb from older sequencing [~19× coverage] and 238.15 Gb from newly generated data [~36× coverage]), 433.85 Gb (~65× coverage) of Illumina short reads, and 636.86 Gb (~95× coverage) of Hi-C paired-end reads for the de novo assembly of the *T. arundinaceum* Hainan92-105 genome (Supplementary Tables 1 and 2). The genome size of Hainan92-105 was estimated to be ~6.73 Gb based on *k*-mer analysis of the Illumina reads (Supplementary Fig. 1). We initially assembled the HiFi reads into a contig-level assembly with an N50 of 30.60 megabases (Mb) (Table 1). Subsequent Hi-C scaffolding captured about 6.72 Gb of the genome sequence, covering 99.83% of the estimated genome size, with around 96.17% (~6.47 Gb) of the contigs anchored to 60 pseudo-chromosomes (Table 1). These pseudo-chromosomes are organized into ten homologous groups (HGs), each containing six allelic chromosomes, consistent with the karyotype of Hainan92-105 as revealed by fluorescence in situ hybridization (FISH) (Figs. 1 and 2; Supplementary Fig. 2; Supplementary Table 3). Comprehensive quality assessments confirmed a highly contiguous and complete assembly (Supplementary Table 4 and 5; Supplementary Note 1). Synteny analyses further support these findings, revealing strong conservation of syntenic blocks between the genome of *T. arundinaceum* and those of *Sorghum bicolor*, *Erianthus rufipilus*, and *Saccharum* (Fig. 2b, c; Supplementary Fig. 3). This suggests that *T. arundinaceum* and *Saccharum* likely share a last common ancestor with a base chromosome number of *x* = 10. The estimated monoploid genome size of *T. arundinaceum* is approximately 1.12 Gb, which is larger than those of *S. spontaneum* (AP85-441^19^: ~0.79 Gb; Np-X^20^: ~0.69 Gb) and *S. officinarum* (Zhongguo 1, ZG1: ~0.87 Gb; LA-purple: ~0.93 Gb) (https://sugarcane.gxu.edu.cn/scdb/download) (Supplementary Fig. 4), indicating that *T. arundinaceum* genome may have undergone expansion after its divergence from *Saccharum*.

**Table 1:** Statistics of the *T. arundinaceum* Hainan92-105 genome assembly

Genome assembly	Value
Total assembly size of contigs (bp)	6,722,710,240
No. of contigs	2097
Maximum length (bp)	90,755,854
Mean length (bp)	3,205,870
N50 contig length (bp)	30,600,032
N90 contig length (bp)	6,027,988
Total assembly size of scaffolds (bp)	6,722,760,840
No. of scaffolds	1591
Maximum length (bp)	145,881,694
Mean length (bp)	4,225,494
N50 scaffold length (bp)	104,813,120
N90 scaffold length (bp)	88,420,611
No. of chromosomes	60
Total size of chromosomes (bp)	6,465,188,122
No. of gaps	506
No. of genes	37,241
No. of gene alleles	180,467
Mean gene length (bp)	2812

### Allele-defined annotation

A total of 180,467 protein-coding genes were annotated in the Hainan92-105 genome, achieving a BUSCO completeness of 96.10% (Supplementary Table 6). Of these genes, 96.26% (173,717) have been functionally annotated (Supplementary Table 7), with 98.07% distributed across the 60 chromosomes, exhibiting slight variation among allelic chromosomes (Supplementary Fig. 5). Analysis of allele definition showed that 37,241 gene models, encompassing 158,160 alleles, were well defined. This included 16,047 gene models (43.09%) with six alleles, 5525 (14.84%) with five alleles, 3137 (8.42%) with four alleles, 2952 (7.93%) with three alleles, 3269 (8.78%) with two alleles, and 6311 (16.95%) with one allele (Supplementary Table 8). Allelic expression analysis of the 16,047 six-allele genes revealed that approximately 29.69% exhibited non-neutral expression patterns across tissues, with conserved non-neutral alleles enriched in defense- and development-related functional categories (Supplementary Figs. 6–8; Supplementary Table 9; Supplementary Note 2).

**Fig. 3:**
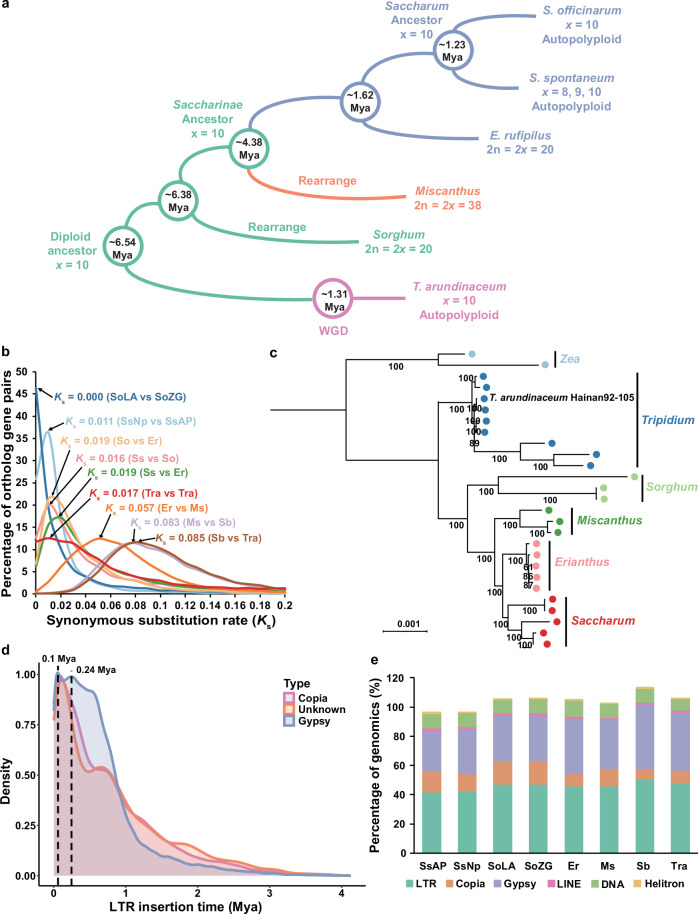
Polyploidization and evolution of *T. arundinaceum.* **a** Schematic of the evolution of *T. arundinaceum*. Divergence times were inferred from *K*_s_ values. **b** Distributions of synonymous substitution rate (*K*_s_) among the representative species *S. officinarum* (LA-purple: SoLA, ZG1: SoZG), *S. spontaneum* (AP85-441: SsAP, Np-X: SsNp), *E. rufipilus* (Er), *M. sinensis* (Ms), *S. bicolor* (Sb), and *T. arundinaceum* (Tra). **c** Unrooted phylogenetic tree based on complete chloroplast sequences of *T. arundinaceum* and related species. **d** Analysis of intact LTR-RTs insertion times in the genomes of *T. arundinaceum*. The horizontal and vertical axes represent the insertion time and density of intact LTR retrotransposons, respectively. **e** Comparison of transposable elements among *T. arundinaceum* and its related species. SsAP, *S. spontaneum* AP85-441; SsNp, *S. spontaneum* Np-X; SoLA, *S. officinarum* LA-purple; SoZG, *S. officinarum* ZG1; Er, *E. rufipilus*; Ms, *Miscanthus*; Sb, *S. bicolor*; Tra, *T. arundinaceum*. Source data are provided as a Source Data file.

Additionally, we identified 4.77 Gb of repetitive sequences, which account for 70.92% of the assembled genome (Supplementary Table 10). This proportion surpasses that observed in *S. spontaneum* (AP85-441: 59.33%, Np-X: 60.61%), *S. officinarum* (LA-purple: 64.19%, ZG1: 64.77%), *E. rufipilus* (63.54%), *Miscanthus sinensis* (62.53%), and *S. bicolor* (68.36%) (Supplementary Table 11; Fig. 3e). Among these repetitive elements, long terminal repeat retrotransposons (LTR-RTs) dominated, comprising 48.19% of the *T. arundinaceum* genome, with Ty3/*Gypsy* (39.75%) being more prevalent than Ty1/*Copia* (8.34%) (Fig. 3e and Supplementary Table 10). DNA transposons accounted for 10.71% of all repetitive sequences and 7.60% of the genome (Fig. 3e and Supplementary Table 10). Analysis of the LTR-RTs insertion timing revealed two major recent burst events during the evolution of *T. arundinaceum*, occurring approximately 0.10 and 0.24 million years ago (Mya), respectively (Fig. 3d; Supplementary Fig. 9a). These successive waves of LTR-RTs proliferation likely contributed to the high abundance of transposable elements (TEs) observed in the *T. arundinaceum* genome.

Analysis of TEs distribution across chromosomes revealed that chromosome 08 (TraChr08) displayed the highest TE density, measured at 69.19%, which significantly exceeds the average density of 65.23% found on other chromosomes by 3.96 percentage points (Kruskal-Wallis test (*H* = 17.01, df = 2) with Dunn’s post-hoc test, adjusted *P* = 3.06 × 10^-04^) (Supplementary Fig. 10a; Supplementary Data 1). TraChr05 and TraChr08 are highly collinear with sorghum chromosome 5 and 8 (Fig. 2b) and correspond to paleo-duplicated chromosome pairs (PdCPs) derived from the Poaceae ρ whole-genome duplication (WGD)^20,21^. These two chromosomes exhibited significantly lower gene expression levels in *T. arundinaceum* leaves relative to other chromosomes, with mean transcript abundance reduced by approximately 40% and 15%, respectively (Kruskal-Wallis test (*H* = 2705.98, df = 2) with Dunn’s post-hoc test, adjusted *P* = 0.00 and 1.85 × 10^−98^, respectively) (Supplementary Fig. 10b). To explore the potential regulatory role of TEs on neighboring genes, we calculated the expression abundance of genes located within 2 Kb of TEs and observed a decrease in gene expression with increasing proximity to TEs (Supplementary Fig. 10c). Additionally, we found that CG and CHG methylation levels were proportionally higher on PdCPs compared to other chromosomes (Kruskal-Wallis test (CG: *H* = 19.30, df = 2; CHG: *H* = 10.31, df =2) with Dunn’s post-hoc test, adjusted *P* < 0.01) (Supplementary Fig. 10d; Supplementary Note 3). Strikingly, CG/CHG methylation levels in TEs-proximal regions correlated negatively with transcriptional activity (*P* < 2.2 × 10^-16^, Spearman’s correlation) (Supplementary Figs. 10e, 11 and 12; Supplementary Note 3). These results collectively support the hypothesis that elevated TEs methylation levels may suppress the expression of nearby genes, thereby influencing gene regulatory mechanisms within the genome.

### Evolutionary analysis of *T. arundinaceum*

To investigate the evolutionary process of the *T. arundinaceum* genome, we performed a systematic phylogenetic analysis of single-copy genes from ten representative grass species, with *O. sativa* as the outgroup (Supplementary Fig. 9b; Supplementary Note 4). The results indicated that *Saccharum* is phylogenetically closer to *S. bicolor* than to *T. arundinaceum* (Fig. 3a and Supplementary Fig. 9b). Additionally, divergence between *T. arundinaceum* and *S. bicolor* was estimated to have occurred approximately 6.67 Mya (95% confidence interval: 5.13–8.82 Mya), which aligns well with the estimate based on synonymous substitutions per site (*K*_s_) of approximately 6.54 Mya (*K*_s_ ≈ 0.085) (Supplementary Fig. 9b; Fig. 3a, b). This suggests that the origin of the diploid ancestor with a chromosome base number of *x* = 10 dates back to at least about 6.54 Mya. To estimate the timeline of polyploidization for *T. arundinaceum*, we calculated the *K*_s_ values for gene alleles. The analysis revealed that the homologous regions of TraChr05 and TraChr08 exhibited a mean *K*_s_ value of 0.022, which is approximately 38% higher than the mean *K*_s_ value (0.016) observed in homologous regions of other chromosomes (Supplementary Fig. 9e). This elevated *K*_s_ aligns with previous findings reported in *S. spontaneum*^20^. Furthermore, the divergence of the homologous chromosomes in *T. arundinaceum* occurred approximately 1.31 Mya (*K*_s_ ≈ 0.017) (Fig. 3b), indicating that the WGD leading to the autopolyploidization of *T. arundinaceum* happened earlier than that of *Saccharum*, which took place 0.77 Mya (*K*_s_ ≈ 0.010)^20^.

We also assembled the complete chloroplast genomes of *T. arundinaceum* using Illumina data. Phylogenetic analyses were conducted based on the alignments of 27 whole chloroplast genome sequences, including those from *T. arundinaceum*, *Saccharum*, and related species. Our results revealed *T. arundinaceum* clusters with *Tripidium* species (*T. ravennae*, *T. procerum*, and *T. kanashiroi*), forming a distinct clade that is separate from the Saccharinae lineage (Fig. 3c), corroborating recent phylogenomic analyses^7^. These findings further demonstrate that *T. arundinaceum* should be removed from *Erianthus* to *Tripidium*.

### Genes related to key agronomic characteristics

Biomass, photosynthesis, sugar content, and stress tolerance are key agronomic traits critical for crop improvement. To evaluate the potential of *T. arundinaceum* as a valuable germplasm resource, we sought to confirm its photosynthetic type. *T. arundinaceum* was historically placed within the C_4_ *Saccharum* complex, however, direct evidence for its C_4_ photosynthetic pathway was lacking. Anatomical observations of Kranz anatomy and C_4_-type A-Ci curves confirmed that *T. arundinaceum* performs C_4_ photosynthesis (Supplementary Fig. 14a, b; Supplementary Table 12; Supplementary Note 5).

We then performed a comparative analysis of core gene families associated with C_4_ photosynthesis, sugar accumulation, cellulose synthase, and drought response (Supplementary Figs. 13-21; Supplementary Note 5). We identified nine C_4_ pathway genes and further established that *T. arundinaceum* employs NADP-ME-type C_4_ photosynthesis (Supplementary Fig. 14c, 16 and 17; Supplementary Table 13), which is similar to that observed in sugarcane^22^. Interestingly, it lacks the canonical C_4_-type pyruvate phosphate dikinase regulatory protein (*PPDK-RP*) and instead carries a single non-C_4_-lineage *PPDK-RP* gene (*TraPPDK-RP*), which nonetheless shows increased expression along the leaf gradient (Supplementary Figs. 15 and 16i), suggesting a conserved but potentially divergent regulatory role. To further characterize the adaptive potential of *T. arundinaceum*, we analyzed the drought-induced expression patterns of six dehydration responsive element binding protein (DREB) genes (Supplementary Table 14 and 15; Supplementary Data 2), orthologs to drought-response *DREBs* in other species^23–25^. Under drought-simulating treatments with 20% (w/v) PEG 6000, *TraDREB2C* was not detectable, whereas the remaining five genes exhibited significantly altered expression compared with the control (unpaired two-tailed Welch’s *t*-test, *P* < 0.05) (Supplementary Fig. 20; Supplementary Note 5). Moreover, *TraDREB2B* displayed a distinct diurnal expression pattern under normal conditions (Supplementary Figs. 19c and 21; Supplementary Note 5), suggesting that the observed upregulation under drought treatment may not reflect a true drought-specific response but rather a confounding effect of diurnal light cycling. Taken together, these results indicate that *TraDREB2A*, *TraDREB1B*, *TraDREB1C*, and *TraDREB1J* may be involved in the drought stress response of *T. arundinaceum*. These findings provide a genetic foundation for understanding the molecular basis of stress tolerance in *T. arundinaceum* and offer candidate gene resources for future utilization.

### Population genomic study of *T. arundinaceum* accessions

**Fig. 4:**
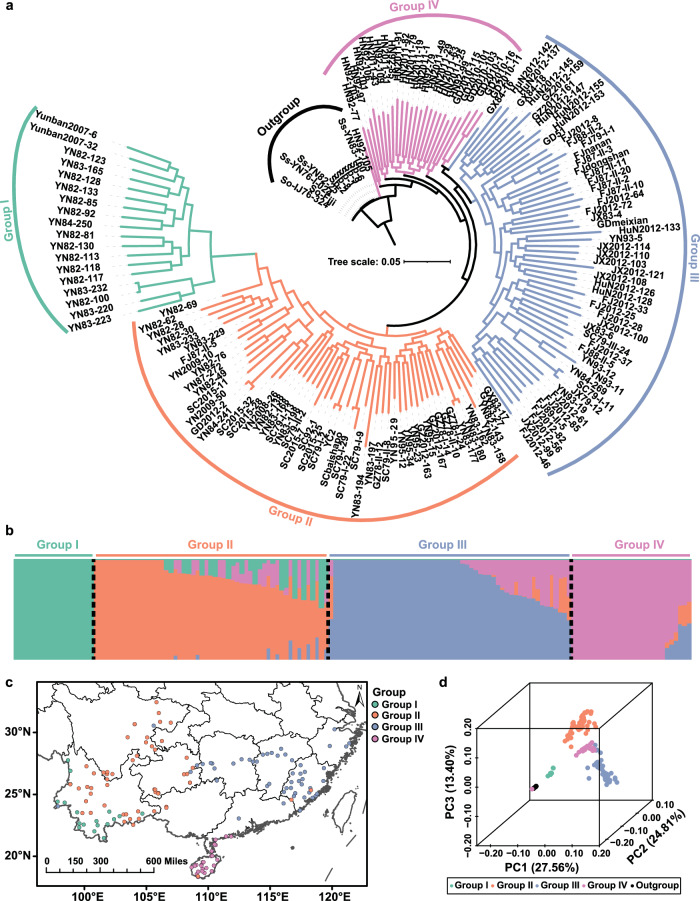
Phylogenetic relationship of 153*T. arundinaceum* accessions. **a** Maximum likelihood phylogenetic tree of *T. arundinaceum* accessions. Ss, *S. spontaneum*; So, *S. officinarum*; Sb, *S. bicolor*. **b** Population structure analysis of representative *T. arundinaceum* accessions at *K* = 4. **c** Geographic distribution of re-sequenced accessions. The base map was downloaded from the Standard Map Service System (http://bzdt.ch.mnr.gov.cn/; No. GS(2020)4619). **d** PCA plots of the first three principal components. PC1, PC2 and PC3 explain the largest proportions of variance, respectively. Colored dots indicate different subpopulations of *T. arundinaceum* accessions, and black dots represent the outgroup accessions.

To explore the genetic diversity and population structure of *T. arundinaceum*, we re-sequenced 153 *T. arundinaceum* accessions collected from 10 provinces in China, generating about 12,096 Gb of resequencing reads (Fig. 4c and Supplementary Data 3). All resequencing reads were mapped to the assembled Hainan92-105 genome for variants identification. A total of 12,844,435 high-confidence variants were identified, including 12,210,518 single nucleotide polymorphisms (SNP) and 633,917 insertions/deletions (InDels) (Supplementary Fig. 22).

**Fig. 5:**
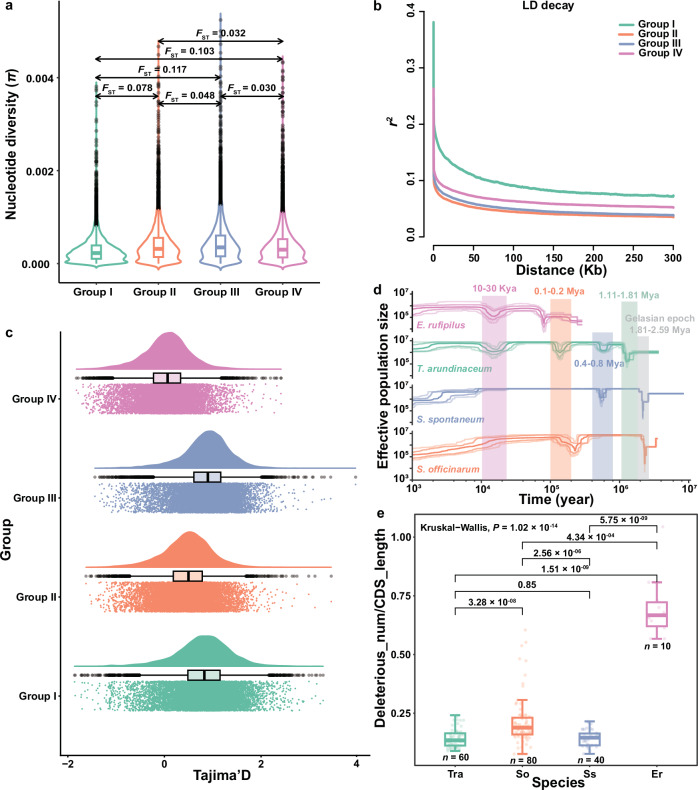
Genetic diversity of *T. arundinaceum* populations. **a** Nucleotide diversity (π) and Genetic differentiation values (*F*_ST_) among subpopulations of *T. arundinaceum*. Both π and *F*_ST_ were calculated using a non-overlapping sliding-window approach (window size = 500 kb, step size = 500 kb) across the genome. For each subpopulation, the distribution of π and *F*_ST_ values from all genomic windows are shown. The number of windows analyzed per subpopulation is denoted by *n*: Group I (*n* = 12,112), Group II (*n* = 12,223), Group III (*n* = 12,226), Group IV (*n* = 12,214). **b** Analysis of LD decay in different subpopulations of *T. arundinaceum*. LD decay distance, the physical distance at which LD (measured as *r*^2^) decayed to 0.1. **c** Tajima’D across subpopulations, calculated using the same sliding-window parameters as in **a**. *n* represents the total number of windows per subpopulation: Group I (*n* = 12,701), Group II (*n* = 12,710), Group III (*n* = 12,716), Group IV (*n* = 12,710). **d** Demographic history of *T. arundinaceum*, *E. rufipilus*, *S. spontaneum* and *S. officinarum*. **e** Deleterious mutations in chromosomes of *T. arundinaceum*, *S. officinarum*, *S. spontaneum* and *E. rufipilus*. The y-axis represents the ratio of deleterious mutation numbers to the CDS length (Kb). *n*, the chromosome number of the reference genomes for each species (*n* = 60 for Tra, 80 for So, 40 for Ss, 10 for Er). Tra, *T. arundinaceum*; So, *S. officinarum*; Ss, *S. spontaneum*; Er, *E. rufipilus*. The center line in each box represents the median; the lower and upper hinges represent the 25th and 75th percentiles, respectively; the whiskers represent 1.5× the interquartile range. Significance of pairwise comparisons was determined by Dunn’s test following a significant Kruskal–Wallis test (*H*(3) = 68.23, *P* = 1.02 × 10^-14^). *P*-values were adjusted for multiple comparisons using the Benjamini–Hochberg method. Exact *Z*-statistics, adjusted *P*-values, effect size and source data are provided as a Source Data file.

Using 20 samples from related species (5 from *S. spontaneum*, 5 from *S. officinarum* and 10 from *S. bicolor*) as an outgroup, phylogenetic analysis based on SNPs revealed that the *T. arundinaceum* population can be divided into four independent groups: Group I through Group IV (Fig. 4a). Among them, accessions in Group IV, collected from Hainan and Guangdong, may represent the earliest subpopulation due to its closest phylogenetic relationship with the outgroup. Furthermore, accessions from Yunnan province were distributed across three groups (Group I-III), indicating that these accessions possess the highest diversity (Fig. 4a, c). Principal component analysis (PCA) and genetic structure analysis further supported the classification of phylogenetic results (Fig. 4b, d; Supplementary Fig. 23a, b; Supplementary Data 4). Group II and Group III exhibited the highest levels of nucleotide diversity (π = 4.10 × 10^-4^ and 4.48 × 10^-4^, respectively) and the most rapid linkage disequilibrium (LD) decay (Fig. 5a, b). Group I showed lowest nucleotide diversity (π = 2.92 × 10^-4^) and strong genetic divergence from other three groups (pairwise *F*_ST_ ≥ 0.078; Fig. 5a), suggesting that the genetic basis of *T. arundinaceum* in Group I is relatively narrow, possibly experiencing more selective pressure. A positive value of Tajima’s D was detected in *T. arundinaceum*, indicating that the population of *T. arundinaceum* may has undergone balancing selection along with a recent population contraction (Fig. 5c and Supplementary Fig. 24).

Analysis of demographic history through the estimation of historical effective population size (*N*_e_) revealed that the two founding *Saccharum* species (*S. officinarum* and *S. spontaneum*) shared the first bottleneck event during the Gelasian stage (2.59–1.81 Mya)^26^, a period marked by a dramatic decline in temperature (Fig. 5d). In contrast, the first bottleneck event in *T. arundinaceum* population occurred slightly later, between 1.81–1.11 Mya, followed by a phase of rapid demographic expansion, corresponding to the Calabrian stage (1.80–0.77 Mya) of the Pleistocene epoch^26^. During the Naynayxungla Glaciation (0.78–0.50 Mya)^27^, both *T. arundinaceum* and *S. spontaneum* exhibited a decline in *N*_e_, likely due to their overlapping geographic distributions and shared sensitivity to climatic cooling. Subsequently, *T. arundinaceum* experienced another distinct bottleneck between 0.20-0.10 Mya, coinciding with the climatic history of Qinghai-Tibetan Plateau with the Guxiang (penultimate) Glaciation (0.30–0.13 Mya)^27^. Finally, the most resent *N*_e_ decline in *T. arundinaceum* was also observed in *E. rufipilus*, which maps to the particularly low temperatures of the Last Glacial Maximum (26.50–19.00 thousand years ago (Kya))^28^.

Furthermore, we investigated deleterious mutations in populations of *T. arundinaceum*, *S. officinarum*, *S. spontaneum*, and *E. rufipilus* by assessing SIFT (sorting intolerant from tolerant)^29^ scores based on the resequencing reads. To account for differences in genome size and gene content, we normalized the number of high-confidence deleterious alleles (SIFT score ≤ 0.05) by the total length of callable coding sequences. The normalized deleterious load in *T. arundinaceum* was similar to that in *S. spontaneum*, and both were approximately 33% lower than that in *S. officinarum* (Fig. 5e). We further assessed the functional implications of genes carrying deleterious mutations and found significant enrichment for Gene Ontology (GO) terms related to sterol metabolic process, response to cAMP, vitamin E, and nutrient (Fisher’s exact test with FDR correction, *P* < 0.01) (Supplementary Fig. 25). However, we note that direct comparisons of deleterious mutation load across species should be interpreted with caution, as each species has undergone distinct evolutionary histories and demographic processes, and differences in genome assembly, annotation and population structure may also affect our estimates.

### Identification of genomic variants associated with environmental adaptation

**Fig. 6:**
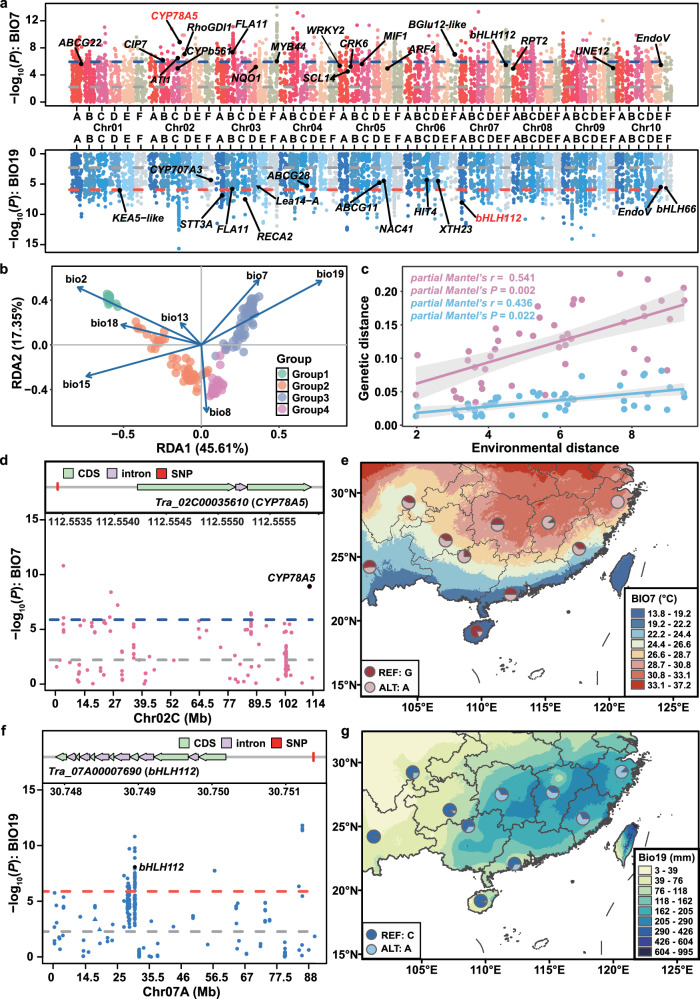
Genome-wide screening of the loci associated with local environmental adaptation. **a** Manhattan plot for variants associated with annual temperature range (BIO7) (upper panel) and precipitation of the coldest quarter (BIO19) (lower panel). SNPs and Indels are represented by points and triangles, respectively. Dashed horizontal lines represent significance thresholds: gray for the Benjamini–Hochberg adjusted FDR threshold (FDR = 0.01); blue (BIO7) and red (BIO19) for the Bonferroni-correction threshold (corrected *P* = 0.01). Selected candidate genes are labeled in the plot at their respective genomic positions. **b** *T. arundinaceum* accessions mapped on the first two canonical axes of RDA. Arrows represent seven environmental factors correlated with the genotypes of *T. arundinaceum*. Colored points represent accessions from different subpopulations. **c** Isolation-by-environment analyses (partial Mantel test, two sided, controlling for the effect of geographic distance) for populations (*n* = 45) based on adaptive (purple dots and line) and neutral variants (blue dots and line), separately. The shadow of linear regression denotes the 95% confidence interval. **d**, **f** Upper panels: the gene structure of *CYP78A5* (**d**) and *bHLH112* (**f**), red lines: candidate adaptive variants corresponding to the sites shown in **e** and **g**; Lower panels: local magnification of the Manhattan plots around the selected genes. Dashed horizontal lines follow the same significance thresholds as described in **a**. **e**, **g** Allele frequencies of candidate adaptive variants Chr02C_112553480 (**e**) and Chr07A_30751379 (**g**). Colors on the map are based on variations of the relevant climate variables across the distribution range. The base map was downloaded from the Standard Map Service System (http://bzdt.ch.mnr.gov.cn/; No. GS(2020)4619). In all Manhattan plots, significance of locus–environment associations was assessed using latent factor mixed models (LFMM), with *P*-values derived from z-scores via a chi-square test (df = 1). Test statistics were adjusted by dividing by the genomic inflation factor (λ). *P*-values were corrected for multiple testing using the Benjamini–Hochberg procedure (FDR = 0.01). Source data are provided as a Source Data file.

Local adaptation has the potential to generate correlations between genomic loci and environmental variables. To identify variants associated with environmental conditions in *T. arundinaceum*, we employed two complementary genotype-environment association (GEA) methods in this study. First, we applied redundancy analysis (RDA)^30^ to detect variants co-varying with multivariate environmental predictors, which initially included ten temperature-related and nine precipitation-related variables (Supplementary Table 16). After addressing multicollinearity among these environmental variables, we retained seven variables with variance inflation factors (VIF) less than 10 for the RDA analyses, including three temperature variables: mean diurnal range (BIO2), temperature annual range (BIO7), and mean temperature of coldest month (BIO8); and four precipitation variables: precipitation of wettest month (BIO13), precipitation seasonality (BIO15), precipitation of warmest quarter (BIO18), and precipitation of coldest quarter (BIO19) (Supplementary Data 5). This RDA analysis identified 121,365 candidate variants with extreme loadings (standard deviation > 3) along one or multiple RDA axes (Fig. 6b).

Additionally, we conducted a latent factor mixed model (LFMM)^31^ analysis, which is a univariate approach that assesses the association between individual genetic variants and specific environmental variables. This analysis identified 188,855 variants significantly associated with one or more environmental variables (adjusted *P* < 0.01, false discovery rate (FDR) correction). 7592 of these variants (comprising 7532 SNPs and 60 Indels) overlapped with the candidate variants identified by RDA. We considered these overlapping variants as “core adaptive variants” relevant to local climate adaptation, which exhibited widespread distribution across the genome (Supplementary Data 6; Supplementary Fig. 26).

To evaluate the potential adaptive variants identified in this study, we first employed Mantel and partial Mantel tests to examine patterns of isolation-by-distance (IBD) and isolation-by-environment (IBE) for both adaptive and neutral variants. The results showed that both adaptive and neutral variants displayed significant patterns of IBD and IBE among populations of *T. arundinaceum* (Fig. 6c; Supplementary Table 17). Importantly, adaptive variants exhibited a stronger IBE than neutral variants after controlling for geographical effects in the partial Mantel tests (Fig. 6c; Supplementary Table 17). Moreover, we performed partial RDA to assess the relative contributions of climatic and geographical factors to adaptive and neutral genetic variation. Our analysis revealed that environmental and geographic variables explained 24.85% and 17.32% of the genetic variation among adaptive variants, respectively (*P* = 0.001) (Supplementary Table 18). When controlling for the confounding effects of geographic variables, climatic factors independently explained 8.51% of the genetic variation in adaptive variants. This contribution was 2.62-fold greater than that of neutral variants, which accounted for 2.35%, and 7.60-fold higher than the exclusive influence of geographic variables, which explained only 0.99% of the variation in adaptive variants (Supplementary Table 18). These results suggest that the adaptive variants identified in this study were predominantly shaped by environmental factors.

The core adaptive variants were annotated to 836 genes (Supplementary Data 6) associated with environmental variables, primarily involved in processes such as FMN reductase (NADPH) activity, oxidoreductase activity, cytosolic transport, actin filament polymerization/organization, actin polymerization or depolymerization, and cytoskeletal motor activity (Supplementary Fig. 27, Supplementary Data 7). Among these genes, *CYP78A5*, a cytochrome P450s protein, has been reported to confer osmotolerance and tolerance to osmo-shock, salt-shock, oxidative, heat stress, and drought when overexpressed^32,33^. In our study, two candidate adaptive SNPs showed a relatively high degree of LD which located in the upstream region (within 2 Kb) of *CYP78A5* (*Tra_02C00035610*) were significantly (adjusted *P* ≤ 1.45 × 10^−09^) associated with BIO7 (Temperature Annual Range) (Fig. 6a, d; Supplementary Fig. 28a; Supplementary Data 6). Chr02C_112553480 was chose an example for allele frequency analysis, found that this candidate adaptive SNP showed a significantly allele frequency distribution (*P* = 0.008, two-tailed Student’s *t*-test) (Supplementary Fig. 28c). The A allele was predominantly found in regions with a high temperature annual range (24.4–33.1 °C), while the G allele was nearly fixed in areas with low temperature annual range (13.8–24.4 °C) (Fig. 6e). Additionally, several other genes were identified as contributors to temperature-associated adaptation (Supplementary Figs. 29 and 30; Supplementary Data 8). For example, the *MYB30* transcription factor regulates heat stress response through ANNEXIN-mediated calcium signaling^34^; overexpression of *WRKY2* improves transgenic *Arabidopsis* tolerance to salt and drought by activating the STZ pathway^35^; and *BGlu12*, a β-glucosidase, was found highly induced in response to UV-B radiation and dehydration^36^. The variants associated with these genes also showed allele frequency distributions that correlated with temperature (Supplementary Figs. 29 and 30).

We also identified several precipitation-related genes that potentially respond to environmental stress, including *bHLH112*^37,38^, *bHLH66*^39^, *Lea14-A*^40^, *CYP707A3*^41^, *ABCG11*^42^, *CRLK1*^43^, *bZIP60-like*^44^, *HIT4*^45^, and *EF1α*^46^ (Supplementary Figs. 31–33, Supplementary Data 8). These genes exhibited allele frequency distribution patterns that significantly correlate with precipitation in our analysis (*P* < 0.05, two-tailed Student’s *t*-test) (Fig. 6g; Supplementary Fig. 28d and 31-33). For example, *bHLH112* (*Tra_07A00007690*), a basic helix-loop-helix transcription factor, emerged as a key gene associated with candidate adaptive variants related to the precipitation of the coldest quarter (BIO19) (Fig. 6a, f). This finding is particularly relevant given the established role of *bHLH112* ortholog in drought and salt responses in *Arabidopsis*^37^. Further investigation revealed a relatively high level of LD in the genomic region surrounding *bHLH112*, which contains four candidate adaptive SNPs (Supplementary Fig. 28b). Among them, one SNP, located in the promoter region of *bHLH112*, was selected to illustrate the geographic distribution of the allele frequencies. Our allele frequency analysis revealed that populations in areas with high precipitation (162–290 mm) during the coldest quarter predominantly carried the A allele, while the C allele was more common in areas with low rainfall (39–162 mm) (Fig. 6g). These findings suggest that the genes associated with the core adaptive SNPs may play important roles in environmental adaptation among natural populations of *T. arundinaceum*.

## Discussion

Understanding the phylogenetic relationships among members of the Andropogoneae is essential for both evolutionary biology and crop improvement. *T. arundinaceum* has traditionally attracted considerable research interest due to its excellent biological traits and ecological dominance in southern China^9–13^; however, the phylogenetic relationship of *T. arundinaceum* has been debated. Molecular phylogenetic analysis based on ITS sequences indicated a close genetic relationship between *T. arundinaceum* and *Erianthus*^13^, while random amplified polymorphic DNA (RAPD), amplified fragment length polymorphism (AFLP), and microsatellites suggested that *T. arundinaceum* should be classified as *Erianthus* or placed in a new genus^47,48^. Chloroplast protein-coding genes analysis has shown that *S. officinarum* is more closely related to *M. sinensis* than to *T. arundinaceum*, with the latter diverging from the Sorghinae before the divergence of *S. bicolor* from the common ancestor of *S. officinarum* and *M. sinensis*^49^. In our study, phylogenetic analysis based on the whole genomes of ten grass species suggests that *Saccharum* is more closely related to *S. bicolor* than to *T. arundinaceum*, which is in line with our findings from the chloroplast phylogenetic tree (Fig. 3c and Supplementary Fig. 9b). Importantly, the chloroplast genome phylogeny further supports the reclassification proposed by Lloyd Evans et al.^7^ and Welker et al.^8^, placing of *T. arundinaceum* within the genus *Tripidium*, distinct from both *Saccharum* and *Erianthus*. This substantial evolutionary distance between *Saccharum* and *T. arundinaceum* may explains the long-standing difficulties in utilizing *T. arundinaceum* for sugarcane improvement, as genetic recombination between such distantly related species is inherently difficult.

Divergence time estimates provide additional insight into the evolutionary history of these lineages. We estimated that *S. bicolor* and *Saccharum* diverged roughly 6.38 Mya (Fig. 3b), a finding that aligns well with previous estimates of their divergence time^20^. Moreover, *T. arundinaceum* diverged from *S. bicolor* approximately 6.54 Mya, indicating that *T. arundinaceum* separated from *S. bicolor* prior to the divergence of *S. bicolor* and *Saccharum*, consistent with the findings of Tsuruta et al.^49^. In contrast, *E. rufipilus* and *Saccharum* diverged from a common ancestor approximately 1.9–2.5 Mya^50^. Therefore, it is proposed that *T. arundinaceum* underwent independent polyploidization from a diploid ancestor distinct from *Saccharum*, estimated to have occurred approximately 1.31 Mya (Fig. 3a). Most *Tripidium* species are polyploid, and, given their relatively close phylogenetic relationship to sugarcane alongside evidence for independent polyploidization events, *Tripidium* represents one of the closest polyploid lineages to *Saccharum*, providing a valuable comparative framework for understanding polyploid evolution and genome diversification in sugarcane.

TEs are important components of eukaryotic genomes that generate extensive variations in gene expression and function in plants, contributing substantially to their adaptive evolution^51,52^. In the case of *T. arundinaceum*, the genome experienced two distinct bursts of LTR-RT activity following polyploidization, which likely accounts for its elevated TE content compared to other members of the Andropogoneae. These two major LTR-RT burst events coincide with a demographic bottleneck in *N*_e_ of *T. arundinaceum*, which temporally overlaps with the dramatic temperature decline in the Guxiang Glaciation (0.3–0.13 Mya) (Figs. 3d and 5d). These findings suggest that *T. arundinaceum* may have adapted to harsh environments through these burst events of LTR-RTs during its evolutionary history. Furthermore, the monoploid genome size of *T. arundinaceum* substantially exceeds that of *Saccharum*, indicating that these TE bursts may have contributed significantly to its genomic expansion. Collectively, our results underscore the important of TEs in shaping the structure and evolution of the *T. arundinaceum* genome and suggest their potential contribution to environmental adaptation.

Assessment of genetic diversity in *T. arundinaceum* employing various molecular markers revealed a high level of genetic variation across its distribution range, which is critical for advancing introgression breeding and germplasm conservation^53–57^. Phylogenetic analysis, coupled with assessment of current geographical distribution, indicates that *T. arundinaceum* accessions from China which clustered together share a similar genetic background. Three distinct subpopulations (Groups I–III) were identified in Yunnan Province. Groups I and II consisted predominantly of accessions collected from Yunnan (Fig. 4a, c), with Group II exhibiting the highest nucleotide diversity and the fastest LD decay (Fig. 5a, b), indicating pronounced intra-specific diversity within southwestern China (Yunnan). These observations corroborate previous findings by Cai et al.^54^, supporting the hypothesis that southwestern China (Yunnan) may serve as a center of genetic diversity for *T. arundinaceum* within China. Comparative demographic analysis revealed parallel declines in *N*_e_ between *T. arundinaceum* and *S. spontaneum* (Fig. 5d), implying potential shared biogeographic origins. According to Zhang et al.^20^, *S. spontaneum* is believed to have originated in northern India; thus, it is likely that *T. arundinaceum* also traces its origins to this region, given its similar distribution observed across northern India (Supplementary Fig. 23c). However, definitive confirmation would require extensive phylogeographic analysis with dense sampling across the entire distribution range of *T. arundinaceum*, particularly in northern India.

*T. arundinaceum* exhibits a broad distribution across tropical and subtropical regions, with particularly high abundance observed in the eastern Tibetan Plateau and areas south of the Huai River-Qinling Mountains line in China^13^. This wide geographic range reflects its exceptional adaptability to a variety of environments, including drought, cold, and saline soils^13^. Identifying genetic variations associated with climate is essential for elucidating fine-scale patterns of local adaptation and enhancing our understanding of species responses to climate change^58^. Our GEA analysis identified multiple candidate loci linked to temperature and precipitation across the distribution range of *T. arundinaceum*. Some genes associated with these loci have well-established roles in abiotic stress responses in plants. For example, genes such as *bHLH112*^38^, *bHLH66*^39^, *Lea14-A*^40^, *CYP94C1*^59^, *MYB44*^60^, *ABCG22*^61^, *WRKY2*^35^, and *CYP707A3*^41^ have been shown to play critical roles in responses to drought stress, while *CYP78A5*^32^, *MYB30*^34^, *EF1α*^46^, *CRLK1*^43^, *bZIP60-like*^44^, *ABCG11*^42^, and *HIT4*^45^ have been implicated in the regulation of heat or cold stress (Supplementary Data 8). These findings underscore the complexity of local adaptation in *T. arundinaceum*, indicating that multiple genetic pathways contribute to its resilience against environmental stresses. Overall, our results provide a comprehensive genomic perspective on the evolutionary genetics and ecological adaptations of *T. arundinaceum*, laying a foundation for future functional studies and the potential utilization of its stress-tolerance resources.

## Methods

### Plant materials

The *T. arundinaceum* accession Hainan92-105, selected for genome assembly and annotation, was cultivated at Fujian Agriculture and Forestry University (Fuzhou, Fujian, 119°16′48″ E, 26°4′48″ N). For population genomic analysis, additional *T. arundinaceum* accessions, originally sourced from various regions and altitudes, were planted at the National Germplasm Repository of Sugarcane in Kaiyuan, China. Detailed information about them is provided in Supplementary Data 3.

*T. arundinaceum* Hainan92-105 used for transcriptome sequencing were cultivated at Fujian Agriculture and Forestry University. Leaf, stem, and root tissues were collected from 45-day-old seedling grown in a climate-controlled chamber (light intensity at 350 μmol m⁻² s⁻¹, 60% relative humidity, with a temperature of 30 °C under continuous light for 14 h, followed by 10 h of darkness at 22 °C), while flowers harvested from plants at the booting, heading, and mature stages in the field. For the leaf gradient experiment, samples were taken from the second leaves of approximately 9-day-old *T. arundinaceum* Hainan92-105 plants grown under the same chamber conditions. These leaves were harvested 3 h into the light period in the morning and cut into seven 1-cm segments^22^. Additionally, for the diurnal cycle experiment, mature leaves (12-month-old) were sampled at 2 h intervals over a 24 h period (6:00 a.m., 8:00 a.m., 10:00 a.m., noon, 2:00 p.m., 4:00 p.m., 6:00 p.m., 8:00 p.m., 10:00 p.m., midnight, 2:00 a.m., 4:00 a.m., and 6:00 a.m.) on November 6–7, 2019, following established methods^62^. For each biological replicate, tissues were pooled from three individual plants, with three independent biological replicates analyzed in total.

### Analysis of chromosome number in *T. arundinaceum*

Fresh and tender root tips of *T. arundinaceum* Hainan92-105 were harvested and pretreated with nitrous oxide (N_2_O) for 1.5 h. The root tips were fixed in a 3:1 (v/v) solution of ethanol:acetic acid and stored at −20 °C until further use. Following washing with distilled water, the root tips were incubated in an enzyme mixture consisting of 4% cellulase (Yakult Pharmaceutical, Tokyo, Japan), 2% pectinase (Sigma-Aldrich), and 2% pectolyase (Plant Media, Shawnee Mission, KS, USA) at 37 °C for 4–5 h. After removing the enzyme solution, the root tips were washed with distilled water, and then broken by using a pipette tip to separate cells from one another. Afterward, the root tips were fixed in fixative solution (ethanol:acetic acid, 3:1). The cell suspension was dripped onto a glass slide and air-dried, after which the slides were stored at −20 °C until needed for analysis.

The FISH experiments were performed following the published protocols^63^. The 20 μL of hybridization solution containing 2 μL of biotin-labeled probes and 2 μL of digoxigenin-labeled probes was hybridized with the target slide at 37 °C overnight. After slowly removing the coverslips, the slides were washed in 2× SSC and 1× PBS solution for 5 min, respectively. Chromosomes were counterstained with 4,6-diamidino-2-phenylindole in VectaShield antifade solution (Vector Laboratories). Metaphase plates showing fluorescent signals were observed and photographed using an AxioScope A1 fluorescent microscope. Images were subsequently processed using Adobe Photoshop software and Image J software.

### Genome sequencing

For PacBio sequencing, fresh young leaves were collected from individual *T. arundinaceum* Hainan92-105 plants and send to Biomarker Technologies (older sequencing) and Berry Genomics (newly sequencing) for the preparation of libraries and subsequent sequencing. For Illumina sequencing, paired-end 150 bp reads were generated on the Illumina HiSeq and DIPSEQ platforms provided by Novergene and BGI, respectively. For HiC-library construction, fresh and tender leaves were submitted to Biomarker Technologies (Beijing, China) and used to prepare chromatin crosslinked to DNA and fixed with formaldehyde. Subsequently, the crosslinked DNA was digested using the HindIII restriction enzyme, producing sticky ends that were biotinylated and proximity-ligated to form chimeric junctions. The processed DNA was further enriched and physically sheared into fragments of 300–700 base pairs (bp). Finally, four Hi-C libraries were sequenced on the Illumina platform. For RNA-sequencing (RNA-seq), total RNA was extracted from the root, stem, leaf, and flower bud tissues of *T. arundinaceum* Hainan92-105 using TRIzol (Invitrogen). These RNA samples were subsequently sent to Novogene (Beijing, China) for the construction of transcriptome libraries, which were sequenced on the Illumina platform.

### Genome survey

About 433.85 Gb of Illumina short reads were filtered using Trimmomatic^64^ (v0.39) software with default parameters. The clean reads were then processed to create *k*-mer sets using Jellyfish^65^ (v2.2.6) to calculate the genome size and heterozygosity, employing a *k*-mer value of 21.

### Haplotype-resolved genome assembly

Minor discrepancies between HiFi reads generated from the two separate sequencing runs could potentially affect the assembly software’s assessment of homozygous read coverage, which may lead to an overestimation of the genome size. To address this issue, we performed de novo assembly using only the newly generated HiFi reads, while leveraging the older HiFi dataset solely to enhance assembly contiguity. We adopted the hybrid assembly mode of hifiasm (Ultra-long ONT integration), an approach that effectively works with polyploid genomes^66^. Hifiasm (v0.19.6-r595) first constructs a string graph using the newly generated 238 Gb HiFi reads (~36× coverage). We then extracted reads longer than 20 Kb (47 Gb, ~7× coverage) from the older HiFi dataset and mapped them onto the graph to generate the final assembly. This dataset was used in place of ultra-long ONT reads to improve assembly contiguity, resulting in a 2.3 Mb increase in the initial unitig N50 of *T. arundinaceum*. By aligning the unitigs to the NCBI bacterial database, we filtered them accordingly. HapHiC^67^, an allele-aware scaffolding tool based on Hi-C data (~95× coverage), was employed to cluster the unitigs into 60 groups, representing the 60 chromosomes of *T. arundinaceum*. The unitig graph captured the connectivity between unitigs, which we used to further link unitigs belonging to the same chromosome, yielding the final haplotype-resolved genome.

### Genome quality assessment

To evaluate the accuracy of genome assembly, Illumina and HiFi reads were mapped to the assembly using BWA (v0.7.17)^68^ and minimap2 (v2.26)^69^, respectively. Subsequently, PanDepth^70^ and mosdepth (v0.3.5)^71^ were employed to calculate reads depth. Reference free QV estimation was performed using Merqury (v1.3)^72^. Additionally, BUSCO^73^ and compleasm^74^ analysis were conducted to assess the presence of Embryophyta genes in the final assembly.

### Comparative genome analysis and divergence time assessment

We performed a whole-genome synteny analysis among *T. arundinaceum* Hainan92-105, *S. bicolor* (https://phytozome.jgi.doe.gov/info/Sbicolor_v5_1), *E. rufipilus*^50^, and *Saccharum* (*S. spontaneum*: Np-X^20^ and AP85-441^19^; *S. officinarum*: ZG1 and LA-purple (https://sugarcane.gxu.edu.cn/scdb/download)) using MCScanX^75^ and JCVI^76^ with default parameters. To estimate evolutionary divergence time, we calculated synonymous substitution rates (*K*_s_) values for orthologous gene pairs identified from synteny blocks, using the Nei-Gojobori method as implemented in the pipeline (https://github.com/tanghaibao/bio-pipeline/tree/main/synonymous_calculation). Species divergence time (*T*) were then derived from peak *K*_s_ values according to the formula:

$$T=K_s/(2\times 6.5\times {10}^{-9})$$

where 6.5 × 10^−9^ represents the grass-specific substitution rate per site per year^77^.

### Repeat prediction

Repetitive sequences were annotated using RepeatModeler (open-1.0.8) (https://github.com/Dfam-consortium/RepeatModeler) to generate consensus TE sequences, followed by RepeatMasker (open-4.0.5)^78^ for classification and masking of repetitive elements. For a detailed investigation of LTR-RTs, highly confident intact LTR-RTs were identified using the LTR_retriever^79^ pipeline. This pipeline integrates the results from LTR_FINDER^80^ and LTRharvest^81^, and efficiently removes false positives from the initial predictions.

### Gene model prediction and functional annotations

Gene model prediction was performed using the BRAKER3^82^ pipeline (https://github.com/Gaius-Augustus/BRAKER), incorporating RNA-seq data from root, stem, and leaf tissues, as well as protein sequences from *O. sativa* (https://phytozome-next.jgi.doe.gov/info/Osativa_v7_0), *S. bicolor* (https://phytozome-next.jgi.doe.gov/info/Sbicolor_v3_1_1), and *E. rufipilus*^50^. High-confidence gene models were generated through training and prediction with GeneMark-ETP^83^ and AUGUSTUS^84^. UTR regions were predicted from RNA-seq data using the script stringtie2utr.py of BRAKER3. AlleleFinder pipeline (https://github.com/sc-zhang/AlleleFinder) was used to identify allele genes in the *T. arundinaceum* genome with the parameters ‘-n 6 -m -b 2 --blast_identity 80 --ovlp_ratio 0.8’.

Gene functions annotation for the *T. arundinaceum* Hainan92-105 genome were performed through multiple complementary approaches. First, protein sequences were aligned against the SWISS-PROT, NR (National Center for Biotechnology Information (NCBI) non-redundant), and Uniport databases using BLASTP (v2.10.0 + ) (*E*-value < 1×10^−5^). Subsequently, EggNOG-Mapper^85^ was employed for the eggNOG annotation, and Gene Ontology (GO) functional annotation and enrichment analysis were carried out. Finally, to assess whether the proteins encoded by these genes might participate in specific functional pathways, we utilized KofamKOALA^86^ software.

### Whole genome methylation analysis

Fresh leaves of *T. arundinaceum* Hainan92-105 were collected and sent to Novogene (Beijing, China) for Oxford Nanopore Technology (ONT) library construction using the genomic sequencing kit SQK-LSK109 (Oxford Nanopore Technologies, Oxford, UK). The libraries were subsequently sequenced on the PromethION platform. To identify methylation in three contexts (CG, CHG, and CHH), we utilized DeepSignal-plant (v.0.1.6)^87^, which uses a deep-learning method for detecting methylation. The raw fast5 files were base-called using Guppy^88^ and then re-squiggled with tombo^89^. Methylation of specified motifs was called by a 5mC model (model.dp2.CNN.arabnrice2-1_120m_R9.4plus_tem.bn13_sn16.both_bilstm.epoch6.ckpt), which was trained using R9.41D reads of *A. thaliana* and *O. sativa*. The script call_modification_frequency.py, provided in the DeepSignal-plant package, was then used to generate the methylation frequency at each CG, CHG and CHH site. Only sites covered by more than three mapped reads were included in subsequent analyses. To calculate TE methylation, the body region and upstream or downstream 2 kb regions were proportionally divided into 30 bins and 100 bins, respectively. Then, the average DNA methylation level of each bin was calculated for all TEs and plotted.

### Identification of key genes

To identify genes associated with key agronomic traits in *T. arundinaceum*, we performed comparative analyses using three reference species (*O. sativa*, *Z. mays*, and *S. bicolor*). Protein sequences of candidate genes from these reference species were retrieved from phytozome v13 (https://phytozome-next.jgi.doe.gov/) and NCBI. Homologous genes in *T. arundinaceum* were identified through BLASTP analysis (*E-*value < 1 × 10 ^-5^). Gene counts related to sugar pathway, photosynthetic, and cellulose synthase for *O. sativa*, *Z. mays*, *S. bicolor*, and *Saccharum* were sourced from Zhang et al.^20^, while data of *DREB* genes were collected from previous studies^23–25,90,91^.

### Expression profile analysis

RNA-seq reads from multiple tissues (leaves, stems, roots and flowers) were quality-trimmed using Trimmomatic^64^ and subsequently mapped to allele-aware annotated gene models utilizing Trinity^92^. Transcript abundance was quantified as transcripts per million (TPM) using the RSEM^93^ program, which is implemented in the Trinity^92^ package.

To analyze the expression patterns of the *TraDREB* genes under drought stress, we performed a short-term time-course experiment. Two-month-old *T. arundinaceum* Hainan92-105 seedlings were transplanted into Hoagland solution for hydroponic cultivation under controlled environment conditions (30 °C, 60% relative humidity, 14 h photoperiod). After one week adaptation, the nutrient solution was removed, and the seedlings were placed on a clean bench (30 °C, 60% relative humidity) to simulate drought stress. Leaf samples were collected at 0, 3, 6, and 9 h post-treatment (3 plants per biological replicate, 3 replicates). The entire sampling process was conducted within a 9 h period on the same day to minimize the influence of circadian rhythms on gene expression, with the 0 h sample serving as the control for calculating relative expression changes at subsequent time points. Total RNA was isolated from leaves using the RNeasy Plant Mini Kit (Qiagen, Cat. No. 74904) and used for the real-time quantitative reverse transcription-PCR (RT-qPCR) analysis with the expression levels of *TraeEF-1a* (Eukaryotic elongation factor 1-alpha) and *TraHIP1* (E3 ubiquitin-protein ligase) as internal controls. cDNA synthesis was performed with StarScript II RT Mix with gDNA Remover (GenStar, A224-10), following the manufacturer’s protocol. RT-qPCR amplification was conducted using the 2× RealStar Fast SYBR qPCR Mix (GenStar, A301-10) on a Bio-Rad Multicolor Real-Time PCR Detection System, with a two-step thermocycling program as specified in the kit’s protocol. Gene-specific primers were designed using PrimerQuest (Integrated DNA Technologies: http://www.idtdna.com/Primerquest/Home/Index) (Supplementary Table 19). The expression patterns of *TraDREBs* measured by RT-qPCR were further supported by an independent transcriptome data from one-month-old *T. arundinaceum* seedlings subjected to drought stress with PEG 6000 (20%, w/v) and sampled at 0 h, 4 h, 8 h, 16 h, or 32 h after treatment.

### Phylogenetic analysis of *T. arundinaceum* and relative species

Single-copy orthologs were identified from the genomes of *T. arundinaceum*, *S. spontaneum* (AP85-441 and Np-X), *S. officinarum* (LA-purple and ZG1), *E. rufipilus*, *M. sinensis*, *S. bicolor*, *Z. mays*, and *O. sativa* through cluster analysis of gene families using Orthofinder^94^. Single-copy genes were clustered with MUSCLE^95^ to construct a super alignment matrix, which was used to construct a phylogenetic tree with RAxML^96^ based on the maximum likelihood method with 1000 replications. Divergence times were estimated using MCMCTREE from the PAML^97^ package, and the expansion and contraction of gene families were assessed using Computational Analysis of Gene Family Evolution (CAFÉ5)^98^.

Additionally, organelle-associated reads were extracted from the Illumina short reads, and the complete chloroplast genomes of *T. arundinaceum* were de novo assembled using GetOrganelle (v1.7.7.1)^99^. The assembled chloroplast genome sequences, together with those of related species retrieved from NCBI GenBank (https://www.ncbi.nlm.nih.gov/nuccore), were auto-aligned using MAFFT (v7.505)^100^. The best-fit evolutionary model, TVM + I + G, was selected using JModelTest2^101^ to conduct maximum likelihood phylogenetic analysis with RAxML-NG^102^.

### Population genomics

The raw paired-end Illumina reads of 153 *T. arundinaceum* accessions were processed using Trimmomatic^64^ to remove adaptors and low-quality bases. Clean reads were then aligned to the Hainan92-105 genome using BWA-MEM with default parameters^103^. Genomic variant call format (GVCF) files for each accession were generated and subsequently combined using the Sentieon pipeline^103^. First, duplicated reads in the resulting BAM files were removed with the command ‘sentieon driver --algo Dedup’. Followed by base recalibration using the ‘sentieon driver --algo QualCal’ command. Variants were then called separately for each accession with the command ‘sentieon driver --algo Haplotyper’. Afterward, the GVCF files of all *T. arundinaceum* individuals were merged using the ‘sentieon driver --algo GVCFtyper’ command. The raw variant set was subsequently filtered using VCFtools^104^ with the following parameters: ‘--max-missing 0.8 --maf 0.05 --max-alleles 2 --min-meanDP 4 --minQ 200’. The SnpEff v4.3t^105^ program was employed to annotate the variants based on gene models from Hainan92-105 genome annotation, using the parameter ‘-ud 2000’ to define the length of upstream and downstream regions around genes. The variant sites were annotated as the SNP and Indels, as well as intergenic and genic regions. Additionally, SIFT4G^29^ was performed for nonsynonymous and synonymous annotations.

### Phylogenetic and population analysis

A maximum likelihood tree was constructed using RaxML (v8.12)^96^ with 2000,000 random SNPs, and the format conversion of the input file was performed using vcf2phylip (v2.0) (https://github.com/edgardomortiz/vcf2phylip). Finally, iTOL^106^ was utilized to visualize the tree. Principal component analysis (PCA) was analyzed by PLINK (v1.90)^107^ software. Population structure analysis was performed using ADMIXTURE (v1.3.0)^108^ for *K* values ranging from 1 to 10, utilizing a linkage disequilibrium (LD) pruned SNP set extracted by PLINK (v1.90)^107^ with the parameters ‘indep-pairwise 50 10 0.2’. Based on the consistency of the result at *K* = 4 with the phylogenetic tree topology and PCA, we selected *K* = 4 as the optimal value for our analysis. Values of Nucleotide diversity (π), Tajima’s D, and genetic differentiation values (*F*_ST_) were calculated by VCFtools^104^. LD decay was assessed using PopLDdecay (v 3.31)^109^ with default parameters, analyzing all pairs of SNPs within 300 Kb.

### Demographic analysis

To calculate the site-frequency spectrum (SFS) of *T. arundinaceum*, we employed ANGSD^110^ to compute the site allele frequency likelihood based on individual genotype likelihoods assuming Hardy–Weinberg Equilibrium (HWE) from filtered BAM files with the parameter ‘-doSaf’. Then the ‘-realSFS’ parameter was used to derive a maximum likelihood estimate of the SFS by the Expectation Maximization (EM) algorithm. The folded SFS was subsequently applied to estimate the population demographic history by Stairway plots^111^. For this analysis, we used a mutation rate of 6.5 × 10^−9^ and a generation time of 1 year.

### Deleterious mutations

Clean reads from *T. arundinaceum*, *S. spontaneum*, *S. officinarum*, and *E. rufipilus* accessions were aligned to their respective reference genomes (*T. arundinaceum*: Hainan92-105, *S. spontaneum*: Np-X^20^, *S. officinarum*: ZG1, *E. rufipilus*: Yunnan2009-3^50^) using BWA-MEM with default parameters^103^. Variants were then identified using Sentieon pipeline^103^. UniRef90 https://www.uniprot.org/uniref?query = (identity:0.9) was used as the reference protein database to build the SIFT4G database of *T. arundinaceum*, *Saccharum*, and *E. rufipilus*. Furthermore, we employed SIFT4G annotator^29^ to annotate SNP data. SIFT utilizes protein alignments to identify conserved amino acids and provides a score (0-1) reflecting the potential deleterious impact of each position in the protein. Positions with a SIFT score of less than 0.05 were predicted as “DELETERIOS” or “DELETERIOS (Low confidence)”, while positions with a score ≥ 0.05 were classified as “TOLERATED”. In this study, we focused on “DELETERIOS” SNPs.

### Ecological niche modeling

Ecological niche modeling was performed using Maxent (v3.4.4)^112^ to predict the current potential distribution of *T. arundinaceum*. A total of 279 occurrence records were compiled, comprising 153 samples collected in this study (Supplementary Data 3) and an additional 126 records retrieved from the Global Biodiversity Information Facility (GBIF; https://www.gbif.org/) and the Chinese Virtual Herbarium (CVH; http://www.cvh.ac.cn/). Duplicate records were removed prior to analysis. Nineteen bioclimatic variables (BIO1-19) for the period 1970-2000 (current) were extracted from WorldClim 2.1 (https://www.worldclim.org/) at a spatial resolution of 2.5 arc-minutes. To reduce multicollinearity, pairwise Spearman’s rank correlation coefficients were calculated among all variables, and those with |*r* | > 0.7 were excluded. The final model was constructed using the remaining uncorrelated variables with default parameters and 10 replicated runs, with 25% of the occurrence records randomly withheld for model evaluation.

### Identification of environment associated genetic SNPs

Following the approach outlined by Sang et al.^113^, we employed two complementary methods to identify environment-associated variants across the genome, utilizing the common variants with a minor allele frequency (MAF) > 10%. First, we conducted a redundancy analysis (RDA) to identify genetic variants that exhibit a strong correlation with multivariate environmental axes using the R package vegan (v2.7.1)^30,114,115^. To exclude multicollinearity among these 19 bioclimatic variables, we computed variance inflation factors (VIFs) using the ‘vif’ function from the R package usdm (v2.1.7)^116^, removing variables with a VIF greater than 10. Consequently, seven environmental variables (BIO2, BIO7, BIO8, BIO13, BIO15, BIO18, and BIO19) were retained for further analyses. Significant environment-associated variants were identified as those displaying loading in the tails of the distribution, using a standard deviation cutoff of 3 (two-tailed *P* = 0.0027) along one or more RDA axes. Second, we employed a univariate latent-factor linear mixed model (LFMM) implemented in the R package LEA (v3.3.2)^117^ to investigate the relationship between allele frequencies and environment variables. Utilizing the clusters identified through ADMIXTURE (v1.3.0)^108^, we performed LFMM analysis with four latent factors to account for population structure within the genotype data. For each environment variable, we performed five independent MCMC runs with 5,000 iterations as burn-in followed by 10,000 iterations. The resulting *P* values were averaged and adjusted for multiple testing using a false discovery rate (FDR) correction, with a significant cutoff 1%.

To investigate and compare the relative contributions of geographic and environmental factors to spatial genetic variation in *T. arundinaceum*, adaptive and neutral variant sets were analyzed separately. Adaptive variants comprised the core set identified by both RDA and LFMM, whereas neutral variants were represented by the LD-pruned subset used for population structure analysis. For each variant set, pairwise *F*_ST_ values among populations from different provinces were used as genetic distance. Geographical distances were calculated from the geographic coordinates of sampling sites. For environmental distances, the seven retained environmental variables were transformed using PCA, and Euclidean distances based on the first five principal components were computed between province pairs. Mantel tests were used to assess the correlations between genetic distance and geographical or environmental distance, and partial Mantel tests were performed to evaluate the independent contribution of each factor while controlling for the other. All tests were conducted in the R package vegan^115^ using Pearson’s correlation coefficient with significance assessed by 999 permutations. Furthermore, we used partial RDA to quantify the relative contribution of geography (latitude and longitude) and environment (seven environmental variables mentioned above) in explaining the proportion of genetic variation. The significance of the explanatory variables was evaluated using 999 permutations with the *anova.cca* function from the R package vegan^115^. The identified genes associated with core adaptive variants were functional annotated by aligning to NR database using BLASTP (v2.10.0 + ) (*E*-value < 1 × 10^−5^). LDBlockShow^118^ was employed to visualize the LD heatmap of SNPs surrounding candidate genes, utilizing the variant call format (VCF) file of adaptive variants.

### Reporting summary

Further information on research design is available in the Nature Portfolio Reporting Summary linked to this article.

## Supplementary information

Supplementary Information

Peer Review file

Description of Additional Supplementary Files

Supplementary Data 1

Supplementary Data 2

Supplementary Data 3

Supplementary Data 4

Supplementary Data 5

Supplementary Data 6

Supplementary Data 7

Supplementary Data 8

Reporting Summary

## Source data

Source Data

## Data Availability

All raw sequencing data and assembled genome sequences for *T. arundinaceum* were deposited into the National Center for Biotechnology Information (NCBI) Sequence Read Archive under BioProject accession PRJNA1182283. Genome assembly and annotation files of *T. arundinaceum* are additionally available in the Genome Warehouse of the National Genomics Data Center (NGDC) database under accession GWHGGGG00000000.1 [https://ngdc.cncb.ac.cn/gwh/Assembly/98824/show]. Source data are provided with this paper.
