# Supplementary material for: Haplotype-resolved genome assembly sheds light on the evolutionary history of autohexaploid Tripidium arundinaceum
Source: Nat Commun. 2026 Jun 27;17:8348. doi: 10.1038/s41467-026-74742-1 (PMC13473609; doi:10.1038/s41467-026-74742-1)
Supplement: Supplementary file 1 [file 41467_2026_74742_MOESM12_ESM.pdf]

nature portfolio

Corresponding author(s): Jisen Zhang

Last updated by author(s): YYYY-MM-DD

Reporting Summary

Nature Portfolio wishes to improve the reproducibility of the work that we publish. This form provides structure for consistency and transparency in reporting. For further information on Nature Portfolio policies, see our Editorial Policies and the Editorial Policy Checklist.

Statistics

For all statistical analyses, confirm that the following items are present in the figure legend, table legend, main text, or Methods section.

n/a	Confirmed	
☐	☒	The exact sample size (*n*) for each experimental group/condition, given as a discrete number and unit of measurement
☐	☒	A statement on whether measurements were taken from distinct samples or whether the same sample was measured repeatedly
☐	☒	The statistical test(s) used AND whether they are one- or two-sided<br>*Only common tests should be described solely by name; describe more complex techniques in the Methods section.*
☒	☐	A description of all covariates tested
☐	☒	A description of any assumptions or corrections, such as tests of normality and adjustment for multiple comparisons
☐	☒	A full description of the statistical parameters including central tendency (e.g. means) or other basic estimates (e.g. regression coefficient) AND variation (e.g. standard deviation) or associated estimates of uncertainty (e.g. confidence intervals)
☐	☒	For null hypothesis testing, the test statistic (e.g. *F*, *t*, *r*) with confidence intervals, effect sizes, degrees of freedom and *P* value noted<br>*Give P values as exact values whenever suitable.*
☒	☐	For Bayesian analysis, information on the choice of priors and Markov chain Monte Carlo settings
☒	☐	For hierarchical and complex designs, identification of the appropriate level for tests and full reporting of outcomes
☐	☒	Estimates of effect sizes (e.g. Cohen's *d*, Pearson's *r*), indicating how they were calculated

Our web collection on statistics for biologists contains articles on many of the points above.

Software and code

Policy information about availability of computer code

Data collection	PacBio CCS (Circular Consensus Sequencing) reads were undertaken with PacBio Sequel II platform; Hi-C reads were collected from the Illumina HiSeq X platform and paird-end Illumina sequencing were collected from the Illumina HiSeq and DIPSEQ platform.
Data analysis	The softwares used in this study have been well described in the method. The softwares are listed as follows: Jellyfish v2.2.6, Hifiasm v0.19.6-r595, HapHiC, BWA v0.7.17, minimap2 v2.26, PanDepth, mosdepth v0.3.5, Merqury v1.3, BUSCO, compleasm, MCScanX, JCVI, RepeatModeler (open-1.0.8), RepeatMasker (open-4.0.5), LTR_retriever v2.6, LTR_FINDER, LTRharvest, BRAKER, GeneMark-ETP, AUGUSTUS, AlleleFinder, BLASTP v2.10.0+, EggNOG-Mapper, KofamKOALA, DeepSignal-plant v.0.1.6, Guppy, tombo, Trinity v2.15.1, RSEM, Orthofinder v3.0.1b1, MUSCLE, RAxML v8.12, PAML 4, CAFÉ5, GetOrangelle, MAFFT v7.505, JModelTest2, RAxML-NG, Trimmomatic v0.39, Sentieon, VCFtools v0.1.18, SnpEff v4.3t, PLINK v1.90, PopLDdecay v3.31, ADMIXTURE v1.3.0, vcf2phylip v2.0, ANGSD, Stairway plots, SIFT4G, Maxent v3.4.4, usdm v2.1.7, vegan v2.7-1, LEA v3.3.2, LDBlockShow.

For manuscripts utilizing custom algorithms or software that are central to the research but not yet described in published literature, software must be made available to editors and reviewers. We strongly encourage code deposition in a community repository (e.g. GitHub). See the Nature Portfolio guidelines for submitting code & software for further information.

Data

Policy information about availability of data

All manuscripts must include a data availability statement. This statement should provide the following information, where applicable:

- Accession codes, unique identifiers, or web links for publicly available datasets
- A description of any restrictions on data availability
- For clinical datasets or third party data, please ensure that the statement adheres to our policy

All raw sequencing data for Tripidium arundinaceum were deposited into the NCBI Sequence Read Archive under the BioProject accession number PRJNA1182283. The genome assembly and annotation files of T. arundinaceum have been deposited in the Genome Warehouse of the National Genomics Data Center (NGDC) database (https://ngdc.cncb.ac.cn/gwh).

Research involving human participants, their data, or biological material

Policy information about studies with human participants or human data. See also policy information about sex, gender (identity/presentation), and sexual orientation and race, ethnicity and racism.

Reporting on sex and gender	NA
Reporting on race, ethnicity, or other socially relevant groupings	NA
Population characteristics	NA
Recruitment	NA
Ethics oversight	NA

Note that full information on the approval of the study protocol must also be provided in the manuscript.

Field-specific reporting

Please select the one below that is the best fit for your research. If you are not sure, read the appropriate sections before making your selection.

- [x] Life sciences
- [ ] Behavioural & social sciences
- [ ] Ecological, evolutionary & environmental sciences

For a reference copy of the document with all sections, see nature.com/documents/nr-reporting-summary-flat.pdf

Life sciences study design

All studies must disclose on these points even when the disclosure is negative.

Sample size	Samples were selected from wild accessions with different genetic backgrounds and geographical distributions to investigate meaningful results.
Data exclusions	No data were excluded.
Replication	For RNA-seq analysis of different tissues at different developmental stages, as well as leaf gradients and diurnal cycles, three biological replicates for each sample were performed, and each biological replicate contained samples from three plants. The phylogenetic tree based on SNPs was constructed with 1,000 bootstrap replicates. The FISH experiments were repeated independently at least three times with similar results. For LFMM, five independent MCMC runs were performed for each environmental variable, with 5,000 iterations used as burn-in followed by 10,000 iterations.
Randomization	This is not relevant to our study as all of the analyses in the research followed well-established methods.
Blinding	The investigators were blinded to group allocation during data collection.

Reporting for specific materials, systems and methods

We require information from authors about some types of materials, experimental systems and methods used in many studies. Here, indicate whether each material, system or method listed is relevant to your study. If you are not sure if a list item applies to your research, read the appropriate section before selecting a response.

Materials & experimental systems

n/a	Involved in the study
x	Antibodies
x	Eukaryotic cell lines
x	Palaeontology and archaeology
x	Animals and other organisms
x	Clinical data
x	Dual use research of concern
	x Plants

Methods

n/a	Involved in the study
x	ChIP-seq
x	Flow cytometry
x	MRI-based neuroimaging

Dual use research of concern

Policy information about dual use research of concern

Hazards

Could the accidental, deliberate or reckless misuse of agents or technologies generated in the work, or the application of information presented in the manuscript, pose a threat to:

No	Yes
x	Public health
x	National security
x	Crops and/or livestock
x	Ecosystems
x	Any other significant area

Experiments of concern

Does the work involve any of these experiments of concern:

No	Yes
x	Demonstrate how to render a vaccine ineffective
x	Confer resistance to therapeutically useful antibiotics or antiviral agents
x	Enhance the virulence of a pathogen or render a nonpathogen virulent
x	Increase transmissibility of a pathogen
x	Alter the host range of a pathogen
x	Enable evasion of diagnostic/detection modalities
x	Enable the weaponization of a biological agent or toxin
x	Any other potentially harmful combination of experiments and agents

Plants

Seed stocks	The 151 Tripidium arundinaceum accessions were supplied by Yunnan Academy of Agricultural Sciences, Kaiyuan, China; 2 Tripidium arundinaceum accessions were supplied by Fujian Agriculture and Forestry University, Fuzhou, China.
Novel plant genotypes	NA
Authentication	The re-sequencing data aligned to Hainan92-105 genome confirms the Tripidium arundinaceum species.
