# Supplementary material for: Haplotype-resolved genome assembly sheds light on the evolutionary history of autohexaploid Tripidium arundinaceum
Source: Nat Commun. 2026 Jun 27;17:8348. doi: 10.1038/s41467-026-74742-1 (PMC13473609; doi:10.1038/s41467-026-74742-1)
Supplement: Supplementary file 2 [file 41467_2026_74742_MOESM2_ESM.pdf]

nature portfolio

Peer Review File

Haplotype-resolved genome assembly sheds light on the evolutionary history of autohexaploid Tripidium arundinaceum

Corresponding Author: Dr Jisen Zhang

This file contains all reviewer reports in order by version, followed by all author rebuttals in order by version.

Version 0:

Reviewer comments:

Reviewer #1

(Remarks to the Author)
In the manuscript entitled:Haplotype-Resolved Genome Assembly Sheds Light on the Evolutionary History of Autohexaploid Tripidium arundinaceum and the Saccharum Complex, the authors present an annotated genome assembly for T. arundinaceum, a closely related species to sugarcane and Sorghum. While the authors have included a large number of analyses (eg. assembly, annotation, expression patterns, demographic/phylogenetic history, popgen, etc..) the main issue with the manuscript is the lack of detail when communicating methods and results throughout the entirety of the paper. While reading through the manuscript, I found myself wondering how exactly analyses were completed. For example: Figure 2 shows synteny among chromosomes and between species S.bicolor and E. rufipilus, but nowhere in the methods is this described. The authors acknowledge that many chimeric contigs existed in their genome assembly, but used HiC contact signal to correct them, as well as a 'comprehensive evaluation' of assembly methods. No details regarding what this entailed or how it was done are provided. ~19X HiFi is low coverage for an auto-hexaploid so I am certain that misjoins were very frequent in the assembly. Another example is genome annotation. The method is described in another paper and a 'perl script' was used to analyze and finalize the annotation. As written, it is impossible to evaluate the work presented here, and the examples provided above are by no means an exhaustive list. In order to be considered for publication, this manuscript requires extensive revision. My advice for authors is to focus on a smaller number of well-supported analyses rather than stretching themselves too thin.

Reviewer #2

(Remarks to the Author)
This manuscript introduces a new chromosome-level assembly of Tripidium arundinaceum, a species that represents a key ancestor of sugarcane and related species, and which may offer a novel resource for identifying genetic mechanisms abiotic stress tolerance that could be bred into domesticated sugarcane. The genome that is presented here appears to be highly contiguous of high quality, and the methods are explained in detail and are appropriate for all of the analyses being done. The results seem to offer some novel insights into the origins and evolutionary history of this species, especially with respect to environmental adaptations.

While this manuscript undoubtedly provides a lot of descriptive information about this new genome, I did at times find it hard to determine the relevance or context for a lot of the results that were presented, and I'm not sure why some of the analyses were done at all or what they are supposed to be telling me about the evolution of this species or its relationship to sugarcane (which seem to be the key questions at hand). I believe that the paper could benefit from some streamlining to focus on the most key results that relate back to some hypothesis about the differences between T. arundinaceum and its other relatives in Saccharum, and that there are a lot of sections which could either be removed altogether or shifted to supplementary information in order to make the paper more interesting and accessible to a wider audience. More specifically:

1. The "Genome assembly" results section contains a lot of information that could probably be moved to a supplement (or in some cases might need to be moved to the Methods section, for example, the paragraph at Lines 118-132).
2. What is the significance of the allele-specific expression analysis described starting at line 146? Why do a GO analysis of

the non-neutral genes? What is the hypothesis or expectation here related to how this might differentiate this species from its other relatives in the genus?
3. Similarly, what is the significance of the methylation analysis, since it seems to only be performed under a single condition?
4. There is a lot of discussion about the analysis of photosynthetic genes and the C4 photosynthetic pathway, but while I understand the general importance of photosynthesis as an agronomic trait, I don't really understand the purpose or the expected outcome for this analysis. Was there a prior hypothesis that T. arundinaceum would show significant differences in the photosynthetic pathway-related genes? Because it seems like these would actually be quite conserved. Was there debate as to whether or not T. arundinaceum was a C4 (versus a C3) plant? If these analyses are going to be included in the manuscript, I believe there should be some context given for them.

Some other, more minor comments and questions:

5. In the LFMM analysis, was population structure accounted for? I did not see this in the Methods (apologies if I missed it), but it does seem like there are a large number of significant SNPs found, so I wonder if many of these could be false positives.
6. In the paragraph discussing the timing of the expansions and contractions (lines 367-380), do any of these events seem to correspond to the timing(s) of the TE bursts?
7. Do the methods described in lines 678-685 also describe how the sugar pathway and C4 photosynthetic genes were identified? Or was there a separate orthology identification pathway (it seems like there must have been in order to perform the CAFÉ analysis)?
8. One lines 744-746 the authors mention using SnpEff to annotate variants. It's unclear if SnpEff was also used to identify nonsynonymous variants, but if it was then I would caution the authors that I've seen firsthand SnpEff provide some incorrect calls of nonsynonymous vs synonymous when you use it with a custom gff/gene file, so I recommend checking the results against the SIFT results, or just relying entirely on SIFT for the nonsynonymous vs synonymous annotations.
9. Figure 3b is pretty hard to read; I recommend giving the figure more space and moving the text labels to the right such that they don't overlap with the curves.
10. Figure 3d seems to show that only certain families of TEs (the Copia elements and the unknown elements) really show the bimodal distribution that strongly indicates a more recent "burst" of TE duplications. This seems interesting and might be worth noting in the manuscript.
11. In Figure 4C, it is very hard to see some of the points on the map because the colors of the points blend so well with the background (the blue points of Group III in particular are almost impossible to see). It seems like either the elevation information should be removed (so the map is just a line map), or the color scheme needs to be changed so the points are visible.

Version 1:

Reviewer comments:

Reviewer #1

(Remarks to the Author)
While I do think that this revised manuscript is improved over the previous submission, I find the same issues (vague statements and lack of detail) persist throughout. Results are typically reported in relative terms (higher, lower) than absolutes with vague interpretations attached ("suggesting evolutionary expansion", " suggesting rapid evolution", etc..), and the discussion largely re-states the results. Below I include two examples where key details are missing from reported results:

"Additionally, we discovered five TraDREB genes whose expression responded to drought stress, suggesting they may play important roles in drought tolerance (Supplementary Fig. 19). These findings provide a genetic foundation for the introgression of beneficial gene resources into sugarcane breeding." -There is no detail at all given here or in the methods about this analysis or result.

"In our study, the candidate adaptive SNP located in the upstream region of CYP78A5 was significantly associated with BIO7 (Temperature Annual Range) (Fig. 6a, d). Allele frequency analysis exhibited that the C allele was predominantly found in regions with a high temperature annual range, while the T allele was nearly fixed in areas with low temperature (Fig. 6e)".- Where is the SNP relative to the gene? are they in LD? what are temperature ranges? These details should be included in the text, not found in figure captions.

Reviewer #2

(Remarks to the Author)
In the revised version of the manuscript the authors have significantly streamlined the manuscript and provided thorough responses to all of my concerns.

Version 2:

Reviewer comments:

Reviewer #1

(Remarks to the Author)
As noted from the previous revision, I appreciate the authors providing additional information and context for their results (providing absolute values, etc..) but while reading the document, i was almost immediately perplexed by the use of statistics and reporting. On line 117, the authors report a significant size difference among T. arundinaceum and Saccharum ($P < 0.01$, Wilcoxon rank sum test). But based on the numbers provided above (1.14 Gb, 0.79 Gb, etc..), there is not enough power to detect a statistical difference. What is the test statistic for this Wilcoxon test? Is the monoploid size for each of the subgenome chromosome copies being tested against combined subgenomes within the other genomes? Looking at Supplementary figure 4, that appears to be the case, as every pairwise combination of Saccharum species relative to T.a is significant (T.a vs LA purple, T.a vs FuGuo1). Regardless, there are no test statistics (W values, degrees of freedom) reported throughout the manuscript.

Again, the same spurious connections exist throughout the manuscript. Here is just one example:
Our comparative genomic analyses revealed that T. arundinaceum harbors a significantly higher ($P < 0.0001$, Wilcoxon rank sum test) burden of deleterious mutations than S. spontaneum. Moreover, genes carrying deleterious mutations in T. arundinaceum were primarily enriched in biological processes crucial for development and stress responses. These results imply that the accumulation of deleterious mutations may have compromised key adaptive pathways, potentially influencing phenotypic robustness and environment adaptability of T. arundinaceum.

1)There is no evidence to link expanded genome size to elevated mutational load.
2)Also, is there an expectation that phenotypic robustness and environmental adaptability are potentially compromised in T. arundinaceum? In the introduction and discussion, the authors argue that the species is useful for its 'exceptional adaptability'. Wouldnt the rationale be that the increased mutational load stems from the bottleneck events, and not a larger genome? And the unexpected result (exceptional adaptation + high mutational load) be worthy of discussion?

Lastly, I looked for the evidence that supported the TraDREB genes playing a role in drought stress (a point I brought up before): From the manuscript:
Additionally, we discovered five TraDREB genes whose expression responded to drought stress, suggesting they may play important roles in drought tolerance (Supplementary Fig. 19; Supplementary Note 5). These findings provide a genetic foundation for the introgression of beneficial gene resources into sugarcane breeding.

Supp Note 5 reads: Quantitative real-time PCR analyses revealed that TraDREB1C, TraDREB1J, TraDREB2A, TraDREB2B, and TraDREB2C were up-regulated in response to drought stress (Supplementary Fig. 19).

Supp figure 19 shows the relative expression of the genes listed above at different time points (0h, 3h,6h, 9h) with the caption: Relative levels of gene expression of six TraDREBs in the leaves of T. arundinaceum HN92-105 under drought conditions. Details are pretty sparse here.. relative to what exactly? The zero timepoint? "CK" which is listed in the figure but not defined. Is 3 hours post nutrient removal considered drought stress? Regardless, this is not a demonstration of drought-upregulation, as there is no drought-free condition used as a control here. This expression pattern could just as easily be associated with circadian rhythm or something else entirely.

Version 3:

Reviewer comments:

Reviewer #3

(Remarks to the Author)
For lines 117–121, the test is actually unnecessary here. Simply remove the word "significantly" and state that the genome is larger than theirs. Statistical tests are unnecessary in many instances, unless relevant conclusions are to be drawn based on their results. In my observation, the authors conducted numerous tests without deriving any corresponding conclusions from them.

Regarding the section on deleterious mutations, the authors compared different populations and drew conclusions such as “both were significantly lower than that in S. officinarum”. However, I consider these conclusions unreliable. Population data are scarcely comparable, as each population has its own unique evolutionary history and characteristics, making direct comparisons difficult. This is especially true for T. arundinaceum and sugarcane (S. officinarum), which diverged a long time ago—specifically, their split predates the divergence between sorghum and sugarcane. Therefore, such comparisons are meaningless. At most, the authors could have merely described the mutation rate of T. arundinaceum and similar metrics.

For lines 219–223, the authors state that the five TraDREB genes are not rhythm-related. However, the expression level of DREB2B is clearly much higher during the day than at night, and this finding is not described anywhere in the full text. In addition, the RT-PCR results of these five genes appear questionable: some are upregulated while others are downregulated, and their expression trends are inconsistent. The reliability of these results needs to be further verified, for

example, by using RNA-seq data for cross-validation.

There are several serious inaccuracies. For instance, the abstract mentions T. arundinaceum's origin in northern India and its close affinity to sorghum, both of which are incorrect. The presence of such flawed conclusions in the abstract indicates a lack of rigor in the authors' findings. Specifically, sorghum and sugarcane are sister groups, while T. arundinaceum is an outgroup to the sorghum-sugarcane clade. It is therefore inappropriate to claim that T. arundinaceum is closely related to sorghum; in fact, it shares an equal degree of genetic relatedness with both sorghum and sugarcane. Furthermore, Extended Data Fig. 2c fails to support the assertion that T. arundinaceum originated in northern India. Notably, the genetic structure analysis in Extended Data Fig. 2a does not include any samples from northern India, making the conclusion about its northern Indian origin inexplicable.

Version 4:

Reviewer comments:

Reviewer #3

(Remarks to the Author)
The previous response is well-written, yet there remain a few issues to address. Given the mention of Saccharum Complex in the title, the authors are presumably referring to sugarcane (the crop) rather than the genus Saccharum per se. Notably, Tripidium is even more distantly related to sugarcane than sorghum is, making it unrealistic to investigate sugarcane by means of Tripidium. Similarly, the article repeatedly claims that Tripidium holds significance for sugarcane breeding, which is a rather tenuous assertion. By contrast, Tripidium does have certain reference value for studying the evolution of plants in the genus Saccharum.
Accordingly, the article should explicitly state that the Tripidium genome is used to elucidate the evolution of the genus Saccharum, rather than claiming that it provides reference value for sugarcane research.

Version 5:

Reviewer comments:

Reviewer #3

(Remarks to the Author)
Scientific research should first adhere to the principles of rigor, prudence, and reproducibility. Existing studies only suggest the possibility that Tripidium arundinaceum may be related to sugarcane or involved in introgression events with sugarcane, but there is no direct and conclusive experimental evidence to support this inference.
Although this study has assembled a high-quality genome of Tripidium arundinaceum, it has not demonstrated at the molecular or population level that genetic material from Tripidium arundinaceum has introgressed into sugarcane. Furthermore, the paper does not include any comparative analysis or validation experiments directly related to sugarcane. Under such circumstances, the background and title of the article overemphasize sugarcane, which is inconsistent with the actual research content and is likely to mislead readers. This does not conform to the prudent attitude required in scientific research.

There are numerous other instances of inadequate rigor throughout the manuscript.
For example, the abstract claims that Population genomics analysis revealed Yunnan Province as a diversity center for T. arundinaceum in China. However, Group III and Group IV contain very few accessions from Yunnan.
In contrast, Group II includes several accessions from Hainan, and Group III shows substantial introgression from Hainan. Notably, Hainan is located at the outermost position in the phylogeny, suggesting it may represent an earlier origin.

Given that sorghum is phylogenetically closer to sugarcane than T. arundinaceum,and given that the claim of introgression from T. arundinaceum into sugarcane remains highly questionable, I recommend that the authors remove all sugarcane-related background and content from the manuscript. In addition, the population genomic analyses of T. arundinaceum accessions should be completely re-analyzed, and all comparisons with sugarcane should be deleted.

Version 6:

Reviewer comments:

Reviewer #4

(Remarks to the Author)
Dear Editor,
After evaluating the manuscript and the authors' responses to Reviewer #3, we provide our formal assessment. While the haplotype-resolved assembly of Tripidium arundinaceum represents a significant technical achievement, the current

narrative framework contains major scientific inaccuracies and misleading interpretations that undermine the study's relevance.
We agree with Reviewer #3 that both the title and background overemphasize sugarcane and the concept of the Saccharum complex. This concept, first proposed by Mukherjee (1957), suggested that traditional sugarcane cultivars originated from interspecific hybridization among members of a putative interbreeding group comprising the genera Saccharum, Erianthus, Sclerostachya, and Narenga which he termed the 'Saccharum complex'. This group was later expanded by Daniels et al., 1975 to include Miscanthus.
However, molecular data do not support the involvement of Erianthus/Tripidium or Miscanthus in the emergence of sugarcane cultivars and thus their contribution to sugarcane genome. Instead, molecular data indicate that modern sugarcane emerged from wild Saccharum species. Moreover, to our knowledge, no natural hybrids between T. arundinaceum and sugarcane have been reported and no sugarcane cultivars that are grown commercially have been proven to incorporate chromosomes from T. arundinaceum. Due to the fact that some Tripidium arundinaceum have large, sugarcane-like stalks, there have been many attempts to use this species in sugarcane breeding. In fact, producing F1 and backcrosses between sugarcane and T. arundinaceum remains very difficult, and only a few of these events have been validated with molecular data. So, most breeding centers have abandoned this base broadening strategy and focus more on S. spontaneum.
In this context, the sentence of the abstract "Tripidium arundinaceum, a highly polyploid member of the Saccharum complex, has been used for over a century to broaden genetic diversity and enhance drought tolerance in sugarcane breeding" is inaccurate and misleading. The fact that Tripidium arundinaceum is phylogenetically quite distant from sugarcane, even more so than sorghum, could explain this lack of success in using Tripidium arundinaceum in sugarcane breeding.
This early concept of the Saccharum complex is misleading and should no longer be propagated.
Therefore, we agree that the background and title of the article overemphasize sugarcane and are misleading. We recommend removing the term "Saccharum complex" from the title and both the abstract and background should be modified to more accurately reflect current knowledge.
Regarding the claim that southwestern China (Yunnan) represents a "diversity center," the data presented in Figure 4 are difficult to interpret. A first difficulty arises from the fact that the admixture analysis (Figure 1b) includes only a subset of the accessions present in the phylogeny (Figure 1a). Furthermore, the identified group, except for group1, consist of both pure and hybrid accessions which are represented by dots with the same color in Figure 1c (map). So, the map fails to show how these pure and hybrid lineages are actually distributed. This ambiguity extends to the Principal Component Analysis (Figure 1d), which also appears to include only a fraction of the total samples (presumably only those considered "pure»?) further obscuring the global diversity picture. So, these figures should be modified.
In addition, the natural distribution of T. arundinaceum extends continuously across Southeast Asia and into northeastern India. Without including samples from this broader distribution, the observed diversity in Yunnan may simply reflect the edge of a wider genetic gradient rather than a localized diversification hotspot.
So, we agree with reviewer 3, with the current presented data the claim of a "diversity center," is not sufficiently supported.
There are several other weaknesses in the studies in particular we have reservations regarding some of the population genomic methodologies used, specifically concerning the tools and parameters used to account for the polyploid nature of T. arundinaceum.
Best regards,

Version 7:

Reviewer comments:

Reviewer #4

(Remarks to the Author)
Dear Editor,

We appreciate the authors' constructive responses to our previous comments, as well as the revisions made to the manuscript. To ensure complete consistency and accuracy before publication, we request the following final adjustments:

Abstract (line 26): Please replace the term "employed" with "tested" to avoid any misunderstanding and the very limited success in the use of Tripidium arundinaceum in commercial sugarcane improvement.

Introduction (line 50) and Discussion (line 394): Please cite Welker et al. (2019; DOI: 10.1002/tax.12030) alongside the currently cited Lloyd Evans et al. (2019), as both groups independently reassigned Erianthus arundinaceus to Tripidium in the same year.

(lines 71–72): The statement that "only very few commercially grown sugarcane cultivars have been conclusively shown to carry chromosomes derived from T.arundinaceum" must be supported by a specific reference.

(line 404): The sentence stating that "E. rufipilus considered the last common ancestor of Saccharum" is not clear: last common ancestor between which taxons ? and scientifically inaccurate. E. rufipilus (= E. fulvus) and Saccharum diverged from a common ancestor approximately 2–2.5 Mya (Wang et al. 2023).

Figure 4b: Although the clarifications have improved the interpretation of the population genomics data, the names of the accessions must be identifiable in the admixture bar plot (Figure 4). If the image resolution does not allow for this, a

Supplementary Table listing the exact order of the samples as they appear in the plot must be provided and referenced in the text where Figure 4 is discussed.

Best regards,

Open Access This Peer Review File is licensed under a Creative Commons Attribution 4.0 International License, which permits use, sharing, adaptation, distribution and reproduction in any medium or format, as long as you give appropriate credit to the original author(s) and the source, provide a link to the Creative Commons license, and indicate if changes were made.
In cases where reviewers are anonymous, credit should be given to 'Anonymous Referee' and the source.
The images or other third party material in this Peer Review File are included in the article's Creative Commons license, unless indicated otherwise in a credit line to the material. If material is not included in the article's Creative Commons license and your intended use is not permitted by statutory regulation or exceeds the permitted use, you will need to obtain permission directly from the copyright holder.
To view a copy of this license, visit https://creativecommons.org/licenses/by/4.0/

REVIEWER COMMENTS

Reviewer #1 (Remarks to the Author):

In the manuscript entitled: Haplotype-Resolved Genome Assembly Sheds Light on the Evolutionary History of Autohexaploid Tripidium arundinaceum and the Saccharum Complex, the authors present an annotated genome assembly for T. arundinaceum, a closely related species to sugarcane and Sorghum. While the authors have included **a large number of analyses** (eg. assembly, annotation, expression patterns, demographic/phylogenetic history, popgen, etc..) the main issue with the manuscript is the lack of detail when communicating methods and results throughout the entirety of the paper. While reading through the manuscript, I found myself wondering how exactly analyses were completed. For example: Figure 2 shows synteny among chromosomes and between species S.bicolor and E. rufipilus, but nowhere in the methods is this described. The authors acknowledge that many chimeric contigs existed in their genome assembly, but used HiC contact signal to correct them, as well as a 'comprehensive evaluation' of assembly methods. No details regarding what this entailed or how it was done are provided. ~19X HiFi is low coverage for an auto-hexaploid so I am certain that misjoins were very frequent in the assembly. Another example is genome annotation. The method is described in another paper and a 'perl script' was used to analyze and finalize the annotation. As written, it is impossible to evaluate the work presented here, and the examples provided above are by no means an exhaustive list. In order to be considered for publication, this manuscript requires extensive revision. My advice for authors is to focus on a smaller number of well-supported analyses rather than stretching themselves too thin.

We sincerely appreciate your professional comments and insightful suggestions. To address your concerns more specifically, we have organized our responses into the following points:

Q1. In the manuscript entitled: Haplotype-Resolved Genome Assembly Sheds Light on the Evolutionary History of Autohexaploid *Tripidium arundinaceum* and the Saccharum

Complex, the authors present an annotated genome assembly for *T. arundinaceum*, a closely related species to sugarcane and Sorghum. While the authors have included **a large number of analyses** (eg. assembly, annotation, expression patterns, demographic/phylogenetic history, popgen, etc..) the main issue with the manuscript is the lack of detail when communicating methods and results throughout the entirety of the paper.

Response 1: Thank you for your constructive feedback. We have made comprehensive revisions throughout the manuscript to address your concerns, particularly regarding the methodology and results sections (See Response 5).

Q2. While reading through the manuscript, I found myself wondering how exactly analyses were completed. For example: Figure 2 shows synteny among chromosomes and between species *S. bicolor* and *E. rufipilus*, but nowhere in the methods is this described.

Response 2: We appreciate the reviewer's valuable comment regarding methodological clarity. In response, we have added detailed descriptions of some analyses in the manuscript. For example, in the section titled "Comparative Genome Analysis and Divergence Time Assessment" (Lines 541-545), we describe the whole-genome synteny analysis performed among *Tripidium arundinaceum*, *S.bicolor*, and *E. rufipilus*: "We performed a whole-genome synteny analysis among *T. arundinaceum* HN92-105, *S. bicolor* (https://phytozome.jgi.doe.gov/info/Sbicolor_v5_1), *E. rufipilus*[1], and *Saccharum* (*S. spontaneum*: Np-X[2] and AP85-441[3]; *S. officinarum*: Fuguo1 and LA-purple (https://sugarcane.gxu.edu.cn/scdb/)) using MCScanX[4] and JCVI[5] with default parameters."

Q3. The authors acknowledge that many chimeric contigs existed in their genome assembly, but used HiC contact signal to correct them, as well as a 'comprehensive evaluation' of assembly methods. No details regarding what this entailed or how it was done are provided. ~19X HiFi is low coverage for an auto-hexaploid so I am certain that misjoins were very frequent in the assembly.

Response 3: Indeed, ~19x HiFi is low coverage for an auto-hexaploid and may cause misjoins. Thus, to tackle the issue of substantial chimeric contigs in our genome assembly, we performed additional PacBio SMRT sequencing of *T. arundinaceum* HN92-105, generating approximately 238.15 Gb (~36x) of high-fidelity (HiFi) reads (Supplementary Table 1). Due to minor discrepancies between HiFi reads generated from the two sequencing runs could potentially affect the assembly software's assessment of homozygous read coverage, possibly leading to an overestimation of the genome size. Consequently, we performed de novo assembly using only the newly generated HiFi reads, while leveraging the older HiFi dataset solely for enhancing assembly contiguity (see Methods for details, Lines 515-525). The final results of the genome assembly showed significant improvement. With comparison to previous version, the upgraded genome assembly showed an increase in contig N50 from 167 Kb to 30.5 Mb, and a reduction in the number of gaps from 85,039 to 506 (Response-Table 1). The improved genome of *T. arundinaceum* demonstrates significantly higher quality compared to the genome of *Saccharum*[2,6,7] (Response-Table 2), further validating the reliability of the data presented in this study.

Supplementary Table 1 Statistics of sequencing reads used for *T. arundinaceum* HN92-105 genome assembly.

Items	Pacbio HIFI		Illumina	Hi-C
	older	newly		
Total Num. of sequencing Cell (Library)	6	2	1	4
Total Num. of reads	6,872,879	14,495,947	2,892,343,624	4,257,772,728
Total Num. of sequenced Base (Mp)	123,726	238,152	433,852	636,864
Mean reads length (bp)	18,002	16,429	150	150
Coverage (×)	~19	~36	~65	~95

Response-Table 1 Statics of the *T. arundinaceum* genome assembly before and after additional sequencing.

Items	Canu corrected phased assembly (before)	Hifiasm assembly (after)
Assembly size of contigs (bp)	6,719,955,602	6,722,710,240
Number of contigs	105,447	2,097
Maximum length (bp)	6,797,000	90,755,854
N50 contig (bp)	166,999	30,600,032
N90 contig (bp)	36,000	6,027,988
Average length (bp)	63,728	3,205,870
Total assembly size of scaffolds (bp)	6,728,459,502	6,722,760,840
Number of scaffolds	20,408	1,591
Maximum length (bp)	132,338,406	145,881,694
Average length (bp)	329,697	4,225,494
N50 scaffold length (bp)	111,839,775	104,813,120
N90 scaffold length (bp)	83,999,130	88,420,611
Number of chromosomes	60	60
Anchored sequences (bp)	6,467,142,292	6,465,188,122
Number of gaps	85,039	506

Response-Table 2 Comparison of Genome Assembly Results between *T. arundinaceum* and *Saccharum*.

Genome assembly	*T. arundinaceum* HN92-105	*S.spontaneum* Np-X	*S.* hybrid XTT22	*S.* hybrid R570	
Assembled genome size (Gb)	6.72	2.76	9.28	Primary	5.04
				Alternate	3.73
N50 contig (Mb)	30.6	0.38	0.51	Primary	12.6
				Alternate	2.1
Number of chromosomes	60	40	97	86	
Gaps (% of Genome)	0.001%	0.053%	0.049%	0.100%	

Q4. Another example is genome annotation. The method is described in another paper and a 'perl script' was used to analyze and finalize the annotation.

Response 4: Furthermore, we have provided a distinct software pipeline and methodology for gene model prediction of *T. arundinaceum* HN92-105, with comprehensive details

included in the Methods section (Lines 562-572): “Gene model prediction was performed using the BRAKER3[8] pipeline (https://github.com/Gaius-Augustus/BRAKER), incorporating RNA-seq data from root, stem, and leaf tissues, as well as protein sequences from *O. sativa* (https://phytozome-next.jgi.doe.gov/info/Osativa_v7_0), *S. bicolor* (https://phytozome-next.jgi.doe.gov/info/Sbicolor_v3_1_1), and *E. rufipilus*[1]. High-confidence gene models were generated through training and prediction with GeneMark-ETP[9] and AUGUSTUS[10]. UTR regions were predicted from RNA-seq data using the script stringtie2utr.py of BRAKER3”.

Q5. As written, it is impossible to evaluate the work presented here, and the examples provided above are by no means an exhaustive list. In order to be considered for publication, this manuscript requires extensive revision. My advice for authors is to focus on a smaller number of well-supported analyses rather than stretching themselves too thin.

Response 5: We sincerely appreciate your constructive feedback and fully agree that a more focused and well-supported analyses would strengthen the manuscript. In response to this concern, we have thoroughly revised the manuscript:

1) **Streamline our analyses** to focus on the most biologically and statistically robust findings. Such as, Clarifying the Phylogenetic Position and Taxonomic Status of *T. arundinaceum*; LTR-RTs Bursts Facilitated Environmental Adaptation; Genetic Base of Important Agronomic Trait for Breeding Improvement; Population Genomics Insights for Breeding Applications.

2) **Expand methodological details** to ensure all key procedures are clearly documented and reproducible (e.g., plant materials collect, synteny analysis, divergence time estimation, genome quality assessment, Gene model prediction, and identifications of genes which related to key agronomic characteristics).

3) **Remove peripheral or underdeveloped analyses** that may dilute the core findings to the Supplementary Notes (e.g., Genome quality assessment, Allelic expression dominance, DNA methylation, Gene family expansion and contraction, and Genes related to key agronomic characteristics).

Reviewer #2 (Remarks to the Author):

This manuscript introduces a new chromosome-level assembly of *Tripidium arundinaceum*, a species that represents a key ancestor of sugarcane and related species, and which may offer a novel resource for identifying genetic mechanisms abiotic stress tolerance that could be bred into domesticated sugarcane. The genome that is presented here appears to be highly contiguous of high quality, and the methods are explained in detail and are appropriate for all of the analyses being done. The results seem to offer some novel insights into the origins and evolutionary history of this species, especially with respect to environmental adaptations.

Response: Thank you for your positive comments. Sugarcane breeders have tried to use *Tripidium arundinaceum* as a resource to improve stress tolerance over hundred years. As an autohexaploid, *T. arundinaceum* presents certain challenges for the genomics study. In the revised manuscript, we have further improved the genome assembly by incorporating additional higher-coverage sequencing data (Table 1 and Supplementary Table 1). Based on the improved genome, we have revised the analyses and content throughout the manuscript, enhancing the logic and continuity of the manuscript, thereby making our research more robust and powerful.

While this manuscript undoubtedly provides a lot of descriptive information about this new genome, I did at times find it hard to determine the relevance or context for a lot of the results that were presented, and I'm not sure why some of the analyses were done at all or what they are supposed to be telling me about the evolution of this species or its relationship to sugarcane (which seem to be the key questions at hand). I believe that the paper could benefit from some streamlining to focus on the most key results that relate back to some hypothesis about the differences between *T. arundinaceum* and its other relatives in *Saccharum*, and that there are a lot of sections which could either be removed altogether or shifted to supplementary information in order to make the paper more interesting and accessible to a wider audience.

Response: We sincerely appreciate your valuable suggestions. We have streamlined the content by moving peripheral or underdeveloped analyses that may dilute the key findings to supplementary Notes (e.g., genome quality assessment, allele-specific expression, DNA methylation, gene family expansion and contraction, and genes related to key agronomic characteristics). We refocused the main text on the key biological questions central to this study:

1) Clarifying the Phylogenetic Position and Taxonomic Status of *Tripidium arundinaceum*

Using our haplotype-resolved genome assembly, we provide robust evidence that *T. arundinaceum* is more closely related to *Sorghum bicolor* than to *Saccharum*, resolving its long-debated classification. Divergence time estimates (~6.54 Mya) support its reclassification from *Erianthus* to *Tripidium* and explain the challenges of utilizing *T. arundinaceum* in sugarcane breeding due to its deep divergence from *Saccharum*.

2) LTR-RTs Bursts Facilitated Environmental Adaptation

We identified two major recent bursts of LTR retrotransposons in *T. arundinaceum*, both of these (~0.1 and 0.24 Mya) coincided with the population bottlenecks triggered by the extremely low temperatures and likely contributing to adaptation during glacial periods.

3) Genetic Base of Important Agronomic Trait for Breeding Improvement

We provide the first physiological evidence confirming that *T. arundinaceum* is a C_4 plant. Comparative genomic analysis identified key genes related to agronomic characteristics, including C_4 photosynthesis, sugar metabolism, and drought response. While these genes related to agronomic traits are generally conserved, variations in gene copy number and expression patterns (e.g., *PPDK-RP*, *DREBs*) between *T. arundinaceum* and *Saccharum* have been observed, potentially resulting in functional divergence. This provides a foundational framework for integrating beneficial genes into sugarcane breeding programs.

4) Population Genomics Insights for Breeding Applications

Demographic analyses suggest a possibility shared origin with *S. spontaneum* in northern India, with Yunnan Province as a genetic diversity center in China. Genotype-environment association analyses pinpoint key loci (e.g., *CYP78A5*, *bHLH112*) linked to local adaptation, providing targets for improving stress resilience in sugarcane.

By restructuring the manuscript around these core themes, we aim to present a clearer narrative that highlights the evolutionary, ecological, and breeding implications of our findings. We hope these revisions address your concerns and appreciate your time in reviewing our work.

More specifically:

Q1. The “Genome assembly” results section contains a lot of information that could probably be moved to a supplement (or in some cases might need to be moved to the Methods section, for example, the paragraph at Lines 118-132).

Response 1: Thank you for your valuable feedback. We have reassembled the *T. arundinaceum* genome, and in the updated manuscript, the “Genome assembly” section now emphasizes key results (Lines 96–118), detailed technical descriptions have been moved to the Method section (Lines 515-532).

Q2. What is the significance of the allele-specific expression analysis described starting at line 146? Why do a GO analysis of the non-neutral genes? What is the hypothesis or expectation here related to how this might differentiate this species from its other relatives in the genus?

Response 2: We appreciate your insightful questions regarding the significance of our allele-specific expression analysis and subsequent GO of the non-neutral genes (allele-specific expression genes). There are two reasons for us to performed these analyses:

1) While homologous expression bias has been reported in many allopolyploids, such as wheat[11-13] and cotton[14-16], its study in autopolyploids remains less understood. We

sought to determine whether autohexaploid *T. arundinaceum* exhibit similar patterns of allele-specific expression that might contribute to its phenotypic characteristics.

2) We hypothesized that any observed allele-specific expression patterns might reveal functionally important genes contributing to *T. arundinaceum*'s distinctive developmental or stress-response traits compared to *Saccharum*.

29.69% of genes exhibited allele-specific expression (non-neutral genes) across all the examined tissues in *T. arundinaceum*, whereas 62.4% non-neutral genes were observed in the *S. spontaneum* genome[3]. This suggests that *S. spontaneum* may experience more pronounced transgressive segregation than *T. arundinaceum* at the genetic level. GO enrichment analysis suggest that these non-neutral genes may play important role the process of defense and developmental in *T. arundinaceum*.

Upon careful reconsideration, we acknowledge that, although this analysis provided some interesting observations, its relevance to our primary findings and its comparative significance between *T. arundinaceum* and *Saccharum* require more extensive investigation than we can adequately present in this manuscript.

Therefore, in order to focus on the most robust and well-supported aspects of our research, we have decided to shift both the allele-specific expression analysis and the associated GO analysis to the Supplementary Notes (Lines 20-40 of Supplementary information). We believe this revision will enhance the clarity and overall impact of our study.

Q3. Similarly, what is the significance of the methylation analysis, since it seems to only be performed under a single condition?

Response 3: Thank you for this important question. In this study, we performed genome-wide methylation profiling to systematically explore the relationships among DNA methylation, TE activity, and gene expression levels in the *T. arundinaceum* genome. Our integrated multi-omics approach revealed that TE-associated methylation likely contributes to the suppression of nearby gene expression, providing key insights into the epigenetic regulation of the *T. arundinaceum* genome.

Since this study was not designed to investigate condition-specific methylation changes, we focused on profiling a single, representative condition to ensure a clear and interpretable foundational dataset. Future studies could build on these findings by examining how methylation patterns shift under different stresses or treatments.

Q4. There is a lot of **discussion about** the analysis of photosynthetic genes and the C4 photosynthetic pathway, but while I understand the general importance of photosynthesis as an agronomic trait, I don't really understand the purpose or the expected outcome for this analysis. Was there a prior hypothesis that T. arundinaceum would show significant differences in the photosynthetic pathway-related genes? Because it seems like these would actually be quite conserved. Was there debate as to whether or not T. arundinaceum was a C4 (versus a C3) plant? If these analyses are going to be included in the manuscript, I believe there should be some context given for them.

Response 4: We thank the reviewer for this valuable comment. Although *T. arundinaceum* has traditionally been grouped within the C_4 *Saccharum* complex, our phylogenomic and chloroplast genome analyses revealed that its clusters with other *Tripidium* species (e.g., *T. ravennae, T. kanashiroi*), forming a distinct lineage separate from *Saccharum* and more closely related to *Sorghum*. These findings suggest that, despite a shared C_4 photosynthetic strategy, regulatory features of the C_4 pathway may have diverged substantially in *T. arundinaceum* relative to other C_4 grasses. Moreover, there was previously no direct physiological evidence confirming that *T. arundinaceum* is a C_4 plant.

In this study, anatomical observations and C_4-type A-Ci curves confirmed that *T. arundinaceum* performs C_4 photosynthesis (Supplementary Fig. 13b). While core C_4 enzymes are generally conserved, variation in gene copy number, regulatory mechanisms, and transcriptional control can lead to functional divergence. Notably, our phylogenetic and expression analyses of *PPDK-RP* (pyruvate phosphate dikinase regulatory protein) indicate that *T. arundinaceum* lacks the canonical C_4-type *PPDK-RP1* found in other C_4 grasses. Instead, it harbors a single *PPDK-RP* homolog (*TraPPDK-RP*) that clusters with

non-C_4-type genes. In contrast, sugarcane, maize, and sorghum each contain three *PPDK-RP* paralogs, including a C_4-specialized *PPDK-RP1*. This suggests that the *PPDK-RP* tandem duplication observed in sorghum and other C_4 species likely occurred after they diverged from *T. arundinaceum*.

Despite this, expression profiling along a developmental leaf gradient showed that *TraPPDK-RP* exhibits an increasing expression trend toward the leaf tip, similar to the pattern observed for C_4-type *PPDK-RP1* in other species.

Together, these findings support the idea that while the enzymatic components of the C_4 cycle are conserved, regulatory modules such as *PPDK-RP* have evolved in a lineage-specific manner. This provides new insights into the diversity of C_4 regulation across grass species. We have incorporated this explanation into the revised Results section (lines 203–209) and added additional context to the Introduction (lines 80–82, 86-87). Since no further in-depth investigation was conducted on these findings, in the revised manuscript we primarily presented and discussed these results in the Results section without elaborating on them in the Discussion section. Additionally, to focus on the most key results, some redundant results were moved to Supplementary Notes (Lines 93-129 of Supplementary information).

Some other, more minor comments and questions:

Q5. In the LFMM analysis, was population structure accounted for? I did not see this in the Methods (apologies if I missed it), but it does seem like there are a large number of significant SNPs found, so I wonder if many of these could be false positives.

Response 5: Thanks for your comment. We did account for population structure in our analysis; however, this important detail was inadvertently omitted from the Methods section. We have now included this information in the section titled "Identification of environment associated genetic SNPs" (Lines 709-711): "Utilizing the clusters identified

through ADMIXTURE (v1.3.0), we performed LFMM analysis with four latent factors to account for population structure within the genotype data.”.

In diploid populations of *Populus koreana*, Sang *et al.* identified 1,779 core adaptive variants associated with local climate adaptation from 5,949,459 common variants[17]. In contrast, our study identified 7,592 high-confidence core adaptive variants from 12,597,036 common variants in *T. arundinaceum* populations. This difference likely related to the disparity in genome size (401.41 Mb in *P. koreana* vs 6.72 Gb in *T. arundinaceum* HN92-105), as larger genomes typically harbor more genetic variation. Moreover, the higher number of adaptive variants in *T. arundinaceum* may be further attributed to its polyploid nature, which could increase genetic diversity and the pool of potential adaptive mutations.

To minimize the false positive rate, we selected the common variants with a minor allele frequency (MAF) > 10% to identify genomic variants associated with environmental conditions in *T. arundinaceum*. By combining the results of redundancy analysis (RDA) with a standard deviation cutoff of 3 and the univariate latent-factor linear mixed model (LFMM) that applied false discovery rate (FDR) correction with a significance threshold of 1% for adjusting *P*-values, we utilized the identified intersecting loci as the final candidate sites.

Q6. In the paragraph discussing the timing of the expansions and contractions (lines 367-380), do any of these events seem to correspond to the timing(s) of the TE bursts?

Response 6: Thanks for this insightful question. Indeed, our analysis revealed temporal correlations between TE bursts and demographic changes in *T. arundinaceum*. In our study, we identified two major recent burst events of LTR retrotransposons in the genome of *T. arundinaceum*. The two bursts occurred at approximately 0.1 and 0.24 Mya, both coinciding with the unique demographic bottleneck in *N*e of *T. arundinaceum* during 0.1-0.2 Mya, which temporally overlaps with the dramatic temperature decline in the Guxiang Glaciation (0.3-0.13 Mya) (Response-Fig. 1). Based on these observations, we hypothesize

that *T. arundinaceum* may have adapted to harsh environments through those bursts of LTR-RTs. These findings could suggest a relationship between TE activity and significant demographic and climatic events that may have influenced the evolutionary history of *T. arundinaceum*.

We have included above descriptions in our Discussion section (Lines 388-395).

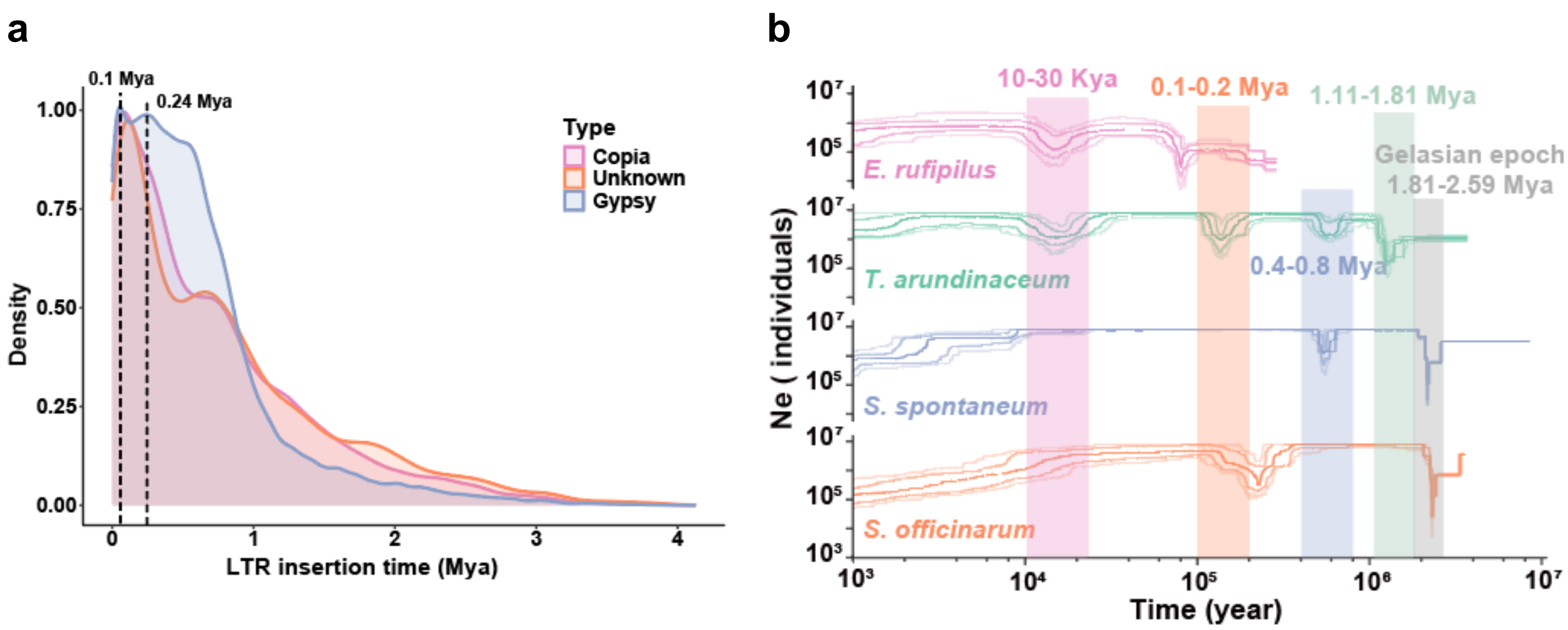

Response-Fig. 1 Analysis of intact LTR-RTs insertion times in the genomes of *T. arundinaceum* (a) and Demographic history of *T. arundinaceum*, *E. rufipilus*, *S. spontaneum* and *S. officinarum* (b).

Q7. Do the methods described in lines 678-685 also describe how the sugar pathway and C4 photosynthetic genes were identified? Or was there a separate orthology identification pathway (it seems like there must have been in order to perform the CAFÉ analysis)?

Response 7: We appreciate the reviewer's insightful question. The methods described in lines 678-685 (previous version) do not cover the identification of the sugar pathway and C_4 photosynthetic genes. For the identification of these important biologically relevant genes (including sugar pathway and C_4 photosynthetic genes), we have included the detailed methods as follows in our revised manuscript (Lines 604-612): "To identify genes associated with key agronomic traits in *T. arundinaceum*, we performed comparative analyses using reference species, including *O. sativa*, *Z. mays*, and *S. bicolor*. Protein sequences of these genes in reference species were retrieved from phytozome v13

(https://phytozome-next.jgi.doe.gov/) and NCBI (https://www.ncbi.nlm.nih.gov/), followed by BLASTP (*E*value of 1e^{-5}) against the *T. arundinaceum* genome to identify homologous genes. The counts of genes involved in the sugar pathway, photosynthetic pathway, and cellulose synthase for *O. sativa*, *Z. mays*, *S. bicolor*, and *Saccharum* were sourced from Zhang et al.[2], while data on *DREB* genes were collected from ref[18-22].".

Q8. One lines 744-746 the authors mention using SnpEff to annotate variants. It's unclear if SnpEff was also used to identify nonsynonymous variants, but if it was then I would caution the authors that I've seen firsthand SnpEff provide some incorrect calls of nonsynonymous vs synonymous when you use it with a custom gff/gene file, so I recommend checking the results against the SIFT results, or just relying entirely on SIFT for the nonsynonymous vs synonymous annotations.

Response 8: We appreciate the reviewer's valuable suggestion regarding potential inaccuracies in SnpEff's annotation of nonsynonymous variants when using custom gene annotations. To ensure robustness, we have already employed both SnpEff and SIFT for variant annotation in this study. The results from these tools were cross-validated to minimize misclassification errors. We have also revised the methods section to provide greater clarity on our approach (Lines 647-651): "The SnpEff[23] program was employed to annotate the variants based on gene models from HN92-105 genome annotation. The variant sites were annotated as the SNP and Indels, as well as intergenic and genic regions. Additionally, SIFT[24] was performed for nonsynonymous and synonymous annotations.".

Q9. Figure 3b is pretty hard to read; I recommend giving the figure more space and moving the text labels to the right such that they don't overlap with the curves.

Response 9: Thanks for your constructive feedback on the clarity of Figure 3b. Indeed, the presence of numerous made the original version appear cluttered and difficult to read. Following your suggestion, we have expanded figure layout to provide more space and repositioned the text labels to the right to prevent overlap with the curves. We prepared two revised two versions (Response-Fig. 2b and 2c) and, after discussing with the other

coauthors, we have decided to select Response-Fig. 2b as the final version due to its enhanced clarity and straightforwardness (Response-Fig. 2).

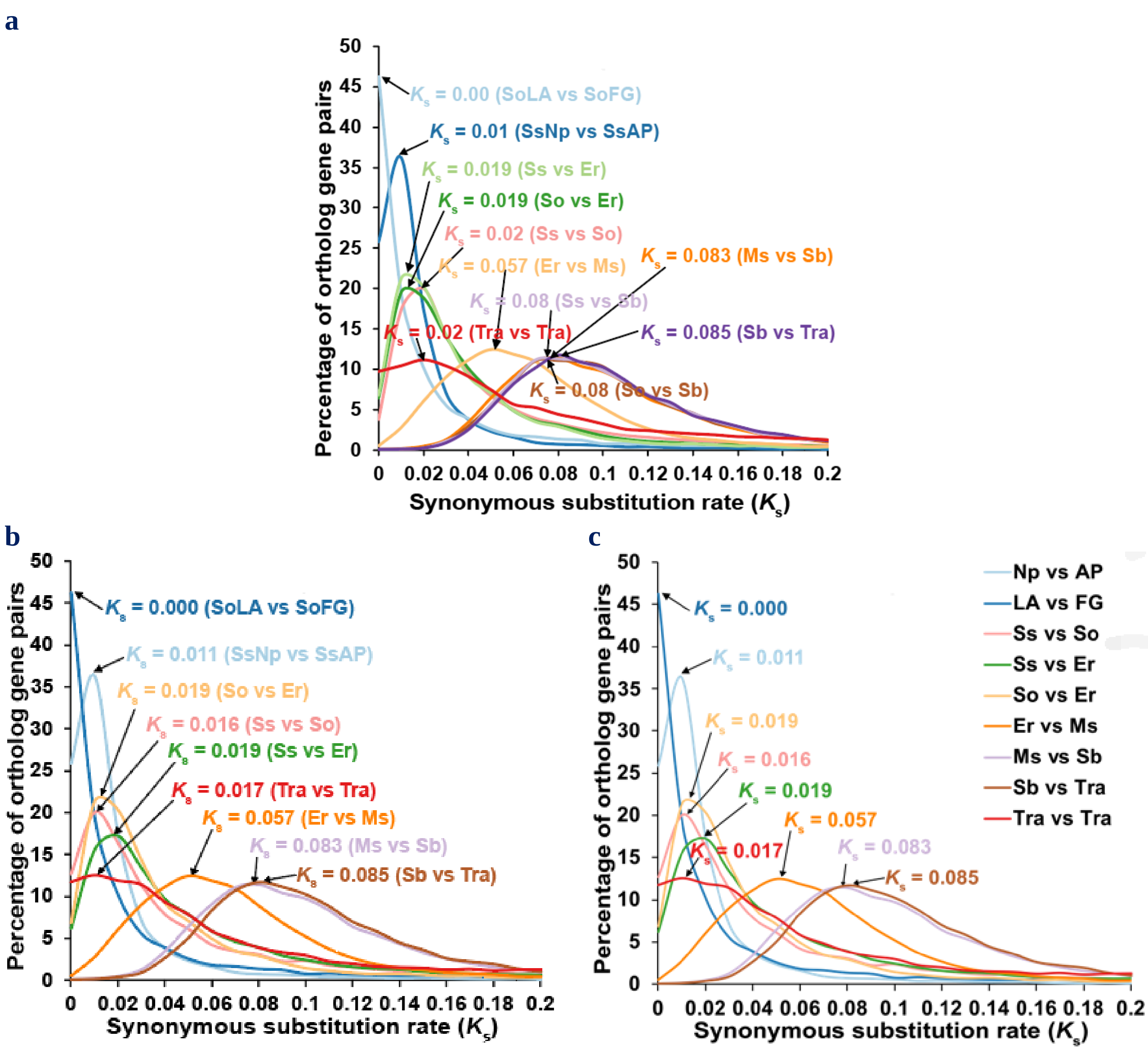

Response-Fig. 2 Original Figure 3b (a) and Revised Figure 3b (b, c). Distributions of synonymous substitution rate (K_s) between the representative species *S. officinarum* (LA-purple: SoLA, FuGuo1: SoFG), *S. spontaneum* (AP85-441: SsAP, Np-X: SsNp), *E. rufipilus* (Er), *Miscanthus* (Ms), *S. bicolor* (Sb), and *T. arundinaceum* (Tra).

Q10. Figure 3d seems to show that only certain families of TEs (the Copia elements and the unknown elements) really show the bimodal distribution that strongly indicates a more

recent "burst" of TE duplications. This seems interesting and might be worth noting in the manuscript.

Response 10: Thank you for your insightful comment. As you noted, the results presented in Figure 3d indicate that certain families of TE exhibit a bimodal distribution that strongly suggests recent bursts of TE duplications (Response-Fig. 3). We have explicitly highlighted this finding in the manuscript (Lines 139-144): "Analysis of the LTR-RTs insertion timing revealed two major recent burst events during the evolution of *T. arundinaceum*, occurring approximately 0.1 and 0.24 million years ago (Mya), respectively (Fig. 3d; Extended Data Fig. 1a). These successive waves of LTR-RTs proliferation likely contributed to the high abundance of transposable elements (TEs) observed in the *T. arundinaceum* genome.". And discussed its potential implications for the evolutionary dynamics of *T. arundinaceum* in Discussion section (Lines 388-397): "The *T. arundinaceum* genome experienced two distinct bursts of LTR-RTs activity following polyploidization, which likely contributed to its higher TE content within the *Saccharum* complex. Interestingly, these major TE burst events coincide with the unique demographic bottleneck in N_e of *T. arundinaceum*, which temporally overlaps with the dramatic temperature decline in the Guxiang Glaciation (0.3-0.13 Mya) (Figs. 3d and 5d). These findings suggest that *T. arundinaceum* may have adapted to harsh environments through bursts of LTR-RTs. Furthermore, the monoploid genome of *T. arundinaceum* is substantially larger than that of *Saccharum*, indicating that these TE bursts have played a significant role in the expansion of the *T. arundinaceum* genome.".

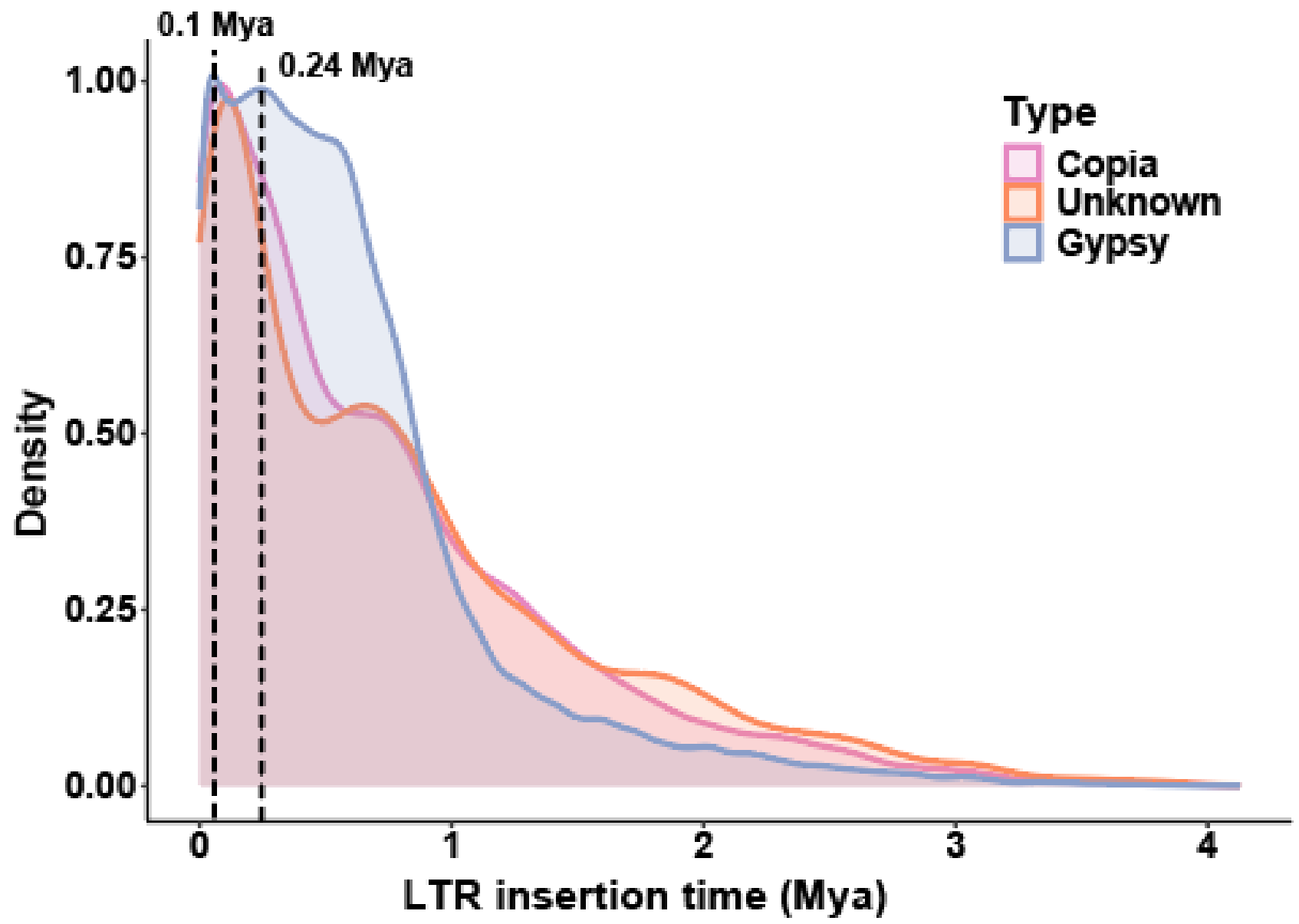

Response-Fig. 3 Analysis of intact LTR-RTs insertion times in the genomes of *T. arundinaceum*. The horizontal and vertical axes represent the insertion time of intact LTR retrotransposons and the density of intact LTR retrotransposons, respectively.

Q11. In Figure 4C, it is very hard to see some of the points on the map because the colors of the points blend so well with the background (the blue points of Group III in particular are almost impossible to see). It seems like either the elevation information should be removed (so the map is just a line map), or the color scheme needs to be changed so the points are visible.

Response 11: Thanks for your helpful suggestion. We agree that some data points in Figure 4c were difficult to distinguish due to the color scheme and elevation shading. To improve clarity, we have revised the figure by removing the elevation background, leaving only the line map. This adjustment ensures that all points are now clearly visible (Response-Fig. 4).

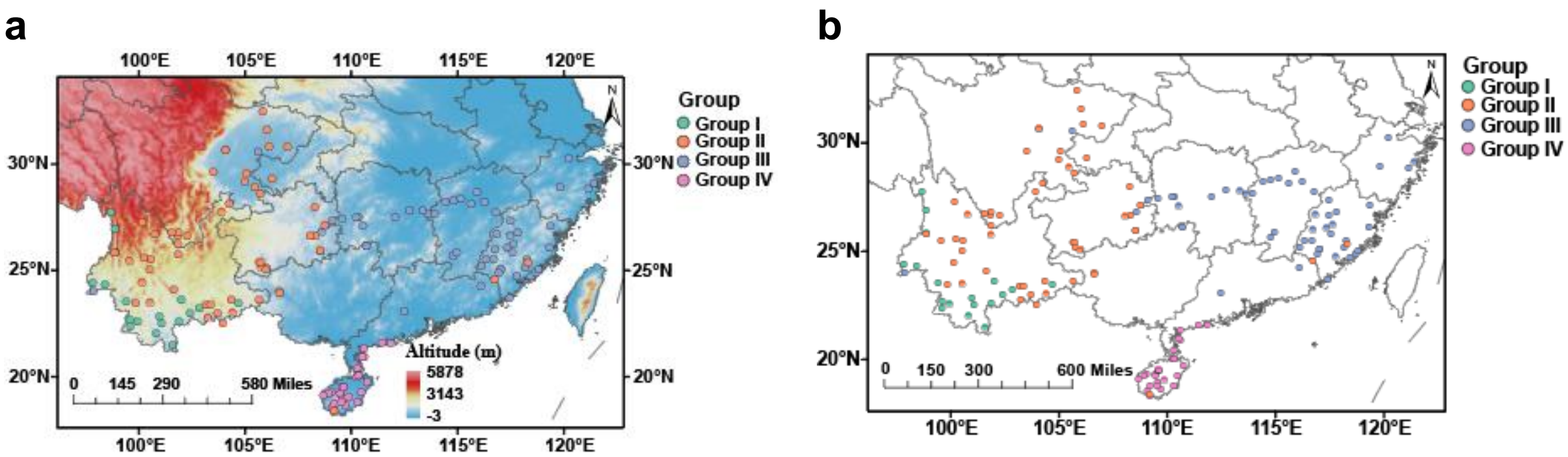

Response-Fig. 4 Original Figure 4c (a) and Revised Figure 4c (b). Geographic distribution of resequenced accessions. The map was generated using the mapping tool ArcGIS (version 10.8).

Reference:

1. Wang, T. *et al.* A complete gap-free diploid genome in Saccharum complex and the genomic footprints of evolution in the highly polyploid Saccharum genus. *Nat Plants* **9**, 554-571 (2023).
2. Zhang, Q. *et al.* Genomic insights into the recent chromosome reduction of autopolyploid sugarcane Saccharum spontaneum. *Nature Genetics* **54**, 885-896 (2022).
3. Zhang, J. *et al.* Allele-defined genome of the autopolyploid sugarcane *Saccharum spontaneum* L. *Nature Genetics* **50**, 1565-1573 (2018).
4. Wang, Y. *et al.* MCScanX: a toolkit for detection and evolutionary analysis of gene synteny and collinearity. *Nucleic Acids Res* **40**, e49 (2012).
5. Tang, H. *et al.* JCVI: A versatile toolkit for comparative genomics analysis. *iMeta* **3**, e211 (2024).
6. Zhang, J. *et al.* The highly allo-autopolyploid modern sugarcane genome and very recent allopolyploidization in Saccharum. *Nature Genetics* **57**, 242-253 (2025).
7. Healey, A.L. *et al.* The complex polyploid genome architecture of sugarcane. *Nature* **628**, 804-810 (2024).
8. Gabriel, L. *et al.* BRAKER3: Fully automated genome annotation using RNA-seq and protein evidence with GeneMark-ETP, AUGUSTUS and TSEBRA. *bioRxiv* (2024).
9. Brůna, T., Lomsadze, A. & Borodovsky, M. GeneMark-ETP significantly improves the accuracy of automatic annotation of large eukaryotic genomes. *Genome Res* **34**, 757-768 (2024).
10. Stanke, M., Steinkamp, R., Waack, S. & Morgenstern, B. AUGUSTUS: a web server for gene finding in eukaryotes. *Nucleic Acids Res* **32**, W309-12 (2004).

11. Li, A. *et al.* mRNA and Small RNA Transcriptomes Reveal Insights into Dynamic Homoeolog Regulation of Allopolyploid Heterosis in Nascent Hexaploid Wheat. *Plant Cell* **26**, 1878-1900 (2014).
12. Akhunova, A.R., Matniyazov, R.T., Liang, H. & Akhunov, E.D. Homoeolog-specific transcriptional bias in allopolyploid wheat. *BMC Genomics* **11**, 505 (2010).
13. Powell, J.J. *et al.* The defence-associated transcriptome of hexaploid wheat displays homoeolog expression and induction bias. *Plant Biotechnol J* **15**, 533-543 (2017).
14. Yoo, M.J., Szadkowski, E. & Wendel, J.F. Homoeolog expression bias and expression level dominance in allopolyploid cotton. *Heredity (Edinb)* **110**, 171-80 (2013).
15. Flagel, L., Udall, J., Nettleton, D. & Wendel, J. Duplicate gene expression in allopolyploid Gossypium reveals two temporally distinct phases of expression evolution. *BMC Biol* **6**, 16 (2008).
16. Zhang, T. *et al.* Sequencing of allotetraploid cotton (Gossypium hirsutum L. acc. TM-1) provides a resource for fiber improvement. *Nat Biotechnol* **33**, 531-7 (2015).
17. Sang, Y. *et al.* Genomic insights into local adaptation and future climate-induced vulnerability of a keystone forest tree in East Asia. *Nature Communications* **13**, 6541 (2022).
18. Liu, S. *et al.* Genome-wide analysis of *ZmDREB* genes and their association with natural variation in drought tolerance at seedling stage of *Zea mays* L. *PLoS Genetics* **9**, e1003790 (2013).
19. Matsukura, S. *et al.* Comprehensive analysis of rice DREB2-type genes that encode transcription factors involved in the expression of abiotic stress-responsive genes. *Molecular Genetics and Genomics* **283**, 185-96 (2010).
20. Li, Z. *et al.* Genome-wide identification and expression profiling of *DREB* genes in *Saccharum spontaneum*. *BMC Genomics* **22**, 456 (2021).
21. Mao, D. & Chen, C. Colinearity and similar expression pattern of rice DREB1s reveal their functional conservation in the cold-responsive pathway. *PLoS One* **7**, e47275 (2012).
22. Akbudak, M.A., Filiz, E. & Kontbay, K. DREB2 (dehydration-responsive element-binding protein 2) type transcription factor in sorghum (Sorghum bicolor): genome-wide identification, characterization and expression profiles under cadmium and salt stresses. *3 Biotech* **8**, 426 (2018).
23. Cingolani, P. *et al.* A program for annotating and predicting the effects of single nucleotide polymorphisms, SnpEff: SNPs in the genome of Drosophila melanogaster strain w1118; iso-2; iso-3. *Fly (Austin)* **6**, 80-92 (2012).
24. Vaser, R., Adusumalli, S., Leng, S.N., Sikic, M. & Ng, P.C. SIFT missense

predictions for genomes. *Nature Protocols* **11**, 1-9 (2016).

"Response to reviewers"

Reviewer #1 (Remarks to the Author):

While I do think that this revised manuscript is improved over the previous submission, I find the same issues (vague statements and lack of detail) persist throughout. Results are typically reported in relative terms (higher, lower) than absolutes with vague interpretations attached ("suggesting evolutionary expansion", " suggesting rapid evolution", etc..), and the discussion largely re-states the results. Below I include two examples where key details are missing from reported results:

Thank you for your feedback on our manuscript. We appreciate your guidance and have made the necessary modifications as per your recommendations:

1) **Use of Absolute Values and Clarity of Results**: We have replaced relative terms (higher, lower) with precise quantitative data or statistical measures (e.g., absolute values, *P* values) where applicable. For instance, the original statement: “Analysis of TEs distribution across chromosomes revealed that chromosome 08 (TraChr08) harbored a significantly higher TE density than other chromosomes.” has been revised to “Analysis of TEs distribution across chromosomes revealed that chromosome 08 (TraChr08) displayed the highest TE density, measured at 69.19%, which significantly exceeds the average density of 65.23% found on other chromosomes by 3.96 percentage points.” (Results, Lines 146-149).

2) **Elimination of Vague Interpretations:** We have strengthened vague phrasing in the manuscript, particularly replacing previous expressions with more specific and scientifically robust descriptions or removed some vague interpretations. For example, we have replaced “suggesting evolutionary genome expansion in *T. arundinaceum*” to more specific description “indicating that *T. arundinaceum* genome may have undergone expansion after its divergence from *Saccharum*” (Results, Lines 118-119).

3) **Increased Manuscriptal Details:** To clarify the experimental design and data

analysis further, we have supplemented the Methods section with detailed descriptions of the experimental conditions, sample processing, and data analysis techniques. Additionally, to concentrate on the most robust and well-supported aspects of our research, we have moved some peripheral or underdeveloped analyses into the Supplementary Notes, where they are only briefly summarized in the main text. This includes the detailed analyses of the *TraDREB* genes under drought stress. However, due to an oversight on our part, the corresponding Supplementary Note was previously not cited in the Results section, which prevented readers from accessing detailed descriptions of the relevant analyses. We have corrected this oversight and now appropriately cited the Supplementary Note in the relevant sections of the Results.

Enhancement of Discussion Depth: Enhanced Depth of Discussion: We also comprehensively revised the discussion to improve it and avoid merely restating results. For instance, we removed content that solely recapitulated findings, such as: "Meanwhile, the diploid last common ancestor of *S. spontaneum* and *T. arundinaceum* likely shared a similar geographical distribution, considering that *T. arundinaceum* and *S. spontaneum* experienced a shared bottleneck event during the Naynayxungla Glaciation, approximately 0.72 to 0.5 Mya (Fig. 5d).".

"Additionally, we discovered five TraDREB genes whose expression responded to drought stress, suggesting they may play important roles in drought tolerance (Supplementary Fig. 19). These findings provide a genetic foundation for the introgression of beneficial gene resources into sugarcane breeding." -There is no detail at all given here or in the methods about this analysis or result.

Response 1: Thank you for your valuable feedback. As outlined noted in Supplementary Note 5 (Lines 140-149 of Supplementary information), we have conducted a comprehensive analysis to identify these *TraDREB* genes and characterize their expression in response to drought stress. To enhance clarity, we have explicitly referenced Supplementary Note 5 in the Results section (Results, Line 214). Additionally, we have included detailed descriptions of drought treatment protocols and

sample collection methods in the "Plant Materials" section of the Methods (Methods, Lines 492-499). We also provide a thorough explanation of the methods used for the relative expression analyses of these *TraDREB* genes in the "Identification of Key Genes and Expression Profiling Analysis" section (Methods, Lines 650-661). We hope these modifications address your concerns and provide the necessary clarity regarding our findings.

"In our study, the candidate adaptive SNP located in the upstream region of CYP78A5 was significantly associated with BIO7 (Temperature Annual Range) (Fig. 6a, d). Allele frequency analysis exhibited that the C allele was predominantly found in regions with a high temperature annual range, while the T allele was nearly fixed in areas with low temperature (Fig. 6e)".- Where is the SNP relative to the gene? are they in LD? what are temperature ranges? These details should be included in the text, not found in figure captions.

Response 2: We appreciate the reviewer's constructive comments. We have now incorporated the following clarifications into the manuscript text:

1) SNP-Gene Relationship and LD Analysis: The candidate adaptive SNP is located within 2 kb upstream of *CYP78A5* (a cytochrome P450s protein). This upstream/downstream region definition was standardized using SnpEff v4.3t[1] with the parameter '-ud 2000' (Methods, Lines 693-695).

LD analysis revealed relatively high degree of LD (Supplementary Fig. 26a) between two candidate SNPs in the *CYP78A5* upstream region, both associated with BIO7 (Temperature Annual Range) (Fig. 6a, d) (Results, Lines 323-325). Similarly, a relatively high level of LD showed in the genomic region surrounding *bHLH112* (a basic helix-loop-helix transcription factor), which contains four candidate adaptive SNPs (Supplementary Fig. 26b) (Results, Lines 351-352). LD visualization was performed using LDBlockShow[2] (Methods, Lines 768-769).

2) Supplement Temperature/Precipitation Ranges: For temperature, The A allele frequency correlated with high BIO7 ranges (24.4-33.1 °C), while the T allele was fixed

in low-BIO7 regions (13.8-24.4 °C) (Fig. 6e) (Results, Lines 329-332). For precipitation, the A allele predominated in high-rainfall coldest quarters (162-290 mm), whereas the C allele was common in low-rainfall areas (39-162 mm) (Fig. 6g) (Results, 355-357).

Reviewer #2 (Remarks to the Author):

In the revised version of the manuscript the authors have significantly streamlined the manuscript and provided thorough responses to all of my concerns.

Thank you for the encouraging feedback and positive comments previously.

References:

1. Cingolani, P. *et al.* A program for annotating and predicting the effects of single nucleotide polymorphisms, SnpEff: SNPs in the genome of Drosophila melanogaster strain w1118; iso-2; iso-3. *Fly (Austin)* **6**, 80-92 (2012).
2. Dong, S.S. *et al.* LDBlockShow: a fast and convenient tool for visualizing linkage disequilibrium and haplotype blocks based on variant call format files. *Briefings in Bioinformatics* **22**(2020).

REVIEWER COMMENTS

Reviewer #1 (Remarks to the Author):

As noted from the previous revision, I appreciate the authors providing additional information and context for their results (providing absolute values, etc..) but while reading the document, i was almost immediately perplexed by the use of statistics and reporting. On line 117, the authors report a significant size difference among T. arundinaceum and Saccharum ($P < 0.01$, Wilcoxon rank sum test). But based on the numbers provided above (1.14 Gb, 0.79 Gb, etc..), there is not enough power to detect a statistical difference. What is the test statistic for this Wilcoxon test? Is the monoploid size for each of the subgenome chromosome copies being tested against combined subgenomes within the other genomes? Looking at Supplementary figure 4, that appears to be the case, as every pairwise combination of Saccharum species relative to T.a is significant (T.a vs LA purple, T.a vs FuGuo1). Regardless, there are no test statistics (W values, degrees of freedom) reported throughout the manuscript.

Response: We thank the reviewer for this valuable comment. Indeed, our initial statistical method (Wilcoxon rank sum test) was not ideal and lacked clarity. To resolve this problem, we have comprehensively revised our statistical approach to address the concerns regarding power, clarity, and reporting:

1) We have specified in the legend of Supplementary Figure 4 that the statistical comparisons are based on data points of monoploid subgenome size. Additionally, we have provided the W value of Wilcoxon rank sum test in the main text and the figure legend. When comparing three or more groups, we replaced the Wilcoxon rank-sum test with the Kruskal-Wallis test to assess overall significance, followed by Dunn's test for post-hoc analysis to identify specific group differences, thereby controlling for false positives.

2) We have reported all required test statistics throughout the manuscript and figures: The H-statistic, degrees of freedom (df), and *P*-value for the Kruskal-Wallis

test are provided in the main text and figure legends. The exact Z-statistics and adjusted *P*-values for all key pairwise comparisons from the Dunn's test are provided in the Source Data.

Again, the same spurious connections exist throughout the manuscript. Here is just one example:
Our comparative genomic analyses revealed that T. arundinaceum harbors a significantly higher ($P < 0.0001$, Wilcoxon rank sum test) burden of deleterious mutations than S. spontaneum. Moreover, genes carrying deleterious mutations in T. arundinaceum were primarily enriched in biological processes crucial for development and stress responses. These results imply that the accumulation of deleterious mutations may have compromised key adaptive pathways, potentially influencing phenotypic robustness and environment adaptability of T. arundinaceum.

1)There is no evidence to link expanded genome size to elevated mutational load.
2)Also, is there an expectation that phenotypic robustness and environmental adaptability are potentially compromised in T. arundinaceum? In the introduction and discussion, the authors argue that the species is useful for its 'exceptional adaptability'. Wouldnt the rationale be that the increased mutational load stems from the bottleneck events, and not a larger genome? And the unexpected result (exceptional adaptation + high mutational load) be worthy of discussion?

Response: Thank you for your insightful and profound review comments. The issues you raised regarding “spurious connections” are crucial. We re-examined the data and analysis methods and found that there was a calculation error when standardizing the number of deleterious mutations using CDS length. We have re-conducted the normalization analysis in this study.

1) Regarding the error correction and new findings: The corrected results showed that the deleterious mutation burden in *T. arundinaceum* is similar to that in *S. spontaneum*, and both are significantly lower than in *S. officinarum* (Kruskal-Wallis

test (H = 68.23, df = 3) with Dunn's post-hoc test, adjusted P < 0.0001) (Results, Lines 273-277). This new finding completely overturns our previous conclusion and elegantly resolves the contradiction you pointed out—namely, the unreasonable inference that *T. arundinaceum* has "exceptional adaptability" while carrying a high load of deleterious mutations. The data now indicate that the species with strong adaptability (*T. arundinaceum* and *S. spontaneum*) actually exhibit a lower burden of deleterious mutations, which is entirely consistent with evolutionary genetics theory (Discussion, Lines 442-448). We sincerely apologize for the errors in our previous analysis and the misleading conclusions that resulted, and we are truly grateful for your corrections, which have greatly enhanced the quality of our manuscript.

2) **Regarding the link between expanded genome size to elevated mutational load:** You are absolutely correct that our previous assertion lacked directed evidence. The corrected data indicate that there is no positive correlation between genome size and the load of deleterious mutations. Consequently, we have entirely removed this unsubstantiated causal link from the manuscript and have rewritten the Discussion section accordingly (Discussion, Lines 442-451).

Lastly, I looked for the evidence that supported the TraDREB genes playing a role in drought stress (a point I brought up before): From the manuscript:

Additionally, we discovered five TraDREB genes whose expression responded to drought stress, suggesting they may play important roles in drought tolerance (Supplementary Fig. 19; Supplementary Note 5). These findings provide a genetic foundation for the introgression of beneficial gene resources into sugarcane breeding.

Supp Note 5 reads: Quantitative real-time PCR analyses revealed that TraDREB1C, TraDREB1J, TraDREB2A, TraDREB2B, and TraDREB2C were up-regulated in response to drought stress (Supplementary Fig. 19).

Supp figure 19 shows the relative expression of the genes listed above at different time points (0h, 3h,6h, 9h) with the caption: Relative levels of gene expression of six

TraDREBs in the leaves of T. arundinaceum HN92-105 under drought conditions. Details are pretty sparse here. relative to what exactly? The zero timepoint? "CK" which is listed in the figure but not defined. Is 3 hours post nutrient removal considered drought stress? Regardless, this is not a demonstration of drought-upregulation, as there is no drought-free condition used as a control here. This expression pattern could just as easily be associated with circadian rhythm or something else entirely.

Response: We sincerely appreciate the reviewer's valuable and rigorous comments. In response to the concerns regarding the evidence for the role of *TraDREBs* in drought stress, we have made the following clarifications and modifications:

1) In our experiments, "CK" (control) refers to the sample collected before the initiating drought stress treatment (under normal condition, 0 hour), which we used as the baseline to calculate the relative expression levels at subsequent time points. To avoid ambiguity, we have explicitly added this definition in the caption of Supplementary Fig. 19 and the Method section (Methods, Lines 660-661).

2) As seen in studies on other grass species[1-3], a 3-hour post drought treatment interval is sufficient to induce significant expression changes in key drought-responsive genes, including *DREB* gene. However, we fully acknowledge that in the absence of direct physiological measurements (e.g., relative leaf water content) in our specific experiment for *T. arundinaceum*, referring to this state unequivocally as "drought stress" could be considered overreaching. To address this concern and ensure the utmost rigor, we have revised the relevant descriptions throughout the manuscript. Specifically, we changed "we discovered five *TraDREB* genes whose expression responded to drought stress, suggesting they may play important roles in drought tolerance" to "we discovered five *TraDREB* genes (*TraDREB1B*, *TraDREB1C*, *TraDREB1J*, *TraDREB2A*, and *TraDREB2C*), which are not circadian-regulated, showed significantly (T-test, $P < 0.05$) altered expression levels following drought treatment, suggesting their potential involvement in the drought stress response of *T. arundinaceum*" (Results, Lines 219-222), and the description of this analysis in Supplementary Note 5 has been updated accordingly (Supplementary Notes, Lines 141-

148, 157-158).

3) We fully agree with the reviewer that an ideal experimental design should include a "well-watered" control group throughout the entire experimental period. Refer to previous studies on short-term drought treatments[1-4], our study did not incorporate such a continuous control across all time points. To minimize potential confounding factors such as circadian rhythm, all samples were collected within a 9-hour window on the same day (Methods, Lines 651-661). Furthermore, we collected RNA-seq samples at 2-h intervals over a 24-h period from leaves under normal condition, and analyzed the diel expression patterns of *TraDREB* genes. The results showed that, except for *TrDREB2B*, none of the other genes exhibited circadian rhythm expression patterns (Response-Fig. 1). Therefore, we speculate that the upregulation of *TraDREB2B* under drought stress may be influenced by circadian rhythms rather than reflecting a specific role in the drought stress response of *T. arundinaceum* (Supplementary Notes, Lines 148-153).

4) This work is primarily a genomic study of *T. arundinaceum*. As noted in the Supplementary Notes, we have stated that our future functional validation on the role of *DREB* genes in the drought stress response of *T. arundinaceum* will include the following improvements: (1) a "well-watered" control group maintained throughout the entire experimental period, and (2) concurrent monitoring of physiological indicators correlated with drought severity (Supplementary Notes, Lines 158-162).

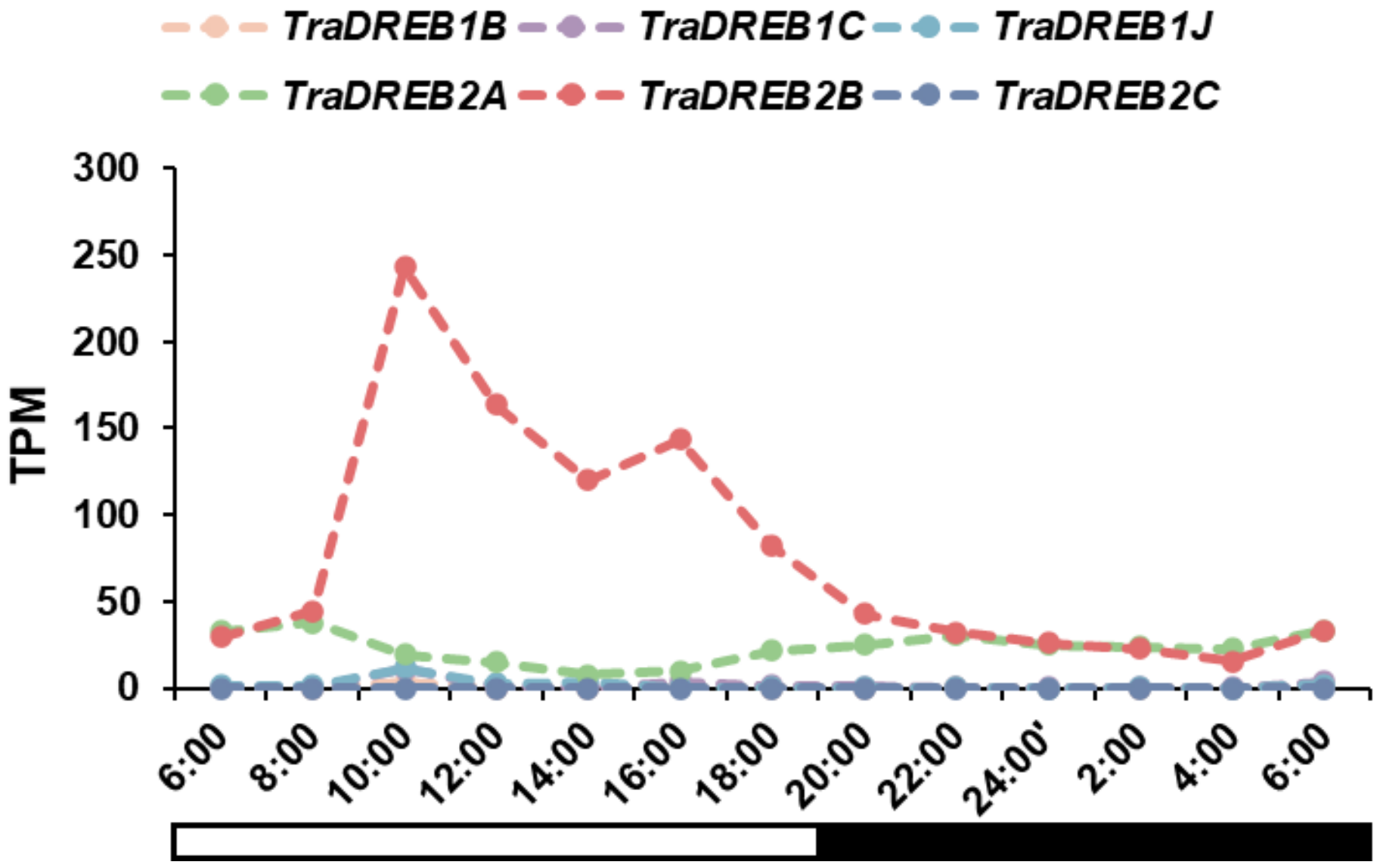

Response-Fig.1 Expression pattern of six *TraDREB* genes across the diurnal expression data. The x-axis represents time points at 2-hour intervals over a 24-hour period, and the y-axis represents the TPM (Transcripts Per Million) values of the genes.

Reference:

1. Mei, F. *et al.* A gain-of-function allele of a DREB transcription factor gene ameliorates drought tolerance in wheat. *Plant Cell* **34**, 4472-4494 (2022).
2. Luo, K. *et al.* The sugarcane ScMYB3R1-ScPYL61-ScPP2C57 module confers drought tolerance via ABA signaling. *Plant Physiology and Biochemistry* **228**, 110247 (2025).
3. Zhang, Q. *et al.* RIP5 Interacts with REL1 and Negatively Regulates Drought Tolerance in Rice. *Cells* **13**(2024).
4. Liu, S. *et al.* Genome-wide analysis of *ZmDREB* genes and their association with natural variation in drought tolerance at seedling stage of *Zea mays* L. *PLoS Genetics* **9**, e1003790 (2013).

REVIEWER COMMENTS

Reviewer #3 (Remarks to the Author):

For lines 117–121, the test is actually unnecessary here. Simply remove the word "significantly" and state that the genome is larger than theirs. Statistical tests are unnecessary in many instances, unless relevant conclusions are to be drawn based on their results. In my observation, the authors conducted numerous tests without deriving any corresponding conclusions from them.

Response 1: Thank you for your valuable feedback. We agree that the statistical test was unnecessary in this context, as the comparison was intended to present the factual difference in genome size estimates between these species, rather than to perform an inferential statistical analysis across biological populations. Following your suggestion, we have revised the sentence on original lines 117–121 to remove the word “significantly” and the associated statistical test description, and now simply state that the monoploid genome of *T. arundinaceum* is larger than those of the compared *Saccharum* accessions (revised manuscript, lines 116–119). The biological interpretation regarding potential genome expansion has been retained, as it is based on the observed size difference itself. Additionally, we have also revised the description of K_s value comparison among chromosomes and removed the associated statistical test description (revised manuscript, lines 184–187). Regarding the other statistical tests mentioned in our manuscript (e.g., comparisons of TE density, gene expression, and methylation levels across different chromosome groups), we believe they are necessary and appropriate. These tests are performed to infer whether observed differences (e.g., higher TE density on TraChr08, lower expression on PdCPs) are statistically supported across multiple biological units (chromosomes), rather than just stating simple factual comparisons.

Regarding the section on deleterious mutations, the authors compared different

populations and drew conclusions such as "both were significantly lower than that in S. officinarum". However, I consider these conclusions unreliable. Population data are scarcely comparable, as each population has its own unique evolutionary history and characteristics, making direct comparisons difficult. This is especially true for T. arundinaceum and sugarcane (S. officinarum), which diverged a long time ago—specifically, their split predates the divergence between sorghum and sugarcane. Therefore, such comparisons are meaningless. At most, the authors could have merely described the mutation rate of T. arundinaceum and similar metrics.

Response 2: We appreciate the reviewer's constructive comments. We agree with you about the limitations of cross-species population comparisons. Different species possess unique evolutionary histories and population characteristics, and direct comparisons of deleterious mutation loads can indeed be influenced by multiple factors, including effective population size, selection intensity, divergence time, etc. Our intention in performing this comparison was to explore observed differences in the patterns of deleterious mutation accumulation among sugarcane and its related species, rather than to assert any evolutionary superiority or inferiority.

Following your suggestions, we have revised the manuscript as follows:

1) The previous conclusive statement that "the deleterious mutation load in *T. arundinaceum* and *S. spontaneum* was significantly lower than that in *S. officinarum*" has been rephrased into a descriptive statement, emphasizing that these are numerical differences observed in this study (revised manuscript, lines 280–282).
2) We have added a statement regarding the limitations of cross-species comparisons, explicitly noting that the observed differences may be influenced by various factors: "However, we note that direct comparisons of deleterious mutation load across species should be interpreted with caution, as each species has undergone distinct evolutionary histories and demographic processes, and differences in genome assembly, annotation and population structure may also affect our estimates." (revised manuscript, lines 286–290).

3) We have removed the discussion section that was previously based on the direct cross-species comparison of deleterious mutation loads.

For lines 219–223, the authors state that the five TraDREB genes are not rhythm-related. However, the expression level of DREB2B is clearly much higher during the day than at night, and this finding is not described anywhere in the full text. In addition, the RT-PCR results of these five genes appear questionable: some are upregulated while others are downregulated, and their expression trends are inconsistent. The reliability of these results needs to be further verified, for example, by using RNA-seq data for cross-validation.

Response 3: We thank the reviewer for the careful evaluation of the *TraDREB* expression data and for these constructive comments. Our detailed responses are as follows:

1) **Concerning the diurnal expression pattern of *TraDREB2B*:** As the reviewer said that *TraDREB2B* is clearly much higher during the day than at night. We have described this expression pattern of *TraDREB2B* in the Supplementary Note 5 (Supplementary information, lines 151–153). In the revised manuscript, we now explicitly highlight this feature in the main text to avoid any misunderstanding. Specifically, we have added a brief sentence in the Results section to state that *TraDREB2B* exhibits a pronounced diurnal expression pattern under normal conditions (revised manuscript, lines 224–226). We have also clarified the original wording so that it is clear that the statement "not rhythm-related" refers to the other *TraDREB* genes, and does not include *TraDREB2B* (Supplementary information, lines 151–156).
2) **Reliability of RT-qPCR results and cross-validation with RNA-seq:** We fully appreciate the reviewer's concern regarding the reliability of the RT-qPCR results for *TraDREBs* under drought stress. Because the original drought-stressed samples are no longer available, we took advantage of an independent RNA-seq dataset generated from one-month-old *T. arundinaceum* seedlings subjected to osmotic

stress induced by 20% (w/v) PEG 6000. Polyethylene glycol (PEG 6000)–induced osmotic stress is widely used as a proxy for drought stress and has been shown to trigger highly comparable physiological and transcriptional responses to soil water-deficit–induced drought in various plant species.

Although the treatment durations and experimental setups of the RT-qPCR and PEG-RNA-seq experiments are not identical, the expression trajectories of the five *TraDREB* genes (excluding *TraDREB2C*, which showed no detectable expression under 20% PEG 6000 treatment) were largely concordant between the two datasets, in terms of the direction and overall trend of regulation relative to the control. This cross-validation supports the robustness of the RT-qPCR–based expression patterns. These comparative results have been added to the Supplementary Note5 (Supplementary information, lines 148-150; Supplementary Fig. 19).

In addition, we have revised the Methods section to explicitly state this cross-validation, as follows (revised manuscript, lines 673–676): “The expression patterns of *TraDREBs* measured by RT-qPCR were further supported by an independent transcriptome data from one-month-old *T. arundinaceum* seedlings subjected to drought stress with PEG 6000 (20%, w/v) and sampled at 0h, 4h, 8h, 16h, or 32h after treatment.”.

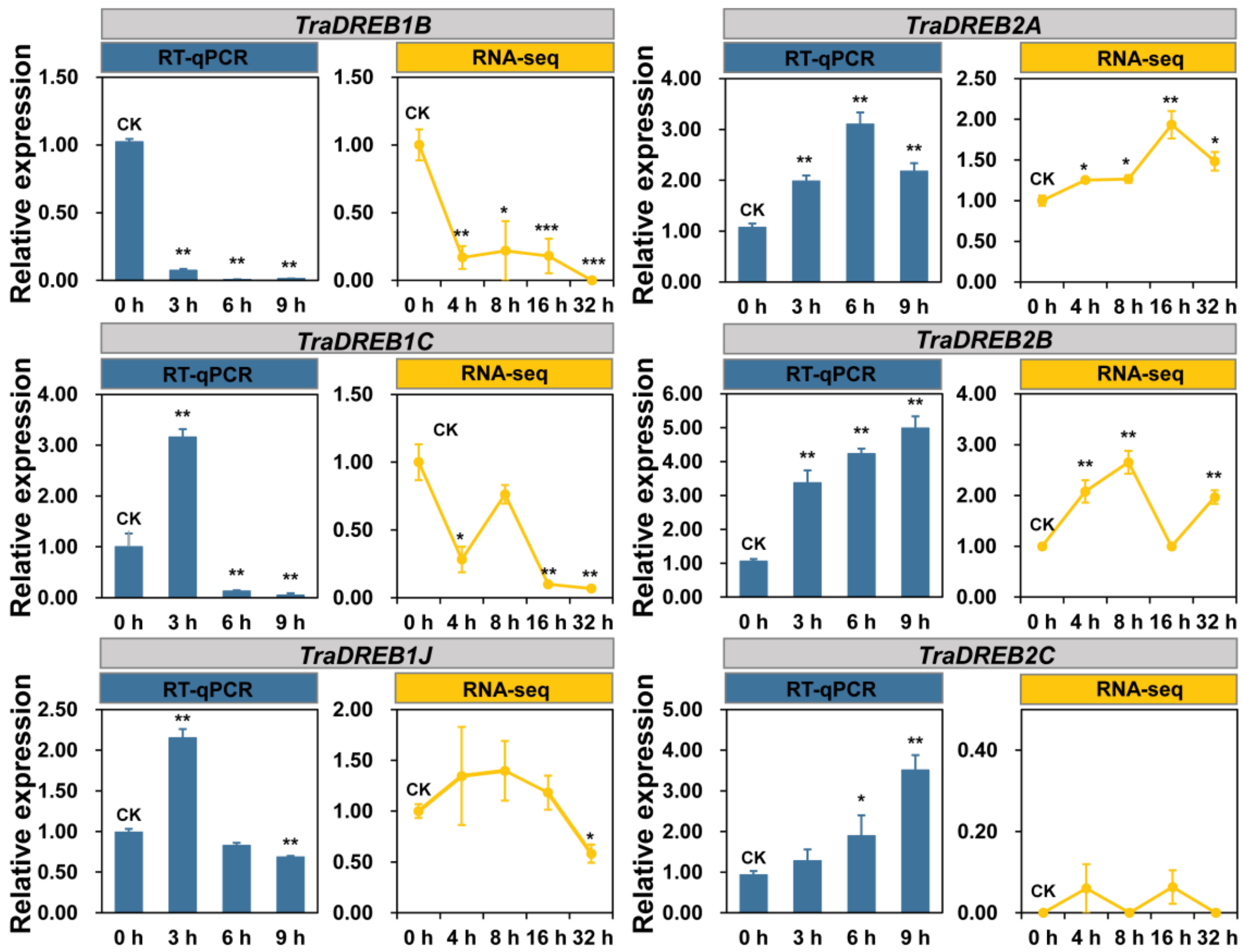

Supplementary Fig. 19 Relative expression levels of six *TraDREB* genes in *T. arundinaceum* HN92-105 under normal and drought conditions. The figure integrates results from two independent drought-stress experiments: RT-qPCR analysis of plants removed from the nutrient solution (left panels) and RNA-seq analysis of plants treated with 20% (w/v) PEG 6000 (right panels). RT-qPCR values (mean ± SD of three biological replicates) were normalized to the internal reference genes and calculated using the $2^{-\Delta\Delta Ct}$ method. RNA-seq expression levels are shown in Transcripts Per Million (TPM; mean ± SE, n = 3 biological replicates). Expression levels at all time points were normalized relative to the 0 h control sample (CK, collected before stress treatment). Asterisks denote statistically significant differences compared with the 0 h control (T-test, $*P < 0.05$, $**P < 0.01$).

There are several serious inaccuracies. For instance, the abstract mentions T. arundinaceum's origin in northern India and its close affinity to sorghum, both of which are incorrect. The presence of such flawed conclusions in the abstract indicates a lack of rigor in the authors' findings. Specifically, sorghum and sugarcane are sister groups, while T. arundinaceum is an outgroup to the sorghum-sugarcane clade. It is therefore inappropriate to claim that T. arundinaceum is closely related to sorghum; in fact, it shares an equal degree of genetic relatedness with both sorghum and sugarcane. Furthermore, Extended Data Fig. 2c fails to support the assertion that T.

arundinaceum originated in northern India. Notably, the genetic structure analysis in Extended Data Fig. 2a does not include any samples from northern India, making the conclusion about its northern Indian origin inexplicable.

Response 4: We are extremely grateful to the reviewer for identifying these serious inaccuracies in our manuscript and sincerely apologize for these errors. We have carefully considered each point and will implement the following revisions:

1) **Correction of Phylogenetic Relationship**: Based on the results of phylogenetic analysis and species divergence- time estimation, it is indeed inappropriate to directly state that "*T. arundinaceum* is phylogenetically closer to *S. bicolor* than to *Saccharum*", a more accurate description would be: "*Saccharum* is phylogenetically closer to *S. bicolor* than to *T. arundinaceum*". We have revised the entire manuscript accordingly.
2) **Re-evaluation of Geographic Origin Assertion**: Regarding the geographic origin hypothesis, we thank the reviewer for this important critique, which prompts us to clarify our reasoning. Our suggestion of a possible northern Indian origin for *T. arundinaceum* is based on a synthesis of inferences: Our demographic history analysis displayed a shared historical population event between *T. arundinaceum* and *S. spontaneum* (Fig. 5d); Both *T. arundinaceum* (Extended Data Fig. 2c) and *S. spontaneum*[1] have extant populations in northern India; Previous study showed that *S. spontaneum* is believed to have originated in northern India[1]. Therefore, we hypothesize that *T. arundinaceum* might also have originated in the same region. We acknowledge that this remains a speculation, and our sampling did not include northern Indian populations to test it directly. We have removed the conclusive statement "originated in northern India" from the abstract, and stated that pinpointing the exact origin requires further phylogeographic studies with comprehensive geographic sampling in Discussion section (revised manuscript, lines 449-451).

Reference:

1. Zhang, Q. *et al.* Genomic insights into the recent chromosome reduction of autopolyploid sugarcane Saccharum spontaneum. *Nature Genetics* **54**, 885-896 (2022).

Reviewer #3 (Remarks to the Author):

The previous response is well-written, yet there remain a few issues to address. Given the mention of Saccharum Complex in the title, the authors are presumably referring to sugarcane (the crop) rather than the genus Saccharum per se. Notably, Tripidium is even more distantly related to sugarcane than sorghum is, making it unrealistic to investigate sugarcane by means of Tripidium. Similarly, the article repeatedly claims that Tripidium holds significance for sugarcane breeding, which is a rather tenuous assertion. By contrast, Tripidium does have certain reference value for studying the evolution of plants in the genus Saccharum.

Accordingly, the article should explicitly state that the Tripidium genome is used to elucidate the evolution of the genus Saccharum, rather than claiming that it provides reference value for sugarcane research.

Response: Thank you for your feedback on our manuscript. We have carefully considered your comments and will revise the manuscript accordingly. Our point-by-point responses and planned revisions are as follows:

1) **Clarification of "*Saccharum* Complex":** The "*Saccharum* complex" is a natural gene pool of closely related, partially interbreeding sugarcane species and allied genera that have contributed to the origin and improvement of modern cultivated sugarcane; it mainly includes the domesticated high sugar canes (*Saccharum officinarum*), wild and hardy canes (*S. spontaneum*), and intermediate regional types (*S. barberi*, *S. sinense*), often extended to related genera such as *Erianthus*, *Miscanthus*, *Narenga*, *Sclerostachya*, and *Tripidium*, all of which can be used in breeding to combine high sugar yield with traits like disease resistance, stress tolerance and adaptability to diverse environments.

   We acknowledge that the term "*Saccharum* Complex" should accurately refer to the group of related genera involved in the origin of sugarcane, rather than simply the genus *Saccharum*. We have revised the manuscript to clearly state that this term encompasses the genera *Saccharum*, *Erianthus*, *Miscanthus*, *Narenga*,

Sclerostachya, and *Tripidium*, highlighting their potential for hybridization (revised manuscript, lines 55-58).

2) **The role of *T. arundinaceum* in sugarcane research:** We fully agree that *T. arundinaceum* is more distantly related to sugarcane than sorghum is. Originally, *T. arundinaceum* was classified within the genera *Saccharum*[1,2] or *Erianthus*[3,4]. Most recently, based on the whole chloroplast genome analysis, Lloyd Evans *et al.* reassigned *T. arundinaceum* to the genus *Tripidium*[5]. Sugarcane breeders have long recognized *T. arundinaceum* for its exceptional adaptability to abiotic stresses and other advantageous traits[6-9]. Consequently, for over a century, considerable efforts have been made to incorporate this species into sugarcane breeding programs[6,10].

In 1885, breeders first attempted hybridization between *T. arundinaceum* and sugarcane[6]. Since then, research on the hybridization of *T. arundinaceum* with sugarcane has been conducted in breeding programs in Barbados, Hawaii, Louisiana (USA), and South Africa. In China, the Hainan Sugarcane Breeding Station initiated a hybridization program between *S. officinarum* and *T. arundinaceum* in 1957 and over subsequent years obtained a limited number of F_1 hybrids that were identified as true hybrids based on isoenzyme and somatic chromosome number[11].

Recently twenty years, significant breakthroughs have been achieved in the hybridization breeding of *T. arundinaceum* and sugarcane[12]. Through multiple rounds of backcrossing (specifically referring to re-hybridization with elite varieties in sugarcane breeding practice), breeders have successfully introduced superior traits from *T. arundinaceum* (such as drought tolerance) into cultivated sugarcane germplasm[13,14]. For example, the advanced generation lines like BC_3F_1 derived from the cross between clones YC73-226 (*S. officinarum* × *S. spontaneum*) and YCE 06-111 (BC_3 between *S. officinarum* and *T. arundinaceum*)[15], which exhibit excellent physiological and stress-resistant traits (as demonstrated in studies by Liang *et al.*, Deng *et al.*)[16,17]. Additionally, clones YC96-66 and YC01-3 have been confirmed as genuine progenies of *S. officinarum* Badila and *T. arundinaceum* Hainan92-105 (the material used for genome assembly in this study)[18].

Consequently, we recognize *T. arundinaceum* as a valuable wild gene donor in sugarcane breeding. To further emphasize its importance as a genetic resource, we have incorporated relevant findings into the Introduction section of the revised manuscript (revised manuscript, lines 76-78).

Reference:

1. Clayton, W.D. & Renvoize, S.A. *Genera Graminum. Grasses of the World*, (Bernan-Unipub, 10033-F M. L. King Highway Lanham, MD 20706-4391, 1986).
2. Filgueiras, T. *Saccharum* L. in *Catalogue of New World Grasses (Poaceae): III. Subfamilies Panicoideae, Aristidoideae, Arundinoideae and Danthonioideae*, Vol. 46 (eds. FO, Z. *et al.*) 550-557 (Contributions from the United States National Herbarium, 2003).
3. Besse, P., McIntyre, C.L. & Berding, N. Characterisation of Erianthus sect. Ripidium and Saccharum germplasm (Andropogoneae - Saccharinae) using RFLP markers. *Euphytica* **93**, 283-292 (1997).
4. Besse, P. *et al.* Assessing genetic diversity in a sugarcane germplasm collection using an automated AFLP analysis. *Genetica* **104**, 143-153 (1998).
5. Lloyd Evans, D., Joshi, S.V. & Wang, J. Whole chloroplast genome and gene locus phylogenies reveal the taxonomic placement and relationship of Tripidium (Panicoideae: Andropogoneae) to sugarcane. *BMC Evolutionary Biology* **19**, 33 (2019).
6. Berding, N. & Roach, B.T. Chapter 4 - Germplasm Collection, Maintenance, and Use. in *Sugarcane Improvement through Breeding*, Vol. 11 (ed. Heinz, D.J.) 143-210 (Elsevier, 1987).
7. Jackson, P. & Henry, R.J. Erianthus. in *Wild Crop Relatives: Genomic and Breeding Resources: Industrial Crops* (ed. Kole, C.) 97-107 (Springer Berlin Heidelberg, Berlin, Heidelberg, 2011).
8. Amalraj, V., Rakkiyappan, P. & Devi, A.R. Evaluation of wild sugarcane *Erianthus arundinaceus* (Retz) Jesw. germplasm. *Journal of Sugarcane Research* **1**, 23-27 (2011).
9. Augustine, S.M., Syamaladevi, D.P., Premachandran, M., Ravichandran, V. & Subramonian, N. Physiological and molecular insights to drought responsiveness in *Erianthus* spp. *Sugar Tech* **17**, 121-129 (2015).
10. Peng, S. *Breeding of sugarcane*, (China Agriculture Press, Beijing, 1990).
11. Liao, Z.-z., Tang, M.-d. & Jin, Y.-f. Peroxidase Isozymes of Saccharum, Arundinaceum and Their Hybrids. *Acta Botania Sinica* **30**(1988).
12. Deng, H. *et al.* Breeding and isozyme marker assisted selection of F2 hybrids from Saccharum spp. X Erianthus arundinaceus. *Sugarcane Industry*, 1-5 (2022).
13. Augustine, S.M. *et al.* Erianthus arundinaceus HSP70 (EaHSP70)

overexpression increases drought and salinity tolerance in sugarcane (Saccharum spp. hybrid). *Plant Sci* **232**, 23-34 (2015).

14. Xiao, S. *et al.* Field evaluation of TaDREB2B-ectopic expression sugarcane (Saccharum spp. hybrid) for drought tolerance. *Frontiers in Plant Science* **13**, 963377 (2022).
15. Dou, Z., Deng, Q. & Shen, W. Evaluation of BC3F1 lines from intergeneric cross between Erianthus arundinaceus and Saccharum for resistance to sugarcane smut caused by Sporisorium scitamineum. *INTERNATIONAL JOURNAL OF AGRICULTURE & BIOLOGY* **19**, 1520–1524 (2017).
16. Deng, Q., Dou, Z., Chen, J., Wang, K. & Shen, W. Drought tolerance evaluation of intergeneric hybrids of BC3F1 lines of Saccharum officinarum × Erianthus arundinaceus. *Euphytica* **215**, 207 (2019).
17. Xie, J. *et al.* Analysis of Photosynthetic Characteristics in Distant Hybrids of Sugarcane and Erianthus arundinaceus across Different Generations. *Molecular Plant Breeding* **16**, 2723-2729 (2018).
18. Deng, Z.-h. *et al.* Analysis of Disequilibrium Hybridization in Hybrid and Backcross Progenies of Saccharum officinarum × Erianthus arundinaceus. *Agricultural Sciences in China* **9**, 1271-1277 (2010).

Reviewer #3 (Remarks to the Author):

Scientific research should first adhere to the principles of rigor, prudence, and reproducibility. Existing studies only suggest the possibility that *Tripidium arundinaceum* may be related to sugarcane or involved in introgression events with sugarcane, but there is no direct and conclusive experimental evidence to support this inference.

Although this study has assembled a high-quality genome of *Tripidium arundinaceum*, it has not demonstrated at the molecular or population level that genetic material from *Tripidium arundinaceum* has introgressed into sugarcane. Furthermore, the paper does not include any comparative analysis or validation experiments directly related to sugarcane.

Under such circumstances, the background and title of the article overemphasize sugarcane, which is inconsistent with the actual research content and is likely to mislead readers. This does not conform to the prudent attitude required in scientific research.

Response 1: Thank you for your critical and constructive comments, which have given us an important opportunity to clarify the scope, rationale, and rigor of our study. We have carefully considered each point and provide the following responses:

(1) Regarding direct evidence of gene introgression between *Tripidium arundinaceum* and sugarcane

We agree with the reviewer that this study does not provide direct molecular evidence of introgression from *Tripidium arundinaceum* into sugarcane, nor was it our intention to imply otherwise. *T. arundinaceum* has been used as a genetic resource in sugarcane breeding for more than a century and is widely regarded as one of the most important germplasm sources; several cultivars, including the Chinese varieties YT15491 (粤糖 15491) and YT15863 (粤糖 15863), are known to have *T. arundinaceum* in their pedigree. We briefly refer to oligo-FISH results for these two cultivars (personal communication with Dr. Jiayun Wu) to support this breeding history

(As shown in the figure below). With respect, we feel that a full re-presentation of those data would extend the scope of this manuscript beyond what is reasonable for a single paper, particularly given that it has already undergone six rounds of review.

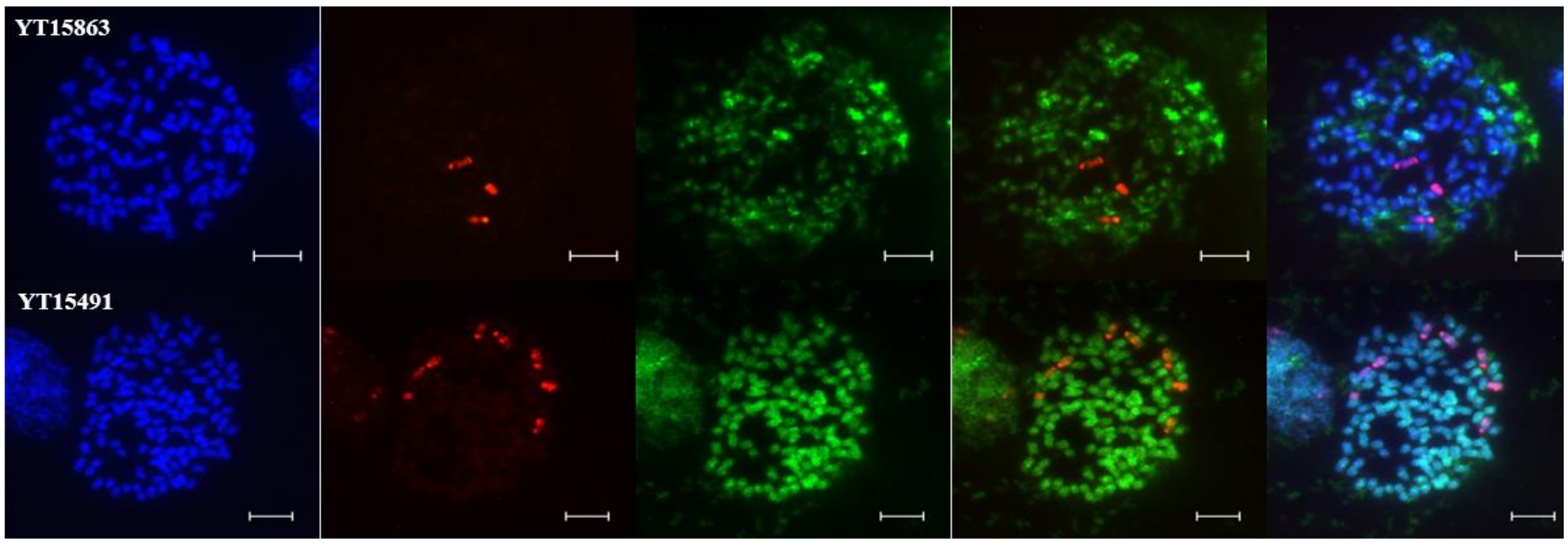

Figure. Chromosomal Detection of *T. arundinaceum* in Chinese varieties YT15491 (粤糖 15491) and YT15863 (粤糖 15863). *T. arundinaceum* chromosomes were visualized in red. YT15863 contains three *T. arundinaceum* chromosomes, and YT15491 contains eight *T. arundinaceum* chromosomes.

The primary objective of our work is to generate and analyze a high-quality reference genome for *T. arundinaceum*—a wild relative long recognized as a key breeding resource—and to investigate its evolutionary history and population structure within the *Saccharum* complex. We consider this genome to be a necessary foundation for future studies on introgression and the identification of beneficial alleles. Given the current early stage of genomic research in the *Saccharum* complex and the extreme complexity of the sugarcane genome, comprehensive, genome-wide introgression analysis is beyond the scope and technical feasibility of the present study.

Nonetheless, the extensive breeding literature cited in the Introduction and Discussion provides indirect biological and breeding evidence for the practical use of *T. arundinaceum* in sugarcane improvement. We fully agree that direct molecular evidence of introgression will be an important focus of future work, but we do not believe that the absence of such analyses diminishes the value of the genomic resource established here.

(2) Regarding Whether the Manuscript's Emphasis on Sugarcane is Misleading

T. arundinaceum has long been regarded by sugarcane breeders as one of the most

important wild germplasm resources. The starting point and ultimate goal of this study are to serve sugarcane breeding and research on the evolution of sugarcane relatives. Therefore, mentioning sugarcane in the background and title is not misleading, but rather an objective reflection of the research's applied prospects.

There are numerous other instances of inadequate rigor throughout the manuscript.
For example, the abstract claims that Population genomics analysis revealed Yunnan Province as a diversity center for T. arundinaceum in China. However, Group III and Group IV contain very few accessions from Yunnan.
In contrast, Group II includes several accessions from Hainan, and Group III shows substantial introgression from Hainan.
Notably, Hainan is located at the outermost position in the phylogeny, suggesting it may represent an earlier origin.

Response 2: We appreciate the reviewer's thoughtful comment, but we respectfully disagree with the interpretation that Hainan, rather than Yunnan, represents the diversity center or origin of *T. arundinaceum* in China.

(1) Definition of "diversity center"

In our manuscript, a "diversity center" is defined as a region that (i) harbours multiple, deeply diverged lineages and (ii) exhibits high within-population genetic diversity and rapid LD decay, rather than simply the province contributing accessions to the largest number of predefined groups.

Under this definition, southwestern China—particularly Yunnan—best fits a diversity center: (i) Accessions from Yunnan are distributed across three distinct genetic groups (Groups I–III) (Fig. 4a, c), indicating that this region contains several deeply diverged lineages of *T. arundinaceum*. (ii) Group II, which include many Yunnan accessions, exhibit the highest nucleotide diversity, and the fastest LD decay among all groups (Fig. 5a, b). These results support southwestern China/Yunnan as the main reservoir of genetic diversity for *T. arundinaceum*.

We agree that Groups III and IV each contain relatively few Yunnan accessions. However, our conclusion is not that "each group is centered in Yunnan", but rather that Yunnan is the only region where multiple, highly divergent lineages (Groups I–III) co-occur and where the most diverse groups are primarily distributed. This pattern is characteristic of a diversity center or long-term refugium, rather than of a peripheral or recently colonized region.

Southwestern China, including Yunnan and adjacent mountain ranges, has complex topography and heterogeneous climates and is widely recognized as a major Pleistocene refugium for many East Asian herbaceous and woody plants. This ecological and biogeographic context is consistent with *T. arundinaceum* persisting there over long time scales and subsequently expanding outward.

(2) Basal position of Hainan (Group IV) does not imply geographic origin

We also agree that the Hainan–Guangdong group (Group IV) occupies a basal position in the rooted phylogeny and appears as an early diverging lineage relative to the other *T. arundinaceum* groups (Fig. 4a). However, a basal or early-diverging lineage is not equivalent to a geographic center of origin. The tree topology suggests that Group IV diverged early from the common ancestor of the remaining groups, but it does not demonstrate that the species originated in Hainan.

Group IV is geographically restricted to the southern coastal/insular region (Hainan and Guangdong) and does **not** exhibit the highest nucleotide diversity or fastest LD decay (Fig. 5a, b). Instead, it represents an early-diverging, range-edge lineage with more limited diversity, consistent with long-term isolation on Hainan rather than a large ancestral source population. Thus, the basal position of Group IV reflects deep divergence and isolation at the southern margin of the species' range, not a higher ancestral diversity or a more likely origin.

(3) Introgression involving Hainan is consistent with a mainland diversity center

As the reviewer notes, Group II includes several accessions from Hainan and Group III shows signals of introgression from Hainan. We interpret this as evidence of secondary contact and gene flow between the Hainan lineage and mainland lineages,

rather than as proof that Hainan is the primary source. The fact that the highest nucleotide diversity and fastest LD decay are found in the predominantly mainland Groups II and III, rather than in Group IV itself, reinforces the interpretation that southwestern China functioned as the main reservoir of genetic diversity, with Hainan acting as a peripheral, occasionally introgressing lineage.

In summary, while Group IV (Hainan–Guangdong) is indeed an early-diverging lineage in the phylogeny, the combination of (i) multiple deeply diverged groups co-occurring in Yunnan (Groups I–III), (ii) the highest nucleotide diversity and fastest LD decay in Groups II and III, (iii) the geographically restricted, lower-diversity nature of Group IV, and (iv) the well-known role of southwestern China as a Pleistocene refugium supports our conclusion that southwestern China, centered on Yunnan, represents the diversity center—and likely a long-term refugium—of *T. arundinaceum*, rather than Hainan.

To avoid confusion, we will revise the Results to emphasize “diversity center in southwestern China (Yunnan)” and to clarify that the basal position of Group IV indicates early divergence, not necessarily a geographic origin. We believe these revisions will address the reviewer’s concern and improve the manuscript.

Given that sorghum is phylogenetically closer to sugarcane than T. arundinaceum,and given that the claim of introgression from T. arundinaceum into sugarcane remains highly questionable, I recommend that the authors remove all sugarcane-related background and content from the manuscript. In addition, the population genomic analyses of T. arundinaceum accessions should be completely re-analyzed, and all comparisons with sugarcane should be deleted.

Response 3: The reviewer notes that “Sorghum is phylogenetically closer to sugarcane than *T. arundinaceum*” and uses this to suggest removing sugarcane-related content. In our view, this comparison actually highlights the uniqueness and importance of studying *T. arundinaceum*: despite being more distantly related phylogenetically, it is one of the few close relatives that can successfully hybridize with sugarcane and produce backcross progeny in breeding programs.

Sorghum, while phylogenetically closer, has been far less successfully and directly utilized in sugarcane breeding. This is a classic example in biology where "phylogenetic relationship" does not always align with "breeding cross-compatibility," and it underscores the scientific and practical significance of our study. Historically, *T. arundinaceum* was thought to be more closely related to sugarcane than sorghum, whereas our results indicate that sorghum is in fact the closer relative at the genomic level. However, sorghum has not been widely used as a genetic resource in sugarcane breeding because most sorghum accessions are diploid, whereas *T. arundinaceum*—like cultivated sugarcane—is highly polyploid, making it more compatible for hybridization and introgression in practical breeding.

Completely removing all sugarcane-related content would sever this study from more than a century of sugarcane breeding practice and would leave readers wondering why this particular species was chosen for detailed genomic investigation.

Reviewer #4 (Remarks to the Author):

After evaluating the manuscript and the authors' responses to Reviewer #3, we provide our formal assessment. While the haplotype-resolved assembly of Tripidium arundinaceum represents a significant technical achievement, the current narrative framework contains major scientific inaccuracies and misleading interpretations that undermine the study's relevance.

Response: We thank the editor and reviewers for their thorough evaluation of our manuscript and our previous responses. We appreciate the recognition of the haplotype-resolved assembly of *Tripidium arundinaceum* as a significant technical achievement. At the same time, we fully acknowledge the concerns raised regarding scientific inaccuracies and potentially misleading interpretations in the current narrative framework. We take these comments very seriously and have carefully revised the manuscript to correct these issues and to ensure that the study's context, interpretations, and conclusions are fully aligned with current knowledge and appropriately reflect the relevance of our work.

We agree with Reviewer #3 that both the title and background overemphasize sugarcane and the concept of the Saccharum complex. This concept, first proposed by Mukherjee (1957), suggested that traditional sugarcane cultivars originated from interspecific hybridization among members of a putative interbreeding group comprising the genera Saccharum, Erianthus, Sclerostachya, and Narenga which he termed the 'Saccharum complex'. This group was later expanded by Daniels et al., 1975 to include Miscanthus. However, molecular data do not support the involvement of Erianthus/Tripidium or Miscanthus in the emergence of sugarcane cultivars and thus their contribution to sugarcane genome. Instead, molecular data indicate that modern sugarcane emerged from wild Saccharum species. Moreover, to our knowledge, no natural hybrids between T. arundinaceum and sugarcane have been reported and no sugarcane cultivars that are

grown commercially have been proven to incorporate chromosomes from T. arundinaceum. Due to the fact that some Tripidium arundinaceum have large, sugarcane-like stalks, there have been many attempts to use this species in sugarcane breeding. In fact, producing F1 and backcrosses between sugarcane and T. arundinaceum remains very difficult, and only a few of these events have been validated with molecular data. So, most breeding centers have abandoned this base broadening strategy and focus more on S. spontaneum.

In this context, the sentence of the abstract "Tripidium arundinaceum, a highly polyploid member of the Saccharum complex, has been used for over a century to broaden genetic diversity and enhance drought tolerance in sugarcane breeding" is inaccurate and misleading. The fact that Tripidium arundinaceum is phylogenetically quite distant from sugarcane, even more so than sorghum, could explain this lack of success in using Tripidium arundinaceum in sugarcane breeding.

This early concept of the Saccharum complex is misleading and should no longer be propagated.

Therefore, we agree that the background and title of the article overemphasize sugarcane and are misleading. We recommend removing the term "Saccharum complex" from the title and both the abstract and background should be modified to more accurately reflect current knowledge.

Response: We thank the reviewer for their insightful comments and fully agree that *T. arundinaceum* is phylogenetically distant from sugarcane, even more so than sorghum. We acknowledge that the traditional "*Saccharum* complex" concept, which historically included *T. arundinaceum*, is outdated and is not supported by the molecular evidence presented in our study. As noted by Lloyd Evans *et al.* (Lloyd Evans et al., 2019), the "*Saccharum* complex" should be restricted to species that can interbreed in the wild, namely *Miscanthus*, *Miscanthidium*, *Saccharum* and the New World *Erianthus* species. In China, only a few research groups have attempted to use *T. arundinaceum* in crosses with sugarcane to broaden the genetic background, and only a small number of sugarcane accessions derived from such crosses have been registered for commercial

cultivation; however, these accessions are grown on an extremely limited scale. Objectively, the breeding value of *T. arundinaceum* for sugarcane improvement is quite limited, and interspecific hybridization remains technically challenging.

In the revised manuscript, we have removed the term "*Saccharum* complex" from the title and have revised the abstract and background to more accurately reflect current knowledge, particularly our genomic findings. We have also carefully revised the Introduction and Discussion to de-emphasize the contribution of *T. arundinaceum* to sugarcane breeding and instead to emphasize its significance as an evolutionarily important wild grass, highlighting its genomic features, ecological adaptability, and independent value as a genetic resource. (All modifications are highlighted in red font)

Regarding the claim that southwestern China (Yunnan) represents a "diversity center," the data presented in Figure 4 are difficult to interpret. A first difficulty arises from the fact that the admixture analysis (Figure 4b) includes only a subset of the accessions present in the phylogeny (Figure 4a). Furthermore, the identified group, except for group1, consist of both pure and hybrid accessions which are represented by dots with the same color in Figure 4c (map). So, the map fails to show how these pure and hybrid lineages are actually distributed. This ambiguity extends to the Principal Component Analysis (Figure 4d), which also appears to include only a fraction of the total samples (presumably only those considered "pure»?) further obscuring the global diversity picture. So, these figures should be modified.

Response: We thank the reviewer for the careful reading and for pointing out the ambiguities in Figure 4. We apologize for any confusion caused by the original presentation and have revised the figure and the corresponding text accordingly. Please find our point-by-point response below:

1) **Regarding the claim that Figure 4b includes only a subset of accessions present in Figure 4a:** We guarantee that all analyses presented in Figure 4 are based on the same complete set of 153 wild *T. arundinaceum* accessions collected from China.

In the original version, we did not display all sample names in Figure 4b because showing all would have made the admixture plot excessively large and difficult to integrate into the multi-panel figure. We apologize for this omission, which led to the misunderstanding. To avoid this issue, we have now removed all sample names from Figure 4b entirely, as they are not essential for interpreting the population structure at this scale.

2) **Regarding the map (Figure 4c) and the distinction between "pure" and "hybrid" accessions:** We acknowledge the reviewer's concern about the use of the same color for both pure and mixed accessions within the same group. However, we wish to clarify that all materials used in this study are wild, naturally occurring *T. arundinaceum* populations. There are no artificially pure lines or controlled hybrid lineages. In the admixture analysis, Groups II, III, and IV show mixed ancestry components from other groups, suggesting historical gene flow or introgression. In contrast, Group I shows no such admixture from other groups, suggesting it may represent the most ancestral *T. arundinaceum* group in China.
3) **Regarding Figure 4d (PCA) and the concern that only a subset of samples is shown:** We guarantee that the PCA was also performed using all 153 accessions. The reviewer's impression that only a subset was shown likely arose from the presence of colored dots in the original Figure 4a, which represented different provinces rather than the subpopulations used in Figures 4c and 4d. This inconsistency has been corrected: we have removed the colored dots from Figure 4a to avoid further misinterpretation (Revised Figure 4).

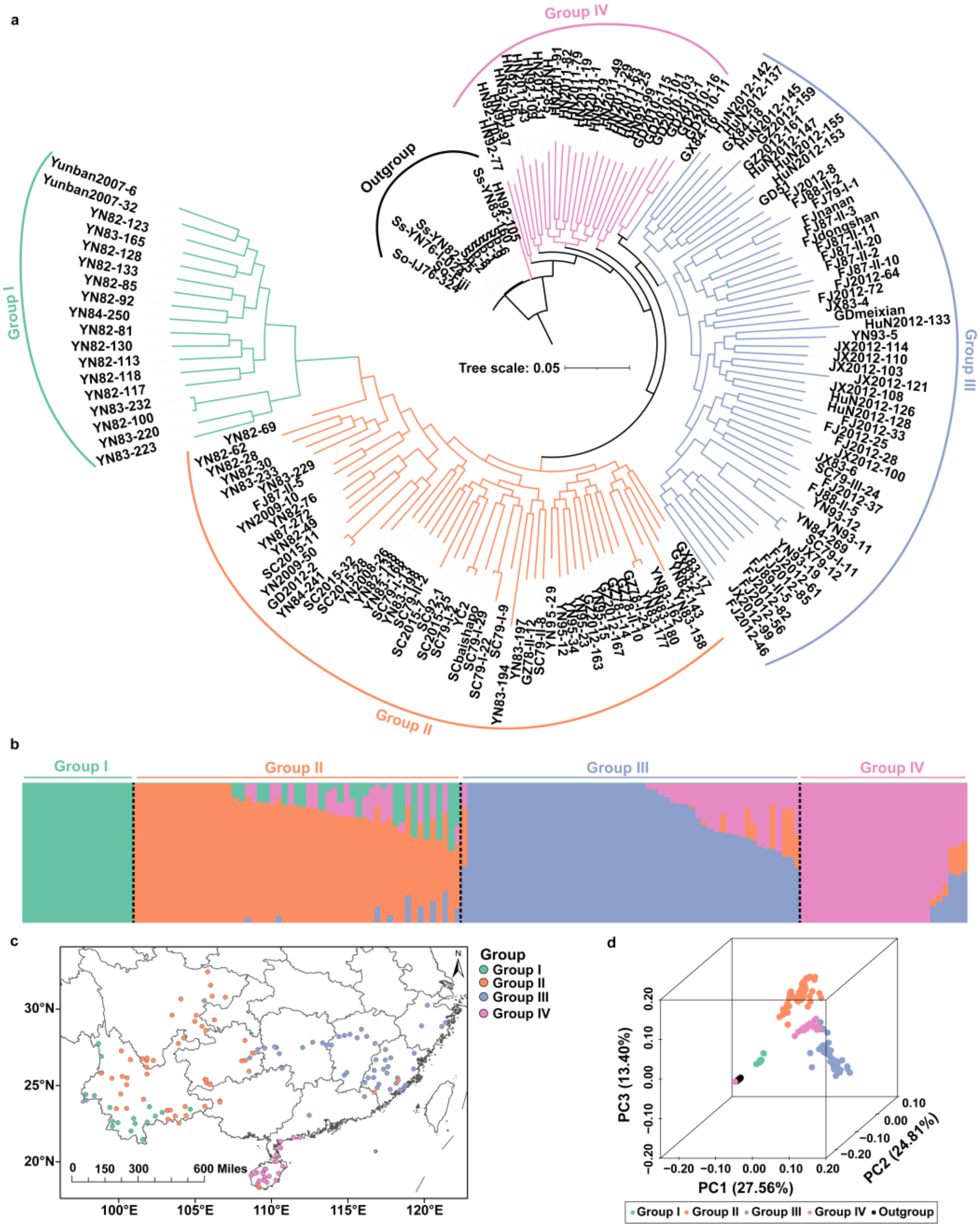

Revised Figure 4 Phylogenetic relationship of 153 *T. arundinaceum* accessions. a Maximum likelihood phylogenetic tree of *T. arundinaceum* accessions. Ss, *S. spontaneum*; So, *S. officinarum*; Sb, *S. bicolor*. **b** Population structure analysis of representative *T. arundinaceum* accessions at K = 4. **c** Geographic distribution of re-sequenced accessions. The map was generated using ArcGIS (version 10.8). **d** PCA plots of the first three principal components. PC1, PC2 and PC3 explain the largest proportions of variance, respectively. Colored dots indicate different subpopulations of *T. arundinaceum* accessions, and black dots represent the outgroup accessions.

In addition, the natural distribution of *T. arundinaceum* extends continuously across Southeast Asia and into northeastern India. Without including samples from this broader distribution, the observed diversity in Yunnan may simply reflect the edge of a

wider genetic gradient rather than a localized diversification hotspot.

So, we agree with reviewer 3, with the current presented data the claim of a "diversity center," is not sufficiently supported.

Response: We thank the reviewer for this important comment. We fully agree that the natural distribution of *T. arundinaceum* extends continuously across Southeast Asia and into northeastern India. Without adequate sampling from these regions, our current data cannot support the claim that Yunnan represents a global or species-wide diversity center for *T. arundinaceum*. However, in this study, we restricted our analyses to Chinese populations of *T. arundinaceum*. Within this defined geographic scope, our results consistently show that southwestern China (Yunnan) harbors the highest genetic diversity among all sampled Chinese populations. Accordingly, we have carefully qualified our conclusion to state that southwestern China (Yunnan) may serve as "**a center of genetic diversity for *T. arundinaceum* within China**" (see revised manuscript, lines 35–36 and 434–440). (Revised manuscript, **lines 35-36:** Population genomics analysis revealed southwestern China (Yunnan) as a diversity center for *T. arundinaceum* within China; **lines 434-440:** Notably, three distinct subpopulations (Group I-III) were identified in Yunnan Province. Groups I and II consisted predominantly of accessions collected from Yunnan (Fig. 4a, c), with Group II exhibiting the highest nucleotide diversity and the fastest LD decay (Fig. 5a, b), indicating pronounced intra-specific diversity within southwestern China (Yunnan). These observations corroborate previous findings by Cai *et al*.(Cai et al., 2012), supporting the hypothesis that southwestern China (Yunnan) may serves as a center of genetic diversity for *T. arundinaceum* within China.).

There are several other weaknesses in the studies in particular we have reservations regarding some of the population genomic methodologies used, specifically concerning the tools and parameters used to account for the polyploid nature of T. arundinaceum.

Response: We thank the reviewer for this valuable comment. We fully acknowledge the current limitations in population genomic methodologies specifically tailored and rigorously validated for highly polyploid species such as *T. arundinaceum*. In practice, many population genomic studies of polyploids continue to rely on diploid-oriented pipelines as a pragmatic approximation. For example, in recent work on allo-autopolyploid sugarcane (Zhang et al., 2025) and allopolyploid *Erianthus rockii* (Wang et al., 2025), variant calling was performed using the Sentieon DNAseq pipeline under default diploid settings. In our study, we adopted a similar strategy for variant identification in the *T. arundinaceum* population, in line with these precedents.

We believe that graph-based genome approaches and more specialized tools may ultimately provide a better framework for modelling the polyploid nature of *T. arundinaceum* (Huang et al., 2026). However, given the current scarcity of genomic resources and dedicated methods for this species, such analyses remain technically challenging at this stage.

We respectfully hope that the reviewer will find this clarification and revision acceptable, and we are very grateful for the constructive suggestions.

Reference:

Cai, Q., Aitken, K.S., Fan, Y.H., Piperidis, G., Liu, X.L., McIntyre, C.L., Huang, X.Q., and Jackson, P. (2012). Assessment of the genetic diversity in a collection of Erianthus arundinaceus. Genetic Resources and Crop Evolution **59,** 1483-1491.

Huang, Y., Zhang, Y., Zhang, Q., Zhuang, G., Li, C., Wang, B., Gao, R., Xu, Y., Qi, Y., Hua, X., Shi, H., Xu, Q., Yao, W., Liu, X., Qi, Y., Chen, B., Zhang, M., Ming, R., Tang, H., and Zhang, J. (2026). Multiscale pangenome graphs empower the genomic dissection of mixed-ploidy sugarcane species. Science (New York, N.Y.) **391,** eadx1616.

Lloyd Evans, D., Joshi, S.V., and Wang, J. (2019). Whole chloroplast genome and gene locus phylogenies reveal the taxonomic placement and relationship of Tripidium (Panicoideae: Andropogoneae) to sugarcane. BMC evolutionary biology **19,** 33.

Wang, B., Zhang, Z., Qi, Y., Wei, Y., Mei, J., Liu, H., Wang, Y., Fang, Y., Hua, X., Ding, H., Zhang, Y., Feng, X., Huang, Y., Li, Z., Chang, H., Wang, Q., Lin, X., Liu, X., Deng, Z., Yao, W., Tang, H., and Zhang, J. (2025). Near-complete genome assembly of allotetraploid Erianthus rockii reveals unique chromosome evolution and lineage-divergence trajectories in the Saccharum complex. Plant Commun **6,** 101464.

Zhang, J., Qi, Y., Hua, X., Wang, Y., Wang, B., Qi, Y., Huang, Y., Yu, Z., Gao, R., Zhang, Y., Wang, T., Wang, Y., Mei, J., Zhang, Q., Wang, G., Pan, H., Li, Z., Li, S., Liu, J., Qi, N., Feng, X., Wu, M., Chen, S., Du, C., Li, Y., Xu, Y., Fang, Y., Ma, P., Li, Q., Sun, Y., Feng, X., Yao, W., Zhang, M., Chen, B., Liu, X., Ming, R., Wang, J., Deng, Z., and Tang, H. (2025). The highly allo-autopolyploid modern sugarcane genome and very recent allopolyploidization in Saccharum. Nature Genetics **57,** 242-253.

REVIEWERS' COMMENTS

Reviewer #4 (Remarks to the Author):

Dear Editor,

We appreciate the authors' constructive responses to our previous comments, as well as the revisions made to the manuscript. To ensure complete consistency and accuracy before publication, we request the following final adjustments:

Abstract (line 26): Please replace the term "employed" with "tested" to avoid any misunderstanding and the very limited success in the use of Tripidium arundinaceum in commercial sugarcane improvement.

Response: Thank the reviewer for the valuable comments. As requested, we have revised the word "employed" to "tested" in the abstract to avoid potential misunderstanding (Revised manuscript, line 29).

Introduction (line 50) and Discussion (line 394): Please cite Welker et al. (2019; DOI: 10.1002/tax.12030) alongside the currently cited Lloyd Evans et al. (2019), as both groups independently reassigned Erianthus arundinaceus to Tripidium in the same year.

Response: We thank the reviewer for the suggestion. As requested, we have added the citation of Welker *et al.*[1] in both the Introduction and the Discussion (Revised manuscript, lines52-53, 394-395).

(lines 71–72): The statement that "only very few commercially grown sugarcane cultivars have been conclusively shown to carry chromosomes derived from T.arundinaceum" must be supported by a specific reference.

Response: We thank the reviewer for this comment. We agree that this statement requires supporting references. However, among the commercially sugarcane cultivars containing *T. arundinaceum* germplasm listed in the official variety registration database (http://202.127.42.47:6010/index.aspx), none are associated with published articles. For example, YueTang15491 and YueTang15863, which carry three and eight *T. arundinaceum* chromosomes, respectively, have completed variety registration but have no formal publications. We have cited these two sugarcane varieties as examples in the revised manuscript (Revised manuscript, line 73).

(line 404): The sentence stating that "E. rufipilus considered the last common ancestor of Saccharum" is not clear: last common ancestor between which taxons? and scientifically inaccurate. E. rufipilus (= E. fulvus) and Saccharum diverged from a common ancestor approximately 2–2.5 Mya (Wang et al. 2023).

Response: We thank the reviewer for this correction. We acknowledge that the original statement describing "*E. rufipilus* considered as the last common ancestor of *Saccharum*" was indeed imprecise and scientifically inaccurate. We have revised the relevant statement in accordance with the reviewer's suggestion (Revised manuscript, lines 405-407).

Figure 4b: Although the clarifications have improved the interpretation of the population genomics data, the names of the accessions must be identifiable in the admixture bar plot (Figure 4). If the image resolution does not allow for this, a Supplementary Table listing the exact order of the samples as they appear in the plot must be provided and referenced in the text where Figure 4 is discussed.

Response: We thank the reviewer for this valuable suggestion. We have compiled the names of all accessions into a supplementary table (Supplementary Data 4), arranged in the actual order of samples as presented in the mixed bar chart of Figure 4b. A corresponding citation has been added in the text where Figure 4b is discussed (Revised manuscript, lines 248).

Reference:

1. Welker, C.A.D., McKain, M.R., Vorontsova, M.S., Peichoto, M.C. & Kellogg, E.A. Plastome phylogenomics of sugarcane and relatives confirms the segregation of the genus Tripidium (Poaceae: Andropogoneae). *Taxon* **68**, 246-267 (2019).
