# Supplementary material for: Haplotype-resolved genome assembly sheds light on the evolutionary history of autohexaploid Tripidium arundinaceum
Source: Nat Commun. 2026 Jun 27;17:8348. doi: 10.1038/s41467-026-74742-1 (PMC13473609; doi:10.1038/s41467-026-74742-1)
Supplement: Supplementary file 3 [file 41467_2026_74742_MOESM3_ESM.pdf]

**Description of Additional Supplementary Files**

File Name: Supplementary Data 1
Description: Statistics of genome coverage of identified transposable elements on each chromosome of *T. arundinaceum*.

File Name: Supplementary Data 2
Description: The information of DREB genes in the *T. arundinaceum* Hainan92-105 genome.

File Name: Supplementary Data 3
Description: The detailed information for all resequencing accessions.

File Name: Supplementary Data 4
Description: Accession names and their corresponding groups in the admixture bar plot of Figure 4b.

File Name: Supplementary Data 5
Description: The environmental variables with variance inflation factors (VIFs) < 10 for 153 *T. arundinaceum* accessions.

File Name: Supplementary Data 6
Description: Detailed information of environmental associated variants identified in this study.

File Name: Supplementary Data 7
Description: Gene Ontology (GO) analysis of genes affected by variants of *T. arundinaceum* population.

File Name: Supplementary Data 8
Description: Summary of functionally important genes associated with detected environmental-associated variants.
