# Supplementary material for: Haplotype-resolved genome assembly sheds light on the evolutionary history of autohexaploid Tripidium arundinaceum
Source: Nat Commun. 2026 Jun 27;17:8348. doi: 10.1038/s41467-026-74742-1 (PMC13473609; doi:10.1038/s41467-026-74742-1)
Supplement: Supplementary file 4 [file 41467_2026_74742_MOESM1_ESM.docx]

**Haplotype-resolved genome assembly sheds light on the evolutionary history of** **autohexaploid *Tripidium arundinaceum***

Li *et al.*

**Supplementary Note 1. Genome quality assessment**

The assembled genome exhibits a highly quality value (QV) of 71.20, with the mapping rates of Hi-C, Illumina and HiFi reads were over 98.41%, 98.03%, and 99.97%, respectively (Supplementary Table 2 and 4). Benchmarking Universal Single-Copy Orthologs (BUSCO)^1^ and compleasm^2^ revealed 98.80% and 99.69% completeness among 1,614 conserved gene groups, respectively (Supplementary Table 5). Additionally, the Hi-C interaction matrix revealed prominent diagonal blocks, reflecting higher contact frequencies between proximal genomic regions than distal ones, further supporting proper chromosomal scaffolding (Supplementary Fig. 2). Collectively, these findings confirm the high quality, continuity, and completeness of the genome assembly for *T. arundinaceum* Hainan92-105.

**Supplementary Note 2. Allelic expression dominance**

Global expression levels of abundantly expressed genes among each HG were similar in the examined tissues of *T. arundinaceum* Hainan92-105 (Supplementary Fig. 6). This similarity indicates that homologous chromosomes do not exhibit dominant expression patterns, likely due to the exchange of homologous chromosomes across generations. To analyze the allelic expression imbalance, we ranked the expression levels of six alleles for 16,047 genes in descending order (designated as I to VI). Differential expression analysis was performed on five consecutive allele pairs (I/II, II/III, III/IV, IV/V and V/VI). An allelic group on homologous chromosomes was defined as non-neutral if any pair of alleles exhibited more than two-fold difference in expression, otherwise, the group was considered as neutral. Among these genes, an average of 70.31% exhibited neutral expression, while 29.69% showed non-neutral expression (Supplementary Table 9), differing from the results in *S. spontaneum*, which had a higher proportion of non-neutral expression alleles^3^. On average, 2,678 (28.23%) neutral and 676 (11.12%) non-neutral genes were conserved across all tissues (Supplementary Fig. 7). Gene ontology (GO) enrichment analysis showed that these conserved non-neutral genes were enriched in categories such as ‘negative regulation of biological process’, ‘negative regulation of response to stimulus’, ‘protein dephosphorylation’, ‘regulation of developmental process’, ‘phosphoprotein phosphatase activity’, and other biological processes (Supplementary Fig. 8). This finding suggest that these allele-specific expression genes may play important role the process of defense and developmental in *T. arundinaceum*.

**Supplementary Note 3. DNA methylation in the genome of *T. arundinaceum***

Epigenetic modification by DNA methylation is conserved and essential for gene regulation and genome stability^4^. We further compared the methylation patterns between paleo-duplicated chromosome pairs (PdCPs) and other chromosomes in *T. arundinaceum*. The CG and CHG methylation levels showed a similar trend, with higher proportions on TraChr05 (79.46% for CG and 49.66% for CHG) and TraChr08 (79.84% for CG and 48.65% for CHG) relative to other chromosomes (76.05% for CG and 47.60% for CHG). In contrast, the CHH methylation level on TraChr05 (2.01%) was significantly lower (Kruskal-Wallis test (*H* = 11.96, df = 2) with Dunn’s post-hoc test) than that on TraChr08 (2.31%) (adjusted *P* = 2.69 × 10^-03^) and other chromosomes (2.29%) (adjusted *P* = 4.24 × 10^-03^) (Supplementary Fig. 10d). Moreover, we found that TEs regions exhibited high levels of methylation, with approximately 90.41%, 57.32%, and 2.69% of methylated cytosines were distributed in the CG, CHG, and CHH context, respectively (Supplementary Figs. 10e and 11). Additionally, we observed a strong negative correlation between the CG, CHG methylation levels and transcription abundances around transposable elements (TEs) (*P* < 2.2 × 10^-16^, Spearman’s correlation; Supplementary Fig. 12). Collectively, these findings implicate TEs in the suppression of adjacent genes, likely mediated by localized increases in DNA methylation.

**Supplementary Note 4. Analysis of** **gene family expansion and contraction**

Analysis of orthologous genes in the genomes of *T. arundinaceum* and nine related species revealed that a total of 519,821 genes were clustered into 37,706 orthologous gene families, with 7,607 gene families shared by all ten species (Supplementary Fig. 9c). Additionally, we employed the Computational Analysis of Gene Family Evolution (CAFÉ5)^5^ software to investigate changes in gene family size during evolution. A total of 4,867 gene families were identified as having undergone rapid changes at the *T. arundinaceum* node, comprising 388 expansions and 4,479 contractions (Supplementary Fig. 9b). The expanded genes were significantly enriched in KEGG pathway associated with Isoflavonoid biosynthesis, Phenylpropanoid biosynthesis, Ubiquitin system, Glycosyltransferases and Cytochrome P450 (*P* < 0.05, Fisher’s exact test) (Supplementary Fig. 9d), suggesting that the rapidly expanding genes may be linked to the stress resistance of *T. arundinaceum.*

**Supplementary Note 5. Genes related to key agronomic characteristics**

In this study, we identified 42 genes related to sugar metabolism, including sucrose phosphate synthases (SPS), sucrose synthases (SUS), invertases (INV), and fructokinases (FRK) (Supplementary Fig. 13). Compared to *S. spontaneum* Np-X, the number of *SPS*, *SUS*, and *FRK* have contracted in *T. arundinaceum*, while *INV* genes have expanded (Supplementary Fig. 13). Additionally, a total of 115 putative genes of sugar transporter superfamilies were found in the genome of *T. arundinaceum*, with a greater number of members in SWEET (Sugars Will Eventually be Exported Transporters), PLT (Polyol transporter), and STP (sugar transporter protein) families compared to *S. bicolor* and *S. officinarum* LA-purple (Supplementary Fig. 13), indicating that these three families experienced gene expansion in *T. arundinaceum* following its divergence from *S. bicolor*. In monocots, the cellulose synthase (CesA) family and cellulose synthase-like (Csl) family members are generally believed to medicate the synthesis of cellulose and the hemicellulose polysaccharides, respectively^6-9^. In *T. arundinaceum*, we identified 17 *CesA* genes and 30 *Csl* genes (including 9 *CslAs*, 5 *CslCs*, 5 *CslDs*, 2 *CslE**s*, 7 *CslFs*, and 2 *CslHs*) (Supplementary Fig. 13). Comparative analysis with *S. bicolor* and *Saccharum* revealed that *CesA* gene families has expanded in the genome of *T. arundinaceum*, providing a genetic basis for its high biomass.

*T. arundinaceum*, once regarded as an important germplasm resource for sugarcane breeding, relevant studies on the C_4_ pathway have not yet been reported. In this study, we conducted preliminary quantification of the photosynthetic physiological characteristics of *T. arundinaceum* and *S.* hybrid, revealing that the net photosynthetic rate and A-Ci response curve of *T. arundinaceum* was similar to that of sugarcane (Supplementary Fig. 14a, Supplementary Table 12), preliminary suggesting that *T. arundinaceum* is a C_4_ plant*.* To further characterize its photosynthetic pathway, we established a leaf developmental gradient for *T. arundinaceum* (Supplementary Fig. 15)—a widely used system for studying C_4_ photosynthesis, where the expression of C_4_-related genes typically increases from the base to the tip of the leaf^10^. Anatomical analysis revealed that *T. arundinaceum* bundle sheath cells exhibit classical C_4_ Kranz anatomy with centrifugal chloroplast arrangement and a clear maturation gradient across the leaf (Supplementary Fig. 14b), in line with classical NADP-ME species such as sugarcane^11^. Additionally, we identified nine core gene families comprising 27 genes related to photosynthesis pathways in *T. arundinaceum* genome (Supplementary Table 13). Among them, the expression levels of *TraPEPCK*, *TraPPDK-RP*, *TraCA1*, *TraCA2*, *TraNADP-MDH2*, *TraNADP-ME2*, *TraPEPC-k1*, *TraPEPC1*, and *TraPPDK1* showed a gradual increase from the base to the tip of the leaf (Supplementary Fig. 14c, 15 and 16), consistent with the expression patterns of C_4_-type genes in the genomes of sugarcane, sorghum, and maize^11-13^, suggesting that these nine genes may be involved in the C_4_ photosynthetic pathway of *T. arundinaceum*. To determine the C_4_ biochemical subtype, we examined the relative expression levels of key decarboxylation enzymes. *TraNADP-ME2* exhibited substantially higher expression than *TraPEPCK*, *TraNAD-ME1* and *TraNAD-ME2* (Supplementary Fig. 14c). Moreover, phylogenetic analysis revealed that the ortholog of *ZmPEPCK1* (C_4_) was absent in *T. arundinaceum*, and *TraPEPCK* displayed a significantly lower expression level than *ZmPEPCK1* (C_4_), similar to that found in sugarcane and sorghum (Supplementary Fig. 16g). Therefore, we propose that NADP-ME is the major decarboxylating enzyme that contributes to the C_4_ decarboxylation reaction of *T. arundinaceum*, which is in line with classical NADP-ME species such as sugarcane, *sorghum*, and maize^11-13^. Furthermore, the orthologous genes *TraPPDK-RP* and *SsPPDK-RP1* showed opposite expression patterns in leaves during the diurnal cycles (Supplementary Fig. 17), suggesting divergence in C_4_ photosynthesis pathways between *T. arundinaceum* and sugarcane.

Dehydration responsive element binding proteins/C-repeat binding factors (DREBs) play a crucial role in the transcriptional activation of the abscisic acid (ABA)-independent drought stress response. They achieve this by binding to dehydration-responsive elements (DREs) with a core motif of A/GCCGAC located in the promoters of drought-responsive genes^14-16^. We identified 18 *DREBs*, including 11 *DREB1* genes and 7 *DREB2* genes, in the genome of *T. arundinaceum* (Supplementary Fig. 18; Supplementary Table 14 and Supplementary Data 2). Expression profiling showed that different *DREB* genes exhibited variable expression levels across the different tissues (Supplementary Fig. 19a). Eight *TraDREB* genes (*TraDREB1B*, *TraDREB1C*, *TraDREB1D*, *TraDREB1E*, *TraDREB1G*, *TraDREB1H*, *TraDREB1J*, and *TraDREB2B*) showed a gradual increase in expression from the base to tip of the leaf, whereas the transcript abundance of *TraDREB2A* gradually decreased across the same gradient, suggesting these nine *TraDREBs* may play a role in photosynthesis regulation (Supplementary Fig. 19b). To assess the transcriptional response of *TraDREBs* under drought stress, we examined the expression of six *TraDREBs* (*TraDREB1B*, *TraDREB1C*, *TraDREB1J*, *TraDREB2A*, *TraDREB2B*, and *TraDREB2C*), which are orthologs of genes known to participate drought response in other grasses such as sugarcane^17^, rice^18^, and maize^19^ (Supplementary Table 15). Under both 20% (w/v) PEG 6000 and dehydration treatments, five of these six genes exhibited significant altered expression relative to the 0-hour control, while *TraDREB2C* was undetectable under PEG 6000 treatment (unpaired two-tailed Welch's *t-*test, *P* < 0.05) (Supplementary Fig. 20). Notably, *TraDREB2B* showed clearly much higher during the day compared to nighttime, whereas the remaining *TraDREBs* showed low or no differential expression during the diurnal cycle (Supplementary Figs. 19c and 21), implying that the induction of *TraDREB2B* under drought stress may be influenced by light rather than representing a specific role in the drought response of *T. arundinaceum*. Moreover, previous study has shown that ectopic expression of a *T. arundinaceum DREB2B* gene—an ortholog of *TraDREB2A* in this study—driven by the stress-inducible *RD29A* promoter, confers drought tolerance and improves yield in sugarcane under field conditions^20^. Collectively, we speculate that *TraDREB2A*, *TraDREB1B*, *TraDREB1C*, and *TraDREB1J* are likely involved in the drought stress response of *T. arundinaceum*, whereas the up-regulation of *TraDREB2B* may be partially light-induced and *TraDREB2C* shows treatment-specific expression. In future studies, to further validate the functional roles of *TraDREB* genes in drought resistance, we will include a “well-watered” control maintained throughout the experiment, as well as the measurement of physiology indicators linked to drought stress to account for circadian rhythms and other confounding factors.

**Supplementary Fig. 1. Estimation of *T. arundinaceum* Hainan92-105 genome size based on a genomic survey of 21-mer frequency distribution.**

**Supplementary Fig. 2. Genome-wide chromatin interactions in the *T. arundinaceum* Hainan92-105 genome. a** Whole genome. **b** Each chromosome.

**Supplementary Fig. 3. Comparative genomic synteny analysis of *T. arundinaceum* with *Saccharum*. a** *S. spontaneum* AP85-441. **b** *S. spontaneum* Np-X. **c** *S. officinarum* ZG1. **d** *S. officinarum* LA-purple.

**Supplementary Fig. 4. Comparison of monoploid genome size of** ***T. arundinaceum* and *Saccharum*.** The monoploid genome size was calculated by dividing the total genome size by the number of homologous chromosomes (*n*): *n* = 8 for SoFG, 8 for SoLA, 4 for SsAP, 4 for SsNp, 6 for Tra. SoZG, *S. officinarum* ZG1; SoLA, *S. officinarum* LA-purple; SsAP, *S. spontaneum* AP85-441; SsNp, *S. spontaneum* Np-X; Tra, *T. arundinaceum*. Source data are provided as a Source Data file.

**Supplementary Fig. 5. Gene number statistics for each chromosome of *T. arundinaceum* Hainan92-105.** Letters A-F represent six homologous chromosomes.

**Supplementary Fig. 6. Differential expression analysis of homologues groups in *T. arundinaceum* Hainan92-105.** S, B, H, and M indicate the seedling stage, booting, heading, and mature stages, respectively. Letters A-F represent six homologous chromosomes in each group. Statistical significance was assessed by one-way ANOVA (*F*(9, 1061876) = 128.723, *P* < 2.0 × 10⁻¹⁶), followed by pairwise comparisons using Tukey's honestly significant difference (HSD) test with adjustment for multiple comparisons. ***adjusted *P* < 0.001. Exact adjusted *P*-values and source data are provided as a Source Data file.

| **a** |  |
| --- | --- |
| **b** |  |

**Supplementary Fig. 7. Comparison of neutral and non-neutral genes among different tissues.** **a** Comparison of neutral genes among different tissues. **b** Comparison of non-neutral genes among different tissues.

**Supplementary Fig. 8. Functional enrichment analysis of shared non-neutral genes in *T. arundinaceum*.** Fisher’s exact test, with *P* values adjusted using the Benjamini-Hochberg correction for multiple hypothesis testing.

**Supplementary Fig. 9. Evolution of the *T. arundinaceum* genome. a** Analysis of intact LTR-RT insertion times in the genomes of representative grass species. **b** Phylogenetic tree showing the evolutionary relationships of *T. arundinaceum* and related species. Numbers of expanded and contracted gene families are shown in cerulean and magenta, respectively. Divergence times between species are shown in blue, and the numbers in parentheses indicate the range of divergence time. **c** Venn diagram of orthologs in *T. arundinaceum* and related species. **d** Gene ontology (GO) enrichment analysis of significantly expanded gene families in the genome of *T. arundinaceum*. Fisher’s exact test, with *P* values adjusted by the Benjamini-Hochberg method for multiple testing. **e** *K*_s_ values for gene alleles in *T. arundinaceum*. The distribution of allelic *K*_s_ in each homologous group is represented as a box plot. The centerline in each box represents the median; the lower and upper hinges represent the 25th and 75th percentiles, respectively. The numbers of gene allele pairs from Chr01 to Chr10 are as follows: *n* =  56,160, 40,861, 45,184, 35,760, 18,980, 28,097, 22,916, 16,726, 25,340, 26,512. Significance of pairwise comparisons was determined by the Wilcoxon rank sum test (*W* = 5,486,099,330, *P* < 2.20 × 10^-16^). The 95% CI of the location shift was [0.001468, 0.001848], and the effect size (*r*) was 0.0516. Source data are provided as a Source Data file.

**Supplementary Fig. 10. DNA methylation regulates expression of genes located on the paleo-duplicated chromosome pairs (PdCPs). a** Proportion of transposon elements (TE) on PdCPs and other chromosomes. **b** Expression levels of genes on PdCPs and other chromosomes in leaves. Data are presented as mean values ± SD. **c** Distribution of expression levels near TEs. The dashed lines represent the start site and end site of TE, respectively. **d** The average levels of CG, CHG, and CHH methylation on PdCPs and other chromosomes. **e** Overall distribution of methylation levels around TEs on PdCPs and other chromosomes. The dashed lines represent the start site and end site of TE, respectively. In boxplots, the centerline represents the median; the lower and upper hinges represent the 25th and 75th percentiles, respectively; and the whiskers represent 1.5× the interquartile range. *n*, the number of homologous chromosomes of Chr05, Chr08, and Others: *n* = 6 for Chr05, 6 for Chr08, 48 for Other. Pairwise comparisons were assessed using Dunn's test following a significant Kruskal–Wallis test (**a**: *H*(2) = 17.01, *P* = 2.03 × 10^-04^; **b**: *H*(2) = 2705.98, *P* = 0.00; **d**: CG, *H*(2) = 19.30, *P* = 6.43 × 10^-05^, CHG, *H*(2) = 10.31, *P* = 5.78 × 10^-03^, CHH, *H*(2) = 11.96, *P* = 2.53 × 10^-03^). *P*-values were adjusted for multiple comparisons using the Benjamini–Hochberg method. Exact *Z*-statistics, *P*-values, effect size and source data are provided as a Source Data file.

**Supplementary Fig. 11. The average methylation levels around TEs in the genome of *T. arundinaceum*.** The dashed lines represent the start site and end site of TE, respectively. Source data are provided as a Source Data file.

**Supplementary Fig. 12. Linear regression of CG, CHG, CHH methylation levels and expression levels of genes near transposon elements.** The shadow of linear regression denotes the 95% confidence interval. Spearman’s correlation. Source data are provided as a Source Data file.

**Supplementary Fig. 13. The comparison of key gene families between *T. arundinaceum* Hainan92-105 and other grass species.** Os, *O. sativa*; Zm, *Z. mays*; Tra, *T. arundinaceum* Hainan92-105; Sb, *S. bicolor*; So, *S. officinarum* LA-purple; Np, *S. spontaneum* Np-X; AP, *S. spontaneum* AP85-441.

**Supplementary Fig. 14. Photosynthetic parameters, leaf anatomical structure, and expression patterns of C_4_ pathway genes along the leaf gradient. a** The response of net CO_2_ assimilation rate to intercellular CO_2_ concentration in *T. arundinaceum* Hainan92-105 and *S*. hybrid XTT22 (mean ± SD). A: net photosynthetic rate, Ci: intercellular CO_2_ concentration. **b** The anatomical features of *T. arundinaceum* leaf. M, mesophyll cell; BS, bundle sheath cell in leaf. Scale bars, 50 μm. **c** Expression analysis of C_4_ pathway genes across leaf gradients in *T. arundinaceum*. Source data are provided as a Source Data file.

**Supplementary Fig. 15. A 9-day-old *T. arundinaceum* Hainan92-105 seedling. a, b** Ligules from leaf 1 and leaf 2 were used as markers to define developmental zones. Scale bars, 1 cm. **c** Development gradient analysis of leaf 2.

| **a** |  | **b** |  |
| --- | --- | --- | --- |
| **c** |  | **d** |  |
| **e** |  | **f** |  |
| **g** |  | **h** |  |
| **i** |  | | |

**Supplementary Fig.** **16. Phylogeny and expression analysis of C_4_ pathway genes. a** *NADP-ME*. **b** *NADP-MDH*. **c** *NADP-ME*. **d** *PEPC*. **e** *PEPC-k*. **f** *CA*. **g** *PEPCK*. **h** *PPDK*. **i** *PPDK-RP*. Tra, *T. arundinaceum*; Ss, *S. spontaneum*; Sb, *S. bicolor*; Zm, *Zea mays*; Cr, *Chlamydomonas reinhardtii*. The red clades indicated the C_4_ genes.

| **a** |  | | | | |
| --- | --- | --- | --- | --- | --- |
| **b** |  | **c** |  | **d** |  |
| **e** |  | **f** |  | **g** |  |
| **h** |  | **i** |  | **j** |  |

**Supplementary Fig. 17.** **Expression pattern of C_4_ pathway genes at different periods in leaves. a** Expression pattern of C_4_ pathway genes of *T. arundinaceum*. **b-j** Comparison analysis of the expression pattern of C_4_ pathway genes between *T. arundinaceum* and *S. spontaneum*. Tra, *T. arundinaceum*; Ss, *S. spontaneum***.** Source data are provided as a Source Data file.

**Supplementary Fig. 18. Phylogenetic tree of *DREB1* and *DREB2* genes in different species.** Purple and green clade indicate the *DREB1*-type and *DREB2*-type genes, respectively.

**Supplementary Fig. 19.** **Heat maps of *TraDREB* gene expression levels in *T. arundinaceum* Hainan92-105. a** Expression patterns of *TraDREBs* in different tissues of different stages. **b** Expression patterns of *TraDREBs* across leaf gradients. **c** Expression patterns of *TraDREBs* at different time periods in leaves. Source data are provided as a Source Data file.

**Supplementary Fig. 20. Relative expression levels of six *TraDREB* genes in *T. arundinaceum* under normal and drought conditions.** The figure integrates results from two independent drought-stress experiments: RT-qPCR analysis of plants removed from the nutrient solution (left panels) and RNA-seq analysis of plants treated with 20% (w/v) PEG 6000 (right panels). RT-qPCR values (mean ± SEM, *n* = 3 biological replicates, each with 3 technical replicates) were normalized to internal reference genes. Data are presented as fold change relative to 0 h using the 2^–ΔΔCt^ method. RNA-seq expression levels are shown as Transcripts Per Million (TPM; mean ± SEM, *n* = 3 biological replicates). Expression levels at all time points were normalized relative to the 0 h control sample (CK, collected before stress treatment). Statistical significance of each time point versus 0 h was assessed by unpaired two-tailed Welch's *t*-test. **P* < 0.05, ***P* < 0.01 vs. 0 h. Exact *t*-statistics, *P*-values, effect size and source data are provided as a Source Data file.

**Supplementary Fig. 21. Diurnal expression patterns of six *TraDREB* genes.** The x-axis represents time points at 2-hour intervals over a 24-hour period, and the y-axis represents the TPM (Transcripts Per Million) values of the genes.

**Supplementary Fig. 22. Genome-wide distribution of genetic variants.** Circular plot showing the monoploid genome assembly (in Mbp) on the outermost ring (**a**), followed by SNP density (**b**) and Indel density (**c**) across haplotypes. From outermost to innermost, each colored line represents a distinct haplotype: A, B, C, D, E, and F, respectively. Source data are provided as a Source Data file.

**Supplementary Fig. 23.** **Population genomics analysis of *T. arundinaceum* accessions.** **a** STRUCTURE analysis of re-sequenced populations with *K* values from 2 to 6. **b** Plot of ADMIXTURE cross-validation errors for *K* = 1 to 10. **c**, Potential geographic distribution of *T. arundinaceum*. Ecological niche modeling was used to predict potential distribution patterns under current climate conditions (1970-2000). Colors on the map indicate the relative density of *T. arundinaceum* throughout its distribution range. The base map was downloaded from the Standard Map Service System (<http://bzdt.ch.mnr.gov.cn/>; No. GS(2016)1666). Source data are provided as a Source Data file.

**Supplementary Fig. 24. The distribution of Tajima’D values among *T. arundinaceum* accessions.** Source data are provided as a Source Data file.

**Supplementary Fig. 25. GO enrichment analysis of genes carrying deleterious mutations in *T. arundinaceum*.** Fisher’s exact test, with *P*-values adjusted using the Benjamini-Hochberg correction for multiple hypothesis testing.

**Supplementary Fig. 26. Genome-wide distribution of 7,592 core adaptive variants identified by both RDA and LFMM.** Circular plot showing from outermost to innermost rings: (**a**) monoploid genome assembly (in Mbp); (**b**) genomic locations of adaptive SNPs across haplotypes A through F. Source data are provided as a Source Data file.

**Supplementary Fig. 27. GO enrichment analysis of genes with core adaptive variants.** Fisher’s exact test, with *P*-values adjusted using the Benjamini-Hochberg correction for multiple hypothesis testing.

**Supplementary Fig. 28. Linkage disequilibrium and allele frequency–environment associations of candidate genes *CYP78A5* and *bHLH112*. a, b** Heatmap of linkage disequilibrium surrounding *CYP78A5* (**a**) and *bHLH112* (**b**), corresponding to the two candidate genes shown in Fig. 6. **c, d** Correlations between allele frequencies of selected candidate variants Chr02C_112553480 (**c**) and Chr07A_30751379 (**d**) and environmental variables. The shadow of linear regression denotes the 95% confidence interval. Two-sided Student’s *t*-test. Source data are provided as a Source Data file.

**Supplementary Fig. 29. Display of some genes significantly related to BIO2 (mean diurnal range). a** Manhattan plot shows the genotype-environment association of core adaptive variants. SNPs and Indels are represented by points and triangles, respectively. Dashed horizontal lines represent significance thresholds (gray represents the Benjamini–Hochberg adjusted FDR threshold, FDR = 0.01; blue represents the Bonferroni correction, corrected *P* = 0.01). Selected candidate genes are labeled in the plot at their respective genomic positions. **b, c** Upper panels: schematic diagram of the genes associated with the variant marked in **a**. Lower panels: distribution of allele frequencies across 10 populations of the selected variant (left panels). Colors of raster on map represent the environmental variable under current scenario. The base map was downloaded from the Standard Map Service System (http://bzdt.ch.mnr.gov.cn/; No. GS(2020)4619). Linear regression of allele frequencies and environment variables for the selected variant (right panels). The shadow of linear regression denotes the 95% confidence interval. Two-sided Student’s *t*-test. Source data are provided as a Source Data file.

**Supplementary Fig. 30. Display of some genes significantly related to BIO7 (Temperature Annual Range). a** Manhattan plot shows the genotype-environment association of core adaptive variants. SNPs and Indels are represented by points and triangles, respectively. Dashed horizontal lines represent significance thresholds (gray represents the Benjamini–Hochberg adjusted FDR threshold, FDR = 0.01; blue represents the Bonferroni correction, corrected *P* = 0.01). Selected candidate genes are labeled in the plot at their respective genomic positions. **b-g** Upper panels: schematic diagram of the genes associated with the variant marked in **a**. Lower panels: distribution of allele frequencies across 10 populations of the selected variant (left panels). Colors of raster on map represent the environmental variable under current scenario. The base map was downloaded from the Standard Map Service System (<http://bzdt.ch.mnr.gov.cn/>; No. GS(2020)4619). Linear regression of allele frequencies and environment variables for the selected variant (right panels). The shadow of linear regression denotes the 95% confidence interval. Two-sided Student’s *t*-test. Source data are provided as a Source Data file.

**Supplementary Fig. 31. Display of some genes significantly related to BIO13 (precipitation of wettest month). a** Manhattan plot shows the genotype-environment association of core adaptive variants. SNPs and Indels are represented by points and triangles, respectively. Dashed horizontal lines represent significance thresholds (gray represents the Benjamini–Hochberg adjusted FDR threshold, FDR = 0.01; red represents the Bonferroni correction, corrected *P* = 0.01). Selected candidate genes are labeled in the plot at their respective genomic positions. **b** Upper panels: schematic diagram of the genes associated with the variant marked in **a**. Lower panels: distribution of allele frequencies across 10 populations of the selected variant (left panels). Colors of raster on map represent the environmental variable under current scenario. The base map was downloaded from the Standard Map Service System (<http://bzdt.ch.mnr.gov.cn/>; No. GS(2020)4619). Linear regression of allele frequencies and environment variables for the selected variant (right panels). The shadow of linear regression denotes the 95% confidence interval. Two-sided Student’s *t*-test. Source data are provided as a Source Data file.

**Supplementary Fig. 32. Display of some genes significantly related to BIO15 (precipitation seasonality). a** Manhattan plot shows the genotype-environment association of core adaptive variants. SNPs and Indels are represented by points and triangles, respectively. Dashed horizontal lines represent significance thresholds (gray represents the Benjamini–Hochberg adjusted FDR threshold, FDR = 0.01; red represents the Bonferroni correction, corrected *P* = 0.01). Selected candidate genes are labeled in the plot at their respective genomic positions. **b-e** Upper panels: schematic diagram of the genes associated with the variant marked in **a**. Lower panels: distribution of allele frequencies across 10 populations of the selected variant (left panels). Colors of raster on map represent the environmental variable under current scenario. The base map was downloaded from the Standard Map Service System (<http://bzdt.ch.mnr.gov.cn/>; No. GS(2020)4619). Linear regression of allele frequencies and environment variables for the selected variant (right panels). The shadow of linear regression denotes the 95% confidence interval. Two-sided Student’s *t*-test. Source data are provided as a Source Data file.

**Supplementary Fig. 33. Display of some genes significantly related to BIO19 (Precipitation of Coldest Quarter). a** Manhattan plot shows the genotype-environment association of core adaptive variants. SNPs and Indels are represented by points and triangles, respectively. Dashed horizontal lines represent significance thresholds (gray represents the Benjamini–Hochberg adjusted FDR threshold, FDR = 0.01; red represents the Bonferroni correction, corrected *P* = 0.01). Selected candidate genes are labeled in the plot at their respective genomic positions. **b-f** Upper panels: schematic diagram of the genes associated with the variant marked in **a**. Lower panels: distribution of allele frequencies across 10 populations of the selected variant (left panels). Colors of raster on map represent the environmental variable under current scenario. The base map was downloaded from the Standard Map Service System (<http://bzdt.ch.mnr.gov.cn/>; No. GS(2020)4619). Linear regression of allele frequencies and environment variables for the selected variant (right panels). The shadow of linear regression denotes the 95% confidence interval. Two-sided Student’s *t*-test. Source data are provided as a Source Data file.

**Supplementary Table 1.** **Statistics of sequencing reads used for *T. arundinaceum* Hainan92-105 genome assembly.**

| Items | Pacbio HIFI | | Illumina | Hi-C |
| --- | --- | --- | --- | --- |
|  | older | newly |  |  |
| Total Num. of sequencing Cell (Library) | 6 | 2 | 1 | 4 |
| Total Num. of reads | 6,872,879 | 14,495,947 | 2,892,343,624 | 4,257,772,728 |
| Total Num. of sequenced Base (Mb) | 123,726 | 238,152 | 433,852 | 636,864 |
| Mean reads length (bp) | 18,002 | 16,429 | 150 | 150 |
| Coverage (×) | ~19 | ~36 | ~65 | ~95 |

**Supplementary Table 2. Statistics for Hi-C sequencing and mapping for the *T. arundinaceum* Hainan92-105 genome.**

| **Statistics of mapping** | | |
| --- | --- | --- |
| Clean Paired-end Reads | 2,128,886,364 | |
| Mapped Paired-end Reads | 2,095,058,491 | |
| Mapped Ratio (%) | 98.41% | |
| Unique Mapped Paired-end Reads | 183,468,653 | |
| Unique Mapped Ratio (%) | 8.62% | |
| **Statistics of valid reads** | | |
| Unique Mapped Paired-end Reads | 183,468,653 | |
| Valid Interaction Pairs | 154,403,104 | |
| Valid Interaction Rate (%) | 84.16% | |
| Dangling End Pairs | 8,752,790 | |
| Dangling End Rate (%) | 4.77% | |
| Re-ligation Pairs | 599,096 | |
| Re-ligation Rate (%) | 0.33% | |
| Self Circle Paired-end Reads | | 837,237 |
| Self Circle Rate (%) | 0.46% | |
| Dumped Paired-end Reads | 32,124 | |
| Dumped Rate (%) | 0.02% | |

**Supplementary Table 3. Statistics of chromosome-level scaffolds based on the Hi-C reads.**

|  | Haplotype A | | Haplotype B | | Haplotype C | | Haplotype D | | Haplotype E | | Haplotype F | |
| --- | --- | --- | --- | --- | --- | --- | --- | --- | --- | --- | --- | --- |
|  | No. of contigs | Length (Mb) | No. of contigs | Length (Mb) | No. of contigs | Length (Mb) | No. of contigs | Length (Mb) | No. of contigs | Length (Mb) | No. of contigs | Length (Mb) |
| Chr01 | 11 | 133.91 | 18 | 141.71 | 11 | 142.74 | 10 | 142.34 | 10 | 145.88 | 8 | 145.77 |
| Chr02 | 27 | 121.26 | 5 | 125.43 | 13 | 118.36 | 4 | 120.72 | 13 | 124.61 | 9 | 121.24 |
| Chr03 | 4 | 118.10 | 8 | 121.89 | 9 | 114.67 | 5 | 108.89 | 10 | 111.20 | 3 | 115.47 |
| Chr04 | 5 | 101.56 | 6 | 111.22 | 6 | 104.56 | 7 | 104.81 | 25 | 99.41 | 12 | 106.87 |
| Chr05 | 12 | 119.22 | 11 | 122.11 | 21 | 112.41 | 24 | 115.10 | 21 | 129.05 | 10 | 121.54 |
| Chr06 | 4 | 97.22 | 10 | 91.82 | 26 | 91.47 | 8 | 102.84 | 14 | 94.43 | 6 | 100.29 |
| Chr07 | 7 | 95.91 | 9 | 102.97 | 7 | 100.74 | 11 | 90.62 | 7 | 94.77 | 4 | 83.70 |
| Chr08 | 2 | 84.64 | 6 | 95.58 | 6 | 83.89 | 9 | 88.42 | 5 | 88.87 | 8 | 87.58 |
| Chr09 | 6 | 92.22 | 6 | 92.66 | 5 | 92.92 | 8 | 95.51 | 4 | 95.87 | 4 | 94.98 |
| Chr10 | 7 | 94.67 | 9 | 95.95 | 12 | 109.92 | 5 | 96.05 | 5 | 102.76 | 8 | 103.86 |
| Anchored contigs | 566 | | | | | | | | | | | |
| Total no. of contigs | 2,097 | | | | | | | | | | | |
| Anchored sequences (Mb) | 6,465.19 | | | | | | | | | | | |
| Total assembled size (Mb) | 6,722.76 | | | | | | | | | | | |
| Anchor rate (%) | 96.17 | | | | | | | | | | | |

**Supplementary Table 4. Assessment of genome consistency based on Illumina and HiFi reads.**

|  | Illumina reads | HiFi reads |
| --- | --- | --- |
| Number of reads | 2,892,343,624 | 30,082,357 |
| Data size (bp) | 433,851,543,600 | 238,151,801,578 |
| mapped bases (bp) | 425,300,489,250 | 238,080,356,037 |
| Map rate (%) | 98.03 | 99.97 |
| Genome Length (bp) | 6,722,760,840 | |
| Mean Depth (×) | 63.80 | 35.39 |
| Coverage Rate (%) | 99.96 | 99.97 |

**Supplementary Table 5. Completeness analysis of genome assembly.**

| Description | BUSCO | Compleasm |
| --- | --- | --- |
| Complete BUSCOs (%) | 98.80 | 99.69 |
| Complete and single-copy BUSCOs (%) | 0.60 | 1.86 |
| Complete and duplicated BUSCOs (%) | 98.20 | 97.83 |
| Fragmented BUSCOs (%) | 0.70 | 0.12 |
| Missing BUSCOs (%) | 0.50 | 0.19 |
| Total BUSCO groups searched (N) | 1,614 | |

**Supplementary Table 6.** **Quality assessment of the gene set for the *T. arundinaceum* Hainan92-105 genome.**

| Metrics | Number | Percentage (%) |
| --- | --- | --- |
| Complete BUSCOs (C) | 1,550 | 96.10 |
| Complete and single-copy BUSCOs (S) | 51 | 3.20 |
| Complete and duplicated BUSCOs (D) | 1,499 | 92.90 |
| Fragmented BUSCOs (F) | 16 | 1.00 |
| Missing BUSCOs (M) | 48 | 2.90 |
| Total BUSCO groups searched | 1,614 |  |

**Supplementary Table 7. Gene function annotation statistics of *T. arundinaceum* Hainan92-105.**

| Type | Number | Percent (%) |
| --- | --- | --- |
| **Annotated** | 173,717 | 96.26 |
| Nr | 171,574 | 95.07 |
| Swissprot | 122,284 | 67.76 |
| Uniprot | 172,678 | 95.68 |
| eggNOG | 168,145 | 93.17 |
| GO | 60,954 | 33.78 |
| KO | 79,179 | 43.87 |
| KEGG | 60,511 | 33.53 |
| PFAM | 115,301 | 63.89 |
| **Unannotated** | 6,750 | 3.74 |
| **Total** | 180,467 | 100 |

**Supplementary Table 8. The statistic of *T. arundinaceum* Hainan92-105 gene alleles annotation.**

| Chromosome | Total no. of genes | No. of genes with 6 alleles | No. of genes with 5 alleles | No. of genes with 4 alleles | No. of genes with 3 alleles | No. of genes with 2 alleles | No. of genes with 1 allele | No. of tandem duplicated gene | No. of dispersely duplicated genes |
| --- | --- | --- | --- | --- | --- | --- | --- | --- | --- |
| Chr01 | 5,801 | 2,919 | 907 | 432 | 365 | 425 | 753 | 150 | 2,023 |
| Chr02 | 4,718 | 2,083 | 669 | 404 | 369 | 423 | 770 | 121 | 2,099 |
| Chr03 | 4,721 | 2,314 | 767 | 375 | 309 | 314 | 642 | 119 | 1,955 |
| Chr04 | 3,839 | 1,799 | 657 | 292 | 250 | 299 | 542 | 78 | 1,443 |
| Chr05 | 3,357 | 823 | 436 | 336 | 398 | 461 | 903 | 166 | 2,621 |
| Chr06 | 3,181 | 1,424 | 504 | 270 | 234 | 260 | 489 | 74 | 1,585 |
| Chr07 | 3,014 | 1,173 | 391 | 272 | 283 | 317 | 578 | 84 | 1,707 |
| Chr08 | 2,446 | 819 | 323 | 236 | 222 | 241 | 605 | 55 | 1,542 |
| Chr09 | 2,895 | 1,361 | 374 | 215 | 237 | 239 | 469 | 88 | 1,085 |
| Chr10 | 3,269 | 1,332 | 497 | 305 | 285 | 290 | 560 | 82 | 1,744 |
| Total | 37,241 | 16,047 | 5,525 | 3,137 | 2,952 | 3,269 | 6,311 | 1,017 | 17,804 |

**Supplementary Table 9. Non-neutral and neutral pattern genes in examined tissues.**

| Tissue | Type | Number | Total | Percent |
| --- | --- | --- | --- | --- |
| Seedling leaf | neutral | 5,336 | 7,655 | 69.71% |
|  | non-neutral | 2,319 |  | 30.29% |
| Seedling steam | neutral | 6,560 | 9,124 | 71.90% |
|  | non-neutral | 2,564 |  | 28.10% |
| Seedling root | neutral | 5,683 | 8,154 | 69.70% |
|  | non-neutral | 2,471 |  | 30.30% |
| Booting-flower | neutral | 6,413 | 8,857 | 72.41% |
|  | non-neutral | 2,444 |  | 27.59% |
| Heading-flower | neutral | 5,625 | 8,349 | 67.37% |
|  | non-neutral | 2,724 |  | 32.63% |
| Mature-flower | neutral | 5,996 | 8,469 | 70.80% |
|  | non-neutral | 2,473 |  | 29.20% |

**Supplementary Table 10. Statistic of transposable elements in *T. arundinaceum* Hainan92-105 genome.**

|  | Count | Length (bp) | % of genome | % of repeats |
| --- | --- | --- | --- | --- |
| **Total repeat fraction** | 5,373,120 | 4,767,783,111 | 70.92 | 100 |
| **Class I: Retroelement** | 2,603,419 | 3,346,389,056 | 49.78 | 70.19 |
| **LTR Retrotransposon** |  |  |  |  |
| Copia | 602,482 | 560,457,654 | 8.34 | 11.76 |
| Gypsy | 1,761,027 | 2,672,311,274 | 39.75 | 56.05 |
| Other | 29,405 | 6,703,566 | 0.10 | 0.14 |
| **Non-LTR Retrotransposon** |  |  |  |  |
| LINE | 210,505 | 106,916,562 | 1.59 | 2.24 |
| SINE | 0 | 0 | 0 | 0 |
| **Class II: DNA transposon** | 1,607,956 | 510,867,176 | 7.60 | 10.71 |
| hobo-Activator | 173,312 | 50,344,875 | 0.75 | 1.06 |
| Tc1-IS630-Pogo | 295,742 | 55,749,146 | 0.83 | 1.17 |
| MULE-MuDR | 295,612 | 95,829,974 | 1.43 | 2.01 |
| Tourist/Harbinger | 273,339 | 61,816,521 | 0.92 | 1.30 |
| Other | 569,951 | 247,126,660 | 3.67 | 5.17 |
| **Helitron** | 164,334 | 41,952,318 | 0.62 | 0.88 |
| **Unclassified** | 978,090 | 391,847,897 | 5.83 | 8.22 |
| **Tandem Repeat** | 19,321 | 476,726,664 | 7.09 | 10.00 |

**Supplementary Table 11. Comparative analysis of genomic repeat sequences between *T. arundinaceum* Hainan92-105 and related species.**

|  | AP85-441 | Np-X | SoLA | SoZG | Er | Ms | Sb | Tra |
| --- | --- | --- | --- | --- | --- | --- | --- | --- |
| **Total repeat fraction (%)** | 59.33 | 60.61 | 64.19 | 64.77 | 63.54 | 62.53 | 68.36 | 70.92 |
| **Class I: Retroelement (%)** | 43.91 | 44.52 | 49.21 | 49.19 | 47.89 | 47.03 | 52.16 | 49.78 |
| **LTR Retrotransposon** | 41.94 | 42.26 | 47.12 | 47.14 | 46.00 | 45.94 | 51.02 | 48.19 |
| Copia (%) | 14.54 | 11.96 | 16.29 | 16.4 | 8.13 | 12.13 | 6.79 | 8.34 |
| Gypsy (%) | 27.30 | 30.12 | 30.66 | 30.49 | 37.77 | 33.67 | 44.13 | 39.75 |
| Other (%) | 0.10 | 0.18 | 0.17 | 0.25 | 0.10 | 0.14 | 0.10 | 0.10 |
| **Non-LTR Retrotransposon** | 1.97 | 2.26 | 2.09 | 2.05 | 1.89 | 1.09 | 1.14 | 1.59 |
| LINE (%) | 1.96 | 2.25 | 2.09 | 2.05 | 1.88 | 1.05 | 1.14 | 1.59 |
| SINEs (%) | 0.01 | 0.01 | 0 | 0 | 0.01 | 0.04 | 0 | 0 |
| **Class II: DNA transposon (%)** | 9.68 | 9.42 | 9.08 | 9.48 | 10.63 | 9.32 | 9.72 | 7.60 |
| hobo-Activator (%) | 1.30 | 1.16 | 1.08 | 1.02 | 0.83 | 0.71 | 0.70 | 0.75 |
| Tc1-IS630-Pogo (%) | 0.88 | 1.02 | 0.72 | 0.75 | 0.87 | 0.52 | 0.76 | 0.83 |
| MULE-MuDR (%) | 2.18 | 1.94 | 1.62 | 2.21 | 1.83 | 1.97 | 1.01 | 1.43 |
| Tourist/Harbinger (%) | 2.04 | 2.28 | 2.30 | 2.19 | 1.83 | 1.18 | 2.90 | 0.92 |
| Other (%) | 3.28 | 3.02 | 3.36 | 3.31 | 5.27 | 4.94 | 4.35 | 3.67 |
| **Helitron (%)** | 0.88 | 0.61 | 0.47 | 0.49 | 0.76 | 0.6 | 0.48 | 0.62 |
| **Unclassified (%)** | 3.59 | 3.13 | 2.63 | 2.94 | 4.27 | 4.87 | 0.88 | 5.83 |
| **Repeat_region (%)** | 1.27 | 2.92 | 2.80 | 2.66 | 0 | 0.72 | 5.12 | 7.09 |

Note: AP85-441 and Np-X, *S. spontaneum*; SoLA, *S. officinarum* LA-purple; SoZG, *S. officinarum* ZG1; Er, *E. rufipilus*; Ms, *M.* *sinensis*; Sb, *S. bicolor*; Tra, *T. arundinaceum*.

**Supplementary Table 12. Photosynthetic parameters of CO_2_ response curves.**

| **Species** | **Vpmax** | **Vcmax** | **Rd** | **Rm** |
| --- | --- | --- | --- | --- |
| ***T. arundinaceum*** | 76.01 | 48.33 | 0.48 | 0.24 |
| ***S.* hybrid** | 66.91 | 52.54 | 0.53 | 0.26 |

Note: Vpmax, maximal PEP carboxylation activity; Vcmax, maximal Rubisco activity; Rd, leaf mitochondrial respiration; Rm, mesophyll mitochondrial respiration.

**Supplementary Table 13. The information of the C_4_ enzyme genes and their non-C_4_ isoforms genes in the *T. arundinaceum* Hainan92-105 genome.**

| *S. bicolor* | *T. arundinaceum* | | | | *S. spontaneum* |
| --- | --- | --- | --- | --- | --- |
|  | Gene ID | Name | Length (aa) | Idenity (%) |  |
| *SbCA1** | *Tra_03A00019760* | *TraCA1** | 446 | 94.21 | *SsCA1** |
| *SbCA2** | *Tra_03B00020650* | *TraCA2** | 203 | 95.05 | *SsCA2** |
| *SbCA3* | *Tra_03A00019770* | *TraCA3* | 202 | 98.02 | *SsCA3* |
| *SbCA4* | *Tra_03D00019080* | *TraCA4* | 249 | 91.02 | *SsCA4* |
| *SbPEPC1** | *Tra_10B00014500* | *TraPEPC1** | 961 | 94.59 | *SsPEPC1** |
| *SbPEPC2* | *Tra_02B00014810* | *TraPEPC2a* | 967 | 99.38 | *SsPEPC2* |
| *SbPEPC3* | *Tra_03C00026360* | *TraPEPC3* | 966 | 98.55 | *SsPEPC3* |
| *SbPEPC4* | *Tra_04A00009390* | *TraPEPC4* | 960 | 99.06 | *SsPEPC4* |
| *SbPEPC5* | *Tra_07C00011610* | *TraPEPC5* | 964 | 99.17 | *SsPEPC5* |
| *SbPEPC-k1** | *Tra_04B00029560* | *TraPEPC-k1** | 285 | 95.05 | *SsPEPC-k1** |
| *SbPEPC-k2* | *Tra_04F00018100* | *TraPEPC-k2* | 307 | 91.53 | *SsPEPC-k2* |
| *SbPEPC-k3* | *Tra_06A00012610* | *TraPEPC-k3a* | 297 | 97.98 | *SsPEPC-k3* |
|  | *Tra_06A00012620* | *TraPEPC-k3b* | 299 | 96.99 |  |
| *SbNADP-MDH1* | *Tra_07E00015310* | *TraNADP-MDH1* | 438 | 95.44 | *SsNADP-MDH1* |
| *SbNADP-MDH2** | *Tra_07B00016130* | *TraNADP-MDH2** | 435 | 95.40 | *SsNADP-MDH2** |
| *SbNADP-ME1* | *Tra_09B00009450* | *TraNADP-ME1* | 646 | 95.86 | *SsNADP-ME1* |
| *SbNADP-ME2** | *Tra_03B00003420* | *TraNADP-ME2** | 639 | 95.31 | *SsNADP-ME2** |
| *SbNADP-ME3* | *Tra_03C00025330* | *TraNADP-ME3* | 653 | 97.98 | *SsNADP-ME3* |
| *SbNADP-ME4* | *Tra_09A00006860* | *TraNADP-ME4* | 570 | 96.49 | *SsNADP-ME4* |
| *SbNADP-ME5* | *Tra_03D00023210* | *TraNADP-ME5* | 637 | 99.33 | *SsNADP-ME5* |
| *SbNADP-ME6* | *Tra_03A00003430* | *TraNADP-ME6* | 648 | 80.57 | *SsNADP-ME6a* |
|  |  |  |  |  | *SsNADP-ME6b* |
| *SbPPDK1** | *Tra_09A00011350* | *TraPPDK1** | 947 | 96.52 | *SsPPDK1** |
| *SbPPDK2* | *Tra_01F00028830* | *TraPPDK2* | 894 | 95.71 | *SsPPDK2* |
| *SbPPDK-RP1** | *Tra_02C00027760* | *TraPPDK-RP** | 437 | 85.75 | *SsPPDK-RP1** |
| *SbPPDK-RP2* |  |  |  | 86.28 | *SsPPDK-RP2* |
| *SbPPDK-RP3* |  |  |  | 88.42 | *SsPPDK-RP3* |
| *SbPEPCK* | *Tra_01C00037900* | *TraPEPCK* | 659 | 99.24 | *SsPEPCK* |
| *SbNAD-ME1* | *Tra_02B00027060* | *TraNAD-ME1* | 622 | 99.52 | *SsNAD-ME1* |
| *SbNAD-ME2* | *Tra_01D00017920* | *TraNAD-ME2* | 618 | 99.19 | *SsNAD-ME2* |

Note: * represents the C_4_ gene.

**Supplementary Table 14. The number of *DREB* genes in the genome of different species.**

| Species | *DREBs*/*CBFs* | | Total |
| --- | --- | --- | --- |
|  | *DREB1* | *DREB2* |  |
| *A. thaliana* | 6 | 9 | 15 |
| *Z. mays* | 10 | 10 | 20 |
| *O. sativa* | 10 | 6 | 16 |
| *S. bicolor* | 10 | 7 | 17 |
| *S.* *rufipilus* | 10 | 6 | 16 |
| *S. spontaneum* | 12 (19) | 6 (10) | 18 (29) |
| *T. arundiaceum* | 11 (66) | 7 (43) | 18 (109) |

Note: The numbers in parentheses indicate the number of alleles.

**Supplementary Table 15. Orthologs of drought stress-related *DREB* genes in *T. arundinaceum* Hainan92-105.**

| *T. arundinaceum* | orthologous in other species | |
| --- | --- | --- |
| Gene | Gene | Similarities (%) |
| *TraDREB1C* | *SsDREB1F* | 88.02 |
| *TraDREB1J* | *SsDREB1L* | 84.62 |
|  | *ZmDREB1.7* | 67.45 |
| *TraDREB1B2* | *ZmDREB1.1* | 82.16 |
| *TraDREB2C* | *ZmDREB2.8* | 77.56 |
| *TraDREB2B* | *SsDREB2F* | 89.43 |
|  | *OsDREB2B* | 51.96 |
| *TraDREB2A* | *SsDREB2D* | 98.31 |
|  | *OsDREB2A* | 67.79 |

**Supplementary Table 16. Environmental variables used in this study derived from WorldClim.**

| Code | Variable |
| --- | --- |
| **Temperature-related** | |
| BIO1 | Annual Mean Temperature |
| BIO2 | Mean Diurnal Range |
| BIO3 | Isothermality (BIO2/BIO7) |
| BIO4 | Temperature Seasonality |
| BIO5 | Max Temperature of Warmest Month |
| BIO6 | Min Temperature of Coldest Month |
| BIO7 | Temperature Annual Range (BIO5-BIO6) |
| BIO8 | Mean Temperature of Wettest Quarter |
| BIO9 | Mean Temperature of Driest Quarter |
| BIO10 | Mean Temperature of Warmest Quarter |
| BIO11 | Mean Temperature of Coldest Quarter |
| **Precipitation-related** | |
| BIO12 | Annual Precipitation |
| BIO13 | Precipitation of Wettest Month |
| BIO14 | Precipitation of Driest Month |
| BIO15 | Precipitation Seasonality |
| BIO16 | Precipitation of Wettest Quarter |
| BIO17 | Precipitation of Driest Quarter |
| BIO18 | Precipitation of Warmest Quarter |
| BIO19 | Precipitation of Coldest Quarter |

**Supplementary Table 17. Isolation-by-distance (IBD) and isolation-by-environment (IBE) analyses of potential adaptive and neutral variants.**

| Test | Parameters | Adaptive loci | |  | Neutral loci | |
| --- | --- | --- | --- | --- | --- | --- |
|  |  | *r* | *P* |  | *r* | *P* |
| Mantel | IBD：Gen vs. Geo | 0.6571 | 0.001*** |  | 0.7108 | 0.001*** |
|  | IBE：Gen vs. Env | 0.6427 | 0.001*** |  | 0.5731 | 0.005*** |
| Partial Mantel | Gen vs. Geo \| Env | 0.561 | 0.002*** |  | 0.635 | 0.003** |
|  | Gen vs. Env \| Geo | 0.5405 | 0.002** |  | 0.4358 | 0.022* |

Note: Gen, genetic distance (*F*_ST_); Geo, geographic distance; Env, environmental distance; partial Mantel tests (two-sided): X ~ Y(Z) is the correlation between X and Y matrices, controlling for Z. Asterisks indicate statistical significance (**P* ≤ 0.05, ***P* ≤ 0.01, ****P* ≤ 0.001).

**Supplementary Table 18. The relative contribution of environment and geography in shaping adaptive and neutral genetic variation.**

| Partial RDA models | Adaptive loci | | |  | Neutral loci | | |
| --- | --- | --- | --- | --- | --- | --- | --- |
|  | R^2^ (adjust) | F | *P* (>F) |  | R^2^ (adjust) | F | *P* (>F) |
| Combined fractions |  |  |  |  |  |  |  |
| F ~ Env. | 0.248453 | 8.1785 | 0.001 |  | 0.06179755 | 2.4303 | 0.001 |
| F ~ Geo. | 0.1732294 | 16.924 | 0.001 |  | 0.04362917 | 4.4671 | 0.001 |
| Individual fractions |  |  |  |  |  |  |  |
| F ~ env. + Geo. | 0.2583241 | 6.8824 | 0.001 |  | 0.06712217 | 2.2152 | 0.001 |
| F ~ env. \| Geo. | 0.08509466 | 3.4586 | 0.001 |  | 0.023493 | 1.5396 | 0.001 |
| F ~ Geo. \| env. | 0.009871152 | 1.9649 | 0.001 |  | 0.005324626 | 1.4138 | 0.001 |
| Total explained | 0.353289912 |  |  |  | 0.095939796 |  |  |
| Total unexplained | 0.646710088 |  |  |  | 0.904060204 |  |  |

Note: F, dependent matrix of population minor allele frequency; Env., environmental variables; Geo., geographic variables (latitude and longitude). The significance of the partial RDA was tested using a one-tailed pseudo-F test based on 999 unrestricted permutations under the reduced model. No correction for multiple comparisons was applied.

**Supplementary Table 19. Primer sequences used for RT-qPCR validation of *TraDREB* genes expression.**

| Gene name | Forward primer | Reverse primer |
| --- | --- | --- |
| *TraDREB1C* | GTGTCCGATCAAGAAGGAGATG | TGGTGCTCTGACGAGGT |
| *TraDREB1B2* | TGGACTTGGAATTGGATGTGTA | GTCACAGCAGTCTCCATGAT |
| *TraDREB1J* | CTTGGAGTTCGATCACTCGTATTA | TCGATGTTGCTATGCCATCC |
| *TraDREB2B* | GACGCTGGAACCTATCACAAA | CCATCATCCTCAGCATCTCATC |
| *TraDREB2A* | AGAAGAGGATGAGGAGGAAGAG | ACCCTTTCTTGGAACCCTTG |
| *TraDREB2C* | GTCCAACTCCGACTCCTTT | GTCTGGTGGTGGTTGTAGTT |
| *TraeEF-1a* | AGCCCACTTTGAGACAGAAC | AGCTTGAGATGCACCACTTATC |
| *TraHIP1* | CTGCTGGTGCTTCTCATTCTA | CTGGCTGATGACTCCTAATCTC |

**Supplementary references**

1.	Simão, F.A., Waterhouse, R.M., Ioannidis, P., Kriventseva, E.V. & Zdobnov, E.M. BUSCO: assessing genome assembly and annotation completeness with single-copy orthologs. *Bioinformatics* **31**, 3210-3212 (2015).

2.	Huang, N. & Li, H. compleasm: a faster and more accurate reimplementation of BUSCO. *Bioinformatics* **39**, btad595 (2023).

3.	Zhang, J. *et al.* Allele-defined genome of the autopolyploid sugarcane *Saccharum spontaneum* L. *Nature Genetics* **50**, 1565-1573 (2018).

4.	Liang, Z. *et al.* Epigenetic Modifications of mRNA and DNA in Plants. *Molecular Plant* **13**, 14-30 (2020).

5.	Mendes, F.K., Vanderpool, D., Fulton, B. & Hahn, M.W. CAFE 5 models variation in evolutionary rates among gene families. *Bioinformatics* **36**, 5516-5518 (2020).

6.	Little, A. *et al.* Revised Phylogeny of the Cellulose Synthase Gene Superfamily: Insights into Cell Wall Evolution. *Plant Physiology* **177**, 1124-1141 (2018).

7.	Yin, Y., Huang, J. & Xu, Y. The cellulose synthase superfamily in fully sequenced plants and algae. *BMC Plant Biology* **9**, 99 (2009).

8.	Carpita, N.C. Update on mechanisms of plant cell wall biosynthesis: how plants make cellulose and other (1->4)-β-D-glycans. *Plant Physiology* **155**, 171-84 (2011).

9.	Polko, J.K. & Kieber, J.J. The Regulation of Cellulose Biosynthesis in Plants. *Plant Cell* **31**, 282-296 (2019).

10.	Li, P. *et al.* The developmental dynamics of the maize leaf transcriptome. *Nature Genetics* **42**, 1060-7 (2010).

11.	Hua, X. *et al.* Regulatory Network of the Late-Recruited Primary Decarboxylase C_4_NADP-ME in Sugarcane. *Plant Physiology* (2024).

12.	Wang, L. *et al.* Comparative analyses of C₄ and C₃ photosynthesis in developing leaves of maize and rice. *Nature Biotechnology* **32**, 1158-65 (2014).

13.	Ding, Z. *et al.* Identification of Photosynthesis-Associated C_4_ Candidate Genes through Comparative Leaf Gradient Transcriptome in Multiple Lineages of C_3_ and C_4_ Species. *PLoS One* **10**, e0140629 (2015).

14.	Yamaguchi-Shinozaki, K. & Shinozaki, K. Transcriptional regulatory networks in cellular responses and tolerance to dehydration and cold stresses. *Annual Review of Plant Biology* **57**, 781-803 (2006).

15.	Nakashima, K., Ito, Y. & Yamaguchi-Shinozaki, K. Transcriptional regulatory networks in response to abiotic stresses in *Arabidopsis* and grasses. *Plant Physiology* **149**, 88-95 (2009).

16.	Agarwal, P.K., Gupta, K., Lopato, S. & Agarwal, P. Dehydration responsive element binding transcription factors and their applications for the engineering of stress tolerance. *Journal of Experimental Botany* **68**, 2135-2148 (2017).

17.	Li, Z. *et al.* Genome-wide identification and expression profiling of *DREB* genes in *Saccharum spontaneum*. *BMC Genomics* **22**, 456 (2021).

18.	Matsukura, S. *et al.* Comprehensive analysis of rice DREB2-type genes that encode transcription factors involved in the expression of abiotic stress-responsive genes. *Molecular Genetics and Genomics* **283**, 185-96 (2010).

19.	Liu, S. *et al.* Genome-wide analysis of *ZmDREB* genes and their association with natural variation in drought tolerance at seedling stage of *Zea mays* L. *PLoS Genetics* **9**, e1003790 (2013).

20.	Xiao, S. *et al.* Field evaluation of *TaDREB2B*-ectopic expression sugarcane (*Saccharum* spp. hybrid) for drought tolerance. *Frontiers in Plant Science* **13**, 963377 (2022).
